# Alcohol consumption and its association with cancer, cardiovascular, liver and brain diseases: a systematic review of Mendelian randomization studies

**DOI:** 10.3389/fepid.2024.1385064

**Published:** 2024-11-07

**Authors:** Naouras Bouajila, Cloé Domenighetti, Henri-Jean Aubin, Mickael Naassila

**Affiliations:** ^1^ Inserm Unit UMRS 1247, University of Picardie Jules Verne, Amiens, France; ^2^ UVSQ, Univ. Paris-Sud, Inserm, Team “Exposome, Heredity, Cancer, and Health”, CESP, University of Paris-Saclay, Villejuif, France; ^3^ Department of Psychiatry and Addictology, Paul-Brousse Hospital, AP-HP, Center for Epidemiology and Population Health Research (CESP), Inserm 1018, University of Paris-Saclay, Villejuif, France

**Keywords:** single nucleotide polymorphisms, genetic epidemiological studies, alcohol use, conventional epigenetic studies, level of alcohol use, health outcomes

## Abstract

**Background:**

The health effects of alcohol consumption, particularly regarding potential protective benefits of light to moderate intake compared to abstinence, remain a subject of ongoing debate. However, epidemiological studies face limitations due to imprecise exposure measurements and the potential for bias through residual confounding and reverse causation. To address these limitations, we conducted a systematic review of Mendelian Randomization (MR) studies examining the causal relationship between alcohol consumption and cancers, cardiovascular, liver, and neurological diseases.

**Methodology:**

We searched PubMed, ScienceDirect and Embase and Europe PMC up to 05/2024 for MR studies investigating the association of genetically predicted alcohol consumption with cancers, cardiovascular, liver and neurological diseases. We assessed methodological quality based on key elements of the MR design a genetic association studies tool.

**Results:**

We included 70 MR studies that matched our inclusion criteria. Our review showed a significant association of alcohol consumption with multiple cancers such as oral and oropharyngeal, esophageal, colorectal cancers, hepatocellular carcinoma and cutaneous melanoma. While the available studies did not consistently confirm the adverse or protective effects of alcohol on other cancers, such as lung cancer, as suggested by observational studies. Additionally, MR studies confirmed a likely causal effect of alcohol on the risk of hypertension, atrial fibrillation, myocardial infraction and vessels disease. However, there was no evidence to support the protective effects of light to moderate alcohol consumption on cognitive function, Alzheimer's disease, and amyotrophic lateral sclerosis, as reported in observational studies while our review revealed an increased risk of epilepsy and multiple sclerosis. The available studies provided limited results on the link between alcohol consumption and liver disease.

**Conclusions:**

Despite the valuable insights into the causal relationship between alcohol consumption and various health outcomes that MR studies provided, it is worth noting that the inconsistent ability of genetic instrumental variables to distinguish between abstainers, light and moderate drinkers makes it difficult to differentiate between U or J-shaped vs. linear relationships between exposure and outcome. Additional research is necessary to establish formal quality assessment tools for MR studies and to conduct more studies in diverse populations, including non-European ancestries.

**Systematic Review Registration:**

www.crd.york.ac.uk/prospero/display_record.php?ID=CRD42021246154, Identifier: PROSPERO (CRD42021246154).

## 1 Introduction

Alcohol consumption is linked to numerous health impairments, chronic diseases and deaths worldwide (1). In particular, excessive consumption affects the brain, liver, cardiovascular system, and can lead to various cancers (2). Recent studies show significant increases in mortality and disability-adjusted life years associated with alcohol consumption, leading to growing concern worldwide (3). The Global Burden of Disease Study 2021 has shown that alcohol use disorders (AUD) are among the top 25 leading Level 3 causes of global years lived with disability (YLDs) worldwide (4). Some countries have revised their alcohol consumption guidelines, and a recent study suggests the need to lower current thresholds for safer alcohol use as there may be no safe level of alcohol consumption (5, 6).

Alcohol has been classified as a carcinogen by the World Health Organization (7). In 2020, it was estimated that 4.1% of all new cancer cases globally were attributable to alcohol consumption (8). The relationship between alcohol consumption and cancer risk has been extensively studied in traditional epidemiological studies, mostly suggesting that alcohol consumption is causally associated with cancers of the oral cavity, pharynx, larynx, esophagus, colorectum, liver, and female breast in a dose-dependent manner (7, 9). However, J-shaped relationships between alcohol consumption and certain health outcomes suggest a potential protective effect of light or moderate consumption (3, 10, 11), leading to ongoing debate regarding methodological issues impeding causal inference (12–14). These associations are consistent across different types of alcoholic beverages, are monotonic and do not have a threshold (9, 15). The alcohol-attributable fraction is high for upper aero-digestive tract (25%–44%), liver (18%–33%), and colorectal (4%–17%) cancers, and for women breast cancer (about 5%), with variation across European countries depending on levels of alcohol exposure (9, 16, 17). Regular heavy drinking is strongly associated with cancer risk, and reducing alcohol consumption is believed to have a beneficial effect on reducing cancer risk (9). In contrast, alcohol consumption has been negatively associated with the risk of lung cancer (18), kidney cancer (19), and non-Hodgkin lymphoma (20).

The relationship between alcohol consumption and cardiovascular disease (CVD) is complex, and numerous studies have shown both risks and benefits of alcohol consumption for specific diseases. About 10% of deaths related to CVD can be attributed to alcohol consumption (21). Heavy alcohol consumption increases the likelihood of coronary heart disease and cardiovascular mortality (22). Observational studies have consistently shown that alcohol consumption is associated with an increased risk of hemorrhagic stroke, heart failure, and atrial fibrillation, while low levels of alcohol consumption have been associated with a lower risk of coronary heart disease and ischemic stroke (3, 23–26). The protective effect of light to moderate alcohol consumption has been questioned by some authors who suggest that methodological biases may have influenced previous studies. They claim that any potential benefits from light to moderate drinking would be very small and unlikely to outweigh the harms (27, 28).

Alcohol-related liver disease (ARLD) is the primary cause of liver-related mortality and the leading indication for liver transplant in Europe, where liver cirrhosis is a major public health problem, particularly among males. In 2002, it accounted for 1.8% of all deaths in the region, causing over 170,000 deaths (29, 30). ARLD encompasses a range of clinical and histological conditions, including alcoholic fatty liver disease, alcoholic steatohepatitis, cirrhosis, and Hepatitis associated with alcohol (30, 31). The risk of ARLD is increased by harmful alcohol use (>2 drinks per day for women and >3 per day for men) (30). The risk of cirrhosis is higher in women than in men for a given amount of alcohol consumed, and there is a dose-response relationship between the amount of alcohol consumed and the risk of cirrhosis (30). Alcohol consumption is also associated with accelerated fibrosis progression in patients with other types of liver diseases. In the general population, about 40% of cases of advanced non-viral liver disease occur in individuals with metabolic risk factors and regular alcohol intake (32). Alcohol-associated cirrhosis accounted for approximately 27% of 1.32 million deaths related to cirrhosis worldwide in 2017 (30).

Despite reports suggesting conflicting evidence on whether moderate alcohol consumption is protective or detrimental for the development of MASLD (Metabolic Dysfunction-Associated Steatotic Liver Disease) (31, 33, 34), recent reviews have concluded that a protective effect remains unsubstantiated, and consequently, any level of alcohol intake in individuals with MASLD may be harmful to liver health (35, 36).

Alcohol consumption has been positively associated with the onset of all types of dementia – especially early-onset dementia – and cognitive decline (37). However, some patterns of drinking have been linked to beneficial effects (38). Thus, the relationships between alcohol use and cognitive health, including dementia are complex, with potential beneficial effects of light to moderate drinking and detrimental effects of heavier drinking. Methodological issues in underlying studies, such as inconsistent measurement of alcohol use and dementia and insufficient control of potential confounders have been suggested as potential biases (37). A recent review concluded that although causality could not be established, light to moderate alcohol consumption in middle to late adulthood was associated with a decreased risk of cognitive impairment and dementia. In contrast, heavy alcohol drinking was associated with changes in brain structures, cognitive impairments, and an increased risk of all types of dementia (39).

Alcohol consumption has varied effects on health, and it is important to clarify the risks and benefits at the population level (40). Observational studies on alcohol consumption and health-related outcomes have produced conflicting results, and making causal inferences based on these studies can be challenging due to various biases and limitations, including reverse causality, confounding factors, and measurement errors (41). While randomized control trials are the gold standard for inferring causality, they may not be practical or ethical in cases of long latency of exposure to disease onset (42).

To overcome these limitations, Mendelian Randomization (MR) analysis has been proposed as an alternative method. MR studies use genetic variants, mostly single nucleotide polymorphisms (SNPs), associated with an exposure as instrumental variables (IV) to estimate its causal association with an outcome, under certain assumptions that minimize bias from confounding or reverse causation (43–45).

Given the contradictory results of previous studies on the risks and potential benefits of low levels of alcohol intake, some of which may be biased, we conducted a systematic review of the latest research using the MR approach to better understand the effects of alcohol on health outcomes. MR is a powerful tool for examining causality, and we conducted a systematic review of MR studies to examine the causal relationships between alcohol consumption and the main causes of death and morbidity, namely cancers, cardiovascular, neurological, and liver diseases.

The question we addressed in our systematic review of Mendelian Randomization studies was whether there is a causal relationship between genetically predicted alcohol consumption and cancers, cardiovascular, liver and neurological diseases.

## 2 Material and methods

We have conducted a systematic review following the PRISMA (Preferred Reporting Items for Systematic Reviews and Meta-Analysis) guidelines 2020 (46). In accordance with the guidelines, our protocol was registered on the International Prospective Registry of Systematic Reviews (PROSPERO) – CRD42021246154.

### 2.1 Eligibility criteria

Studies have been selected according to the criteria outlined below taking up of the PICOS method. Acronym stands for: Population, Intervention which is in our review defined as the exposure, Comparators, Outcome and Study design.

#### 2.1.1 Population

Studies including men and women without restriction of age or ethnicity, healthy or affected by cancers, cardiovascular, liver or neurological diseases.

#### 2.1.2 Exposure

Studies where the exposure was alcohol consumption, indexed by an instrumental variable (IV) or alcohol indexed by IV is one the exposures, representing any amount of alcohol intake.

#### 2.1.3 Comparators

Studies with any comparative measure of alcohol consumption.

#### 2.1.4 Outcomes

Studies in which the outcome was (i) cancers, cardiovascular, liver or neurological diseases, (ii) an epidemiological indicator or (iii) a risk factor of these diseases that are thought to be intermediates on the path to the diseases or leads to the diagnosis of the disease.

#### 2.1.5 Study design

Mendelian randomization design studies on the association between alcohol intake and cardiovascular, neurological, liver diseases and cancers.

#### 2.1.6 Language

Articles reported in English and French languages.

#### 2.1.7 Publication date

Articles published between January 2000 and May 2024.

#### 2.1.8 Publication type

Only original articles included. Case reports, narrative reviews, letters, editorials, opinions and Conference abstracts were excluded.

#### 2.1.9 Ethics

Studies accepted by an ethics committee.

### 2.2 Information sources

Four electronic bibliographic databases were used for the systematic search: PubMed (Medline), Science direct, Embase and Europe PMC. To ensure literature saturation, we searched the reference lists of included studies and relevant journals identified during the search.

### 2.3 Search strategy

The key words used for the bibliographic search were “Alcohol - Mendelian randomization – Cancer - Cardiovascular disease - Liver disease - Neurological disease-”. They were defined by two examiners (NM and BN). The search was carried out taking into account two methods: a search with “MeSH” terms and a search with “free text” terms in order to correctly translate the concepts and synonyms into English. A separate search was carried out for each type of disease. The keywords were combined with the Boolean operators “AND, OR” to form the search equations represented in the Supplementary Table S1 that we introduced in the “PubMed”, “Science Direct”, “Embase” and “Europe PMC”.

### 2.4 Selection process

The articles were entered in the RAYYAN QCRI software (47), we then proceeded to count and remove the duplicates. Titles and abstracts were independently reviewed by two reviewers (NM and BN) respecting the inclusion and exclusion criteria. Full-text studies were also independently reviewed by the same two reviewers. Disagreements were resolved through discussion with the participation of a third reviewer (AHJ).

### 2.5 Data collection process

Using Microsoft Excel®, a standard data collection table was used to extract data from each study. Data was extracted by one investigator (BN) and verified by another investigator (NM). Disagreements were resolved through discussion and the participation of a third investigator (AHJ). When additional information was needed, the study authors were contacted.

### 2.6 Items

The following items were taken from each included study: Reference, Name of the first author, date of publication, Sample size, Ancestry, Sex, Age, Instrumental variable associated to alcohol, Exposure dataset, outcome, Outcome dataset and results.

### 2.7 Study risk of bias assessment

**Figure 1:**
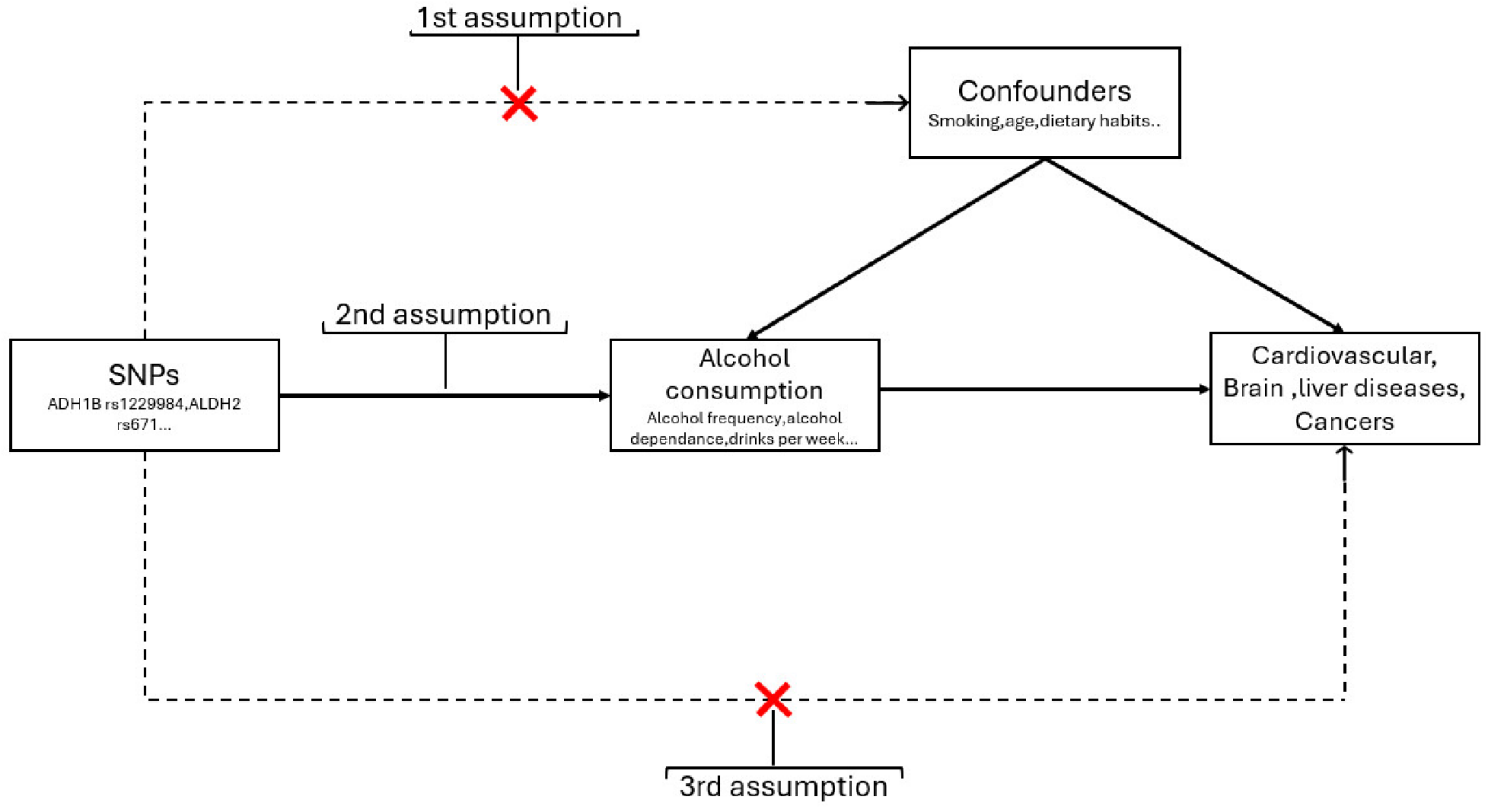
Overview of MR assumptions.

The quality of the studies was independently assessed by three investigators (NM, BN and CD) by using first the Q-Genie tool “Quality of Genetic Association Studies” which contains 11 items rated on a Likert scale. Seven points covered the following topics: rational for the study, selection and definition of the outcome of interest, selection and comparability of comparison groups, technical classification of exposure, non-technical classification of exposure, other sources of bias, sample size and power, *a priori* planning of analyzes, statistical methods and control of confounding factors (48). Second, since the Q-genie tool is not quality assessment tool for MR studies and there was no formal predefined protocol for systematic review of MR to date, we therefore rated other key elements of the MR design including the assumptions that are crucial to the validity of MR as showed in Figure 1:

- *Validation of IV1* – Genetic variants are strongly associated with the exposure: Relevance assumption

The validation of the first assumption in MR studies is evaluated by the selection of strongly associated SNPs with the exposure of interest (*P-values* of the variant-exposure association <5 × 10^−8^). Moreover, to test the robustness of the instrument, the *F-statistic* of the variant-exposure association is usually provided. Values >10 suggest an adequate instrument with a low risk of bias due to a weak instrument (49). The *F-statistic* depends on the proportion of the variance of the exposure explained by the genetic variants (*r*^2^) (50), the sample size and the number of IVs included in the instrument.

- *Validation of IV2* – Genetic variants are not related to confounders of the exposure-outcome association: Independence assumption

Taking into account the random allocation of genetic variants at conception, the IV2 of no association between the IVs and confounders is often fulfilled because of the random allocation of alleles to gametes. In a one-sample MR, in which the variant-exposure and variant-outcome association are computed using individual data from the same sample, the association between the genetic variant and the observed confounders should be tested. However, in a two-sample MR, the variant-exposure and variant-outcome association come from two independent samples, most often using published summary statistics of large genome wide association studies (GWAS), which does not allow to test this hypothesis.

- *Validation of IV3* – Genetic variant does not affect the outcome except through the exposure: Exclusion restriction assumption

Horizontal pleiotropy (i.e., a genetic variant affects other traits which influence the outcome independently of the exposure) may lead to biased MR results because the effect of the variant on the outcome is not exclusively due to the exposure (51). Through assessment of horizontal pleiotropy, the third assumption can be partially verified (52). In addition to the main methods used to estimate the causal MR effect—the two-stage least-squares (2SLS) and the inverse-variance weighted (IVW) methods in a one and two-sample design, respectively (53)—several MR methods have been developed to detect and correct for the bias due to pleiotropy, such as the weighted median (54), weighted mode (55), MR-PRESSO (56) and MR-Egger (57) These methods, originally created for a two-sample MR design, can also be used on large one-sample MR, except for the MR-Egger method which is not recommended for one-sample MR unless the correlation between the variant-exposure and variant-outcome estimates due to confounding can be kept low, or the variability in instrument strength is very high (58).

- Assessment of non-linearity

In order to clarify the causal relationships, it is valuable to identify and characterize non-linear effects when they are present. Non-linear association may result in opposite effects depending on the level of exposure. Such opposing effects have been observed particularly in many observational studies examining the relationship between alcohol consumption and cardiovascular events (59).

These assumptions were rated as “Good” if the assumption was assessed using above-mentioned approaches, “moderate” if the assumption was only described, and “poor” if the assumption was not checked or described. This evaluation of the validity of each assumption using this protocol was assessed independently by three reviewers; any inconsistencies were resolved by discussion. The quality assessment data are presented in Supplementary Tables S2–S9.

### 2.8 Data synthesis

Bibliometric analyzes are represented as a flow diagram that describes the complete process of searching and selecting articles. The summary of the results is presented in tables grouping together the data detailed in supplementary information (Supplementary Tables S10–S13). We synthesized narrative genetic evidence on causal associations between alcohol consumption and cancers, cardiovascular, liver and neurological diseases. The main findings have been classified by outcome.

## 3 Results

### 3.1 Flowchart of studies involved in the MR review

**Figure 2:**
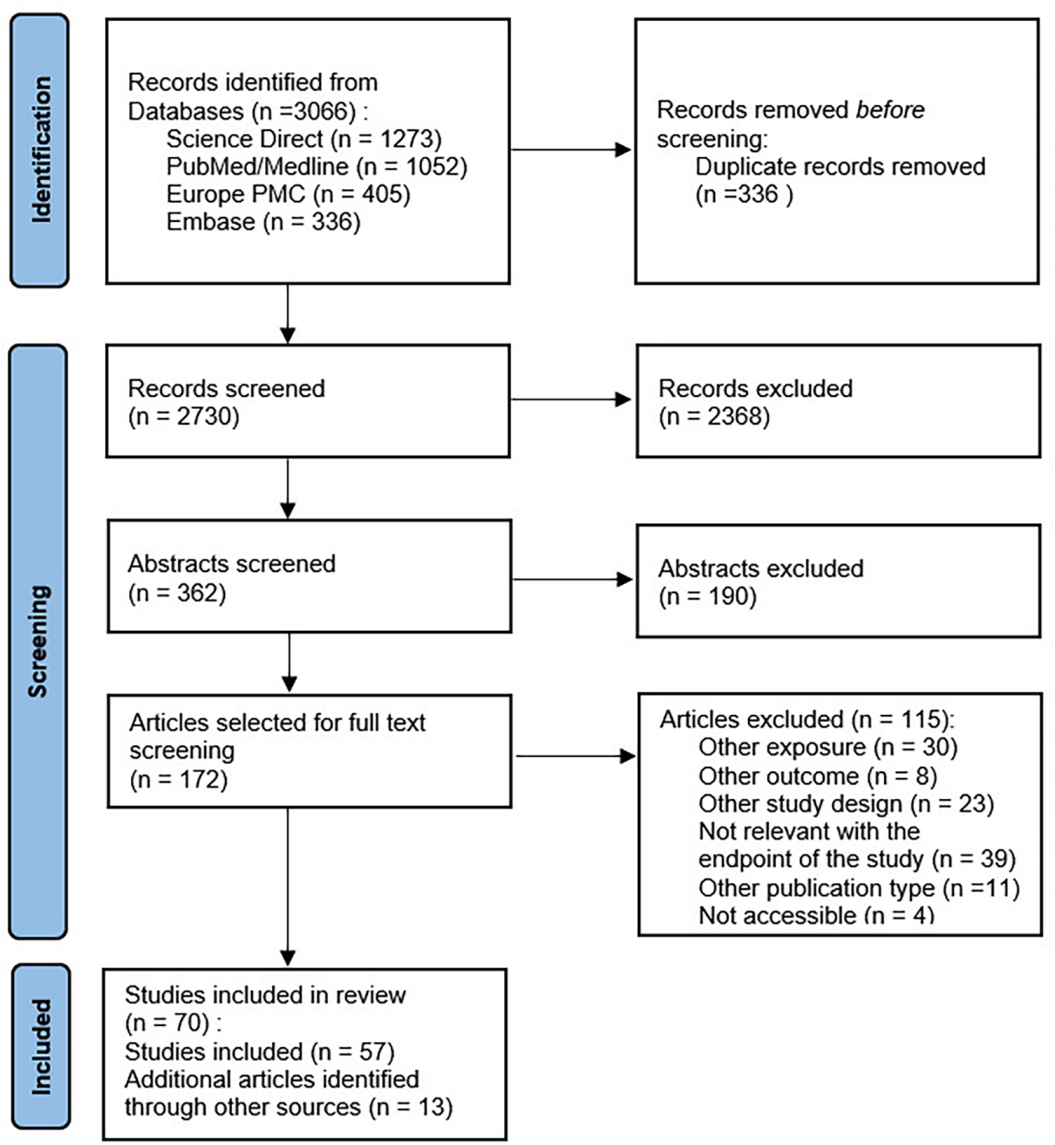
Flow chart of the systematic review 2000–2024.

A flowchart summarizing the study selection process has been produced in accordance with PRISMA guidelines (Figure 2). We identified 3,066 articles from electronic database searches. After removing the duplicates (336 articles), we excluded 2,368 after reading the title and among them, 190 were excluded after reading the abstracts and 110 were excluded among the full text articles. We reviewed the full texts of 172 articles, 57 of which met our inclusion criteria. We identified 13 additional studies via backward reference searches of the 57 included studies, such that a total of 70 studies were eligible for inclusion in our systematic review. The reasons of exclusion are presented in the flow chart.

The main characteristics of the included studies are presented in the tables available in Supplementary Materials. These characteristics have focused on a wide range of outcomes: Cancer outcomes (26 studies), cardiovascular outcomes (24 studies), liver outcomes (6 studies) and neurological outcomes (17 studies). While the majority of the studies were based on populations of European ancestry (47 studies), 12 were Asian and 7 were mixed-ancestry. The majority of studies involved middle-aged and/or older participants.

As for IVs, the studies used either a single genetic variant (12 studies), a combination of genetic variants (51 studies), or a combination of both (8 studies). The variants rs1229984 and rs671, respectively of the genes encoding *ADH1B* and *ALDH2*, are the genetic variants of alcohol consumption most mentioned in these studies. The rs1229984 variant of *ADHB* was used as an IV of alcohol consumption in 12 studies, as was the rs671 variant of *ALDH2* which was used in 7 studies.

### 3.2 Methodological quality assessment

Using the Q-Genie tool, most of the included studies were rated as good overall quality, while 12 studies were rated as moderate quality. Detailed tables of the quality assessment are available in supplementary materials (Supplementary Tables S2–S5).

The MR methodological quality of the included studies was assessed using our protocol, and the results are presented in Supplementary Tables S6–S9.

### 3.3 Synthesis of the results of the studies

All results are synthesized in Supplementary Tables S2–S13. All confidence intervals were estimated at 95% (95% CI) and *p*-value were two-sided in the included studies.

#### 3.3.1 Cancer outcomes

##### 3.3.1.1 All cancers

Larsson et al. (60) performed a two-sample MR analysis and looked for the effects of alcohol consumption and smoking on the risk of a large set of cancers, including (i) consortia of lung, breast, ovarian and prostate cancers, and (ii) the UK-Biobank study notably for site-specific cancer, in European descents. The IVs come from the largest GWAS for alcohol drinking to date, including 941,280 individuals of the GWAS & Sequencing Consortium of Alcohol and Nicotine use (GSCAN) (61). Using 29 SNPs, genetically predicted alcohol consumption was not significantly associated to overall cancer (*n* = 75,037 cases; OR = 0.95, *p* = 0.38) or any other site-specific cancer, except with lung cancer in the International Lung Cancer Consortium (ILCCO) [OR = 1.94 (1.41–2.68), *p* = 4.68×10^−5^]. This study validated all three key MR assumptions, and had a good Q-genie score.

##### 3.3.1.2 Breast, ovarian and endometrial cancers

Seven studies have examined the genetic evidence for the causal association of breast, ovarian and endometrial cancer with alcohol consumption (60, 62–67).

A two-sample MR study (63) assessed the causality of three alcohol-related exposures: number of drinks per week, alcohol use disorder (AUD), and the AUD identification test-concise score (AUDIT-C) adjusted for age (99, 9 and 13 SNPs, respectively). Importantly, the two latter genetically predicted alcohol-related exposures were identified in males only, in a European population from the Million Veteran Program. This study found no evidence of a causal association between alcohol consumption and breast cancer risk (OR_drinks/week_ = 1.01, *p* = 0.89; OR_AUD_ = 1.04, *p* = 0.62; OR_AUDIT−C_ = 1.07, *p* = 0.44) in the Breast Cancer Association Consortium (BCAC; 122,977 cases and 105,974 controls), but as indicated by the authors, the study was underpowered to detect such a relatively modest association as shown in other studies (8% to 12% increase in risk per 10 g/day increase of alcohol consumption).For ovarian cancer, alcohol consumption has been associated with a reduced risk, with a significant association found for AUDIT-C [OR_AUDIT−C_ = 0.83 (0.71–0.97), *p* = 0.02] and a non-significant association found for AUD [OR_AUD_ = 0.92 (0.83–1.01), *p* = 0.08], and for the number of drinks per week (OR_drinks/week_ = 0.83, *p* = 0.19), using data from the Ovarian Cancer Association Consortium (OCAC; 22,406 cases and 40,941 controls). However, the effect was lost by excluding genetic variants associated with potential confounding factors (OR_AUDIT−C_ = 0.89 [0.68–1.16], *p* = 0.38; OR_AUD_ = 0.96 [0.78–1.18], *p* = 0.68).

Furthermore, while the third study suggested that moderate alcohol consumption is associated with a modest increase in breast cancer risk in their observational analysis, no association was found for breast and epithelial ovarian cancer in the BCAC and OCAC using 34 SNPs from white British participants of the UK-Biobank study (62).

Similarly, Zhou et al. (66) in a two sample MR analysis assessed the causality of three alcohol-related exposures: number of drinks per week, alcohol use disorder (AUD), and the Problematic alcohol use (PAU) (84, 19 and 26 SNPs, respectively) found no causal association in the main analysis (OR_drinks/week_ = 1.01, *p* = 0.883; OR_AUD_ = 1.05, *p* = 0.721); OR_PAU_ = 1.03, *p* = 0.781). However, causal effect was observed between PAU and breast cancer incidence risk when conditioning on alcohol consumption [excluding the overlapping or highly correlated genetic IVs (*r*^2^ > 0.1) with drinks per week] (OR_PAU_ = 1.76, *p* = 0.036).

Another two sample MR study using 169 SNPs to instrument drinks per week and included 122,977 cases and 123,082 controls from BCAC, revealed no causal association between alcohol consumption and breast cancer risk (OR_drinks/week_ = 1.01, *p* = 0.829) (65). Additionally, Liu et al. (64) in a two-sample MR study, found no significant association between alcohol consumption, instrumented by 37 SNPs, and the overall risk of ovarian cancer (OR = 0.74, *p* = 0.081), using data from the Ovarian Cancer Association Consortium (OCAC) with 25,509 cases and 40,941 controls.

For endometrial cancer, the MR analysis showed that an increase of one standard deviation in genetically predicted log-transformed alcoholic drinks per day was associated with a 43% reduction in endometrial cancer risk (OR_drinks/week_ = 0.57, *p* < 0.001) in a population comprising 12,906 cases and 108,979 controls. In the subgroup analysis, alcohol consumption was associated with a decreased risk of endometrioid endometrial cancer (EEC) (OR = 0.56, *p* = 0.004), but was not associated with non-endometrioid endometrial cancer (NEC) (OR = 1.36, *p* = 0.626) (67).

These seven studies validated all three key MR assumptions, and had a good/moderate Q-genie score.

##### 3.3.1.3 Prostate cancer

No association was found between genetically-predicted alcohol consumption and prostate cancer in Larsson et al. (60) [OR = 0.96 (0.74–1.24), *p* = 0.75]. Similarly, Brunner et al. (68) in a one sample design found no evidence of a genetic association between ADH/ALDH variants related to alcohol consumption and the incidence of prostate cancer. However, ALDH1B rs10973794 was associated with increased mortality in low-grade prostate cancer [HR = 1.43 (1.14–1.79), *p* = 0.002].

These two studies validated all three MR assumptions and demonstrated high quality as assessed by the Q-Genie tool.

##### 3.3.1.4 Oral and oropharyngeal cancers

While Larsson et al. (60) reported a positive but not significant association of genetically predicted alcohol consumption with head and neck cancer using 6,034 oral/oropharyngeal cases and 6,585 controls from a recent GWAS and 60 SNPs from the GSCAN [OR = 1.75 (0.93–3.72), *p* = 0.14], a two-sample MR study showed a strong evidence of a positive association with oral and oropharyngeal cancers in a population of mixed ancestry using data on GSCAN consortium [OR per 1-SD increase in drinks per week = 10.0 (5.3–18.6), *p* = 5.64 × 10^−13^, 1-SD = 9 additional drinks/week] (69). The association remained significant after controlling for lifetime smoking index using multivariable MR analysis [OR = 5.2 (3.2–8.6)]. The stratified analyses by cancer subsite (oral cavity and oropharyngeal cancer) showed consistent results. Another two sample MR study conducted on European population showed that genetically predicted alcoholic drinks per week instrumented by 34 SNPs was significantly associated with the higher risk of head and neck cancer [OR _drinks/week_ = 1.003 (1.001–1.006); *p* = 0.014]. Conversely, the study found no statistically significant association between genetic predisposition to alcohol consumption instrumented by 7 SNPs and the risk of head and neck cancer [OR = 1.000 (0.999–1.002); *p* = 0.537] (70).

Im et al. (71) in a two sample MR design using ALDH2-rs671 and ADH1B-rs1229984 as IVs in an Asian population found no significant causal relationship between genetically predicted alcohol consumption and the risk of lip, oral cavity, and pharynx cancer in both men and women. The hazard ratios for an increase of 280 g/week in alcohol consumption were 1.02 [0.59–1.75] for men and 0.95 [0.45–1.99] for women. In addition, this study found no significant causal relationship between genetically predicted alcohol consumption and the risk of larynx cancer in both men and women. The hazard ratios per 280 g/week of alcohol consumption were 0.58 [0.14–2.34] for men and 0.02 [0.00–4.45] for women.

These four studies validated all three key MR assumptions, and had a good Q-genie score.

##### 3.3.1.5 Lung cancer

As mentioned above, Larsson et al. (60) reported a strong positive association of genetically predicted alcohol consumption with lung cancer in the ILCCO [OR = 1.94 (1.41–2.68), *p* = 4.68 × 10^−5^] but not in UK Biobank [OR = 1.12 (0.65–1.93), *p* = 0.69]. However, this finding contradicts a two-sample MR performed by Chen et al. (72) that aimed to determine the relation between habitual alcohol consumption with meals (described as appropriate, light-to moderate, less than 30 g/day) and lung cancer. Using 14 SNPs provided by the UK-Biobank study, significant inverse associations were found with lung cancer [OR = 0.175 (0.045–0.682), *p* = 0.012] and lung squamous cell cancer [OR = 0.075 (0.013–0.429), *p* = 0.004] but not with lung adenocarcinoma (OR = 1.00, *p* = 0.90) using data from ILCCO, and an inverse but not significant association was found with small cell lung cancer [OR = 0.25 (0.052–1.169), *p* = 0.078] using data from the UK-Biobank study.

Another two sample study on Asian population using ALDH2-rs671 and ADH1B-rs1229984, an increase of 280 g/week in alcohol consumption was associated with a decreased risk of lung cancer in men [HR = 0.81 (0.67–0.98)]. For women, a similar trend was observed, but it was not statistically significant [HR = 0.85 (0.68–1.06)] (71).

Ding et al. (73) in a two sample design using ADH1B rs1229984 as IV in 2,485 lung cancer cases and 410,350 controls of European ancestry showed no causal relationship between habitual alcohol intake and lung cancer [OR = 1.30 (0.39–4.35), *p* = 0.674], authors suggest that this may be due to the use of only 1 genetic variant, resulting in insufficient statistical power.

These studies validated all three key MR assumptions and achieved good Q-Genie scores, except for the study by Ding et al. (73), which validated only two of the assumptions.

##### 3.3.1.6 Esophageal cancer

Six studies investigated the association between genetically predicted alcohol consumption and esophageal cancer.

Larsson et al. (60) using 843 cases from the UK Biobank, reported strong but statistically non-significant positive associations of genetically predicted alcohol consumption with esophageal cancer [OR = 1.88 (0.76–4.66); *p* = 0.171]. Conversely, Im et al. (71) found a significant positive association between genetically predicted consumption of 280 grams per week and an increased risk of esophageal cancer in men [HR = 1.47 (1.09–1.99)]. In women, although the hazard ratio suggested a 58% increased risk, the association was not statistically significant [HR = 1.58 (0.85–2.93)].

Yuan et al. (74) in a two-sample design using 1,130 cases and 702,116 controls from a European population, reported a significant positive association with esophageal cancer [OR = 2.86 (1.18–6.91), *p* = 0.020]. However, this association became non-significant in multivariable Mendelian randomization (MVMR) analysis when adjusted for smoking, suggesting a possible synergistic effect between alcohol and smoking [adjusted OR = 1.28 (0.59–2.82), *p* = 0.533]. Zou et al. (75), in a two-sample design using data from the FinnGen consortium (503 cases; 259,583 controls), showed no statistically significant association between alcohol intake frequency and esophageal cancer risk [OR = 0.206 (0.545–2.668), *p* = 0.644]. Inversely, Using data from Biobank Japan (1,300 cases and 195,745 controls) performed on asian population ancestry, Cai et al. (76) reported a strong significant positive association between ever/never drinking and esophageal cancer risk [OR_ever/never drinker_ = 2.24E4 (40.02–1.25E7), *p* = 0.0019]. Zhang et al. (77) in a two sample analysis reported no causal effect between alcohol and esophageal squamous cell carcinoma (ESCC) risk [OR = 0.99 (0.99–1.00), *p* = 0.71] using 5 SNPs. However, the polymorphism ADH1B rs1229984 was associated with an increased risk of ESCC [OR = 2.50 (1.70–3.69)]. Inversely, the ALDH2 rs671 variant was found to be associated with a decreased risk of ESCC [OR = 0.60 (0.50–0.73)]. For the ALDH2 rs674 variant, the association with ESCC showed an OR = 1.22 [0.71–2.12], indicating no significant association. Similarly, the ADH1B rs1042026 [OR = 1.28 (0.52–3.14)] also showed no significant association with ESCC. The study also showed that smokers with the rs1229984 risk allele had a further increased risk of ESCC [OR = 1.39 (1.21–1.59), *p* < 0.001] compared with nonsmokers [OR = 1.52 (1.36–1.70)].

All these studies utilized a two-sample design, validated all three key MR assumptions, and achieved good Q-Genie scores.

##### 3.3.1.7 Gastric cancer

Five studies investigated relationship between genetically predicted alcohol intake and gastric cancer.

Larsson et al. (60) using 736 stomach cancer cases from UKB indicated no statistically significant association between genetically predicted alcohol consumption and the risk of stomach cancer [OR = 0.88 (0.33–2.30); *p* = 0.788]. Similarly, Im et al. (71) reported no statistically significant association between alcohol consumption of 280 grams per week and the risk of stomach cancer for either men [HR = 1.16 (0.89–1.50)] or women [HR = 0.85 (0.61–1.20)]. In addition, Yuan et al. (74) in a two-sample design with 1,608 cases and 701,472 controls, conducted a univariable Mendelian randomization analysis that suggested a potential, though non-significant, increase in the risk of gastric cancer associated with genetically predicted alcohol consumption [OR = 1.57 (0.75–3.30); *p* = 0.233]. In the multivariable Mendelian randomization (MVMR) analysis, adjusted for smoking, the association remained consistent, indicating a positive but non-significant relationship [OR = 1.59 (0.79–3.21); *p* = 0.194]. Another MR study reported a statistically negative association between genetically predicted ever drinker/never drinker and a reduced risk of gastric cancer, with a 5% lower odds compared to never drinkers [OR_ever/never drinker_ = 0.95 (0.93–0.98)]. However, after excluding rs671, the causal associations between alcohol consumption and gastric cancer were no longer observed (76). Finally, Tan et al. (78), in a two-sample design using data from GWAS datasets from the MRC Integrative Epidemiology Unit (6,563 gastric cancer cases; 195,745 controls), showed no causal relationship between alcohol consumption and gastric cancer. The analysis in a European population, using 179 SNPs related to alcohol, showed no significant association [OR = 1.05 (0.94–1.18), *p* = 0.359]. Similarly, in an asian population, using 66 SNPs, there was also no significant association [OR = 1.01 (0.98–1.04), *p* = 0.562].

All these studies utilized a two-sample design, validated all three key MR assumptions, and achieved good Q-Genie scores.

##### 3.3.1.8 Colorectal cancer

Seven studies investigated the relationship between genetically predicted alcohol consumption and colorectal cancer.

Im et al. (71) indicated an increased risk of colon cancer with higher consumption of alcohol per 280 grams per week in men. Nevertheless, this association is not statistically significant [HR = 1.38 (0.90–2.11)]. Similar results were reported for women [HR = 1.23 (0.89–1.72)]. As for rectal cancer, the study indicated a slight increase in the risk of rectal cancer with higher consumption of alcohol per 280 grams per week. However, this association is not statistically significant [HR = 1.01 (0.71–1.46)]. Inversely, for women, results indicated a reduced risk. This association is also not statistically significant [HR = 0.79 (0.55–1.14)]. Findings from another study (60) using 5,486 cases from UK Biobank indicated a positive association of alcohol consumption with colorectal cancer risk. However, the association is not statistically significant [OR = 1.31 (0.84–2.04); *p* = 0.235]. Zhou et al. (79) in a two sample MR analysis using data summary-level data from 12 colorectal GWASs (20,049 cases; 22,661 controls) performed on European population,reported a statistically significant association between genetically predicted number of drinks consumed per week and the risk of colorectal cancer [OR_drinks/week_ = 1.79 (1.23–2.61), *p* = 0.003]. The association between alcohol use disorder and colorectal cancer risk was also positive although not statistically significant [OR_AUD_ = 1.33 (0.95–1.85), *p* = 0.093]. As for problematic alcohol use, a significant positive association was observed [OR_PAU_ = 1.53 (1.02–2.29), *p* = 0.040]. Yuan et al. (74) conducted a study using 9,519 colorectal cancer cases and 686,953 controls. Both univariable and multivariable analyses adjusted for smoking were performed. The study reported a positive but non-significant association in both analyses, [OR _UV_ = 1.09 (0.76–1.55); *p* = 0.649] for the univariable analysis [OR_MVMR_ = 1.28 (0.95–1.72); *p* = 0.098] and for the multivariable Mendelian randomization analysis. He et al. (80) in a two sample MR study found no statistically significant associations between various measures of alcohol consumption and the risk of colorectal cancer: current alcohol drinkers [OR = 1.012 (0.974–1.051); *p* = 0.556], Never Alcohol Drinkers [OR = 1.010 (0.957–1.067); *p* = 0.715], previous alcohol drinkers [OR = 1.001 (0.935–1.072); *p* = 0.971], alcohol consumption in Females [OR = 1.004 (0.998–1.011); *p* = 0.184], alcohol consumption in males [OR = 1.001 (0.993–1.008); *p* = 0.870], and Alcohol Intake Frequency [OR = 0.999 (0.997–1.001); *p* = 0.154]. Another study using 7,062 colorectal cancer cases and 195,745 controls, reported a negative non-significant association between ever/never drinker and colorectal cancer [OR = 0.84 (0.23–3.07); *p* = 0.7952] (76). Finally, Li et al. (81) in a two sample MR analysis performed on Asian population (6,692 cases; 27,178 controls) investigated the relation between ever vs. never drinkers instrumented by 6 SNPs and number of drinks per week instrumented by 2 SNPs reported that genetically predicted alcohol consumption (ever vs. never drinker) was positively associated with the risk of colorectal cancer [OR_ever/never drinker_ = 1.08 (1.05–1.12), *p* = 1.51 × 10^−5^] and the number of alcoholic drinks per week was also associated with an increased risk of colorectal cancer [OR _drinks/week_ = 1.39 (1.27–1.52), *p* = 5.29 × 10^–13^]. Sensitivity analysis yielded similar results.

These studies validated all three key MR assumptions and achieved good Q-Genie scores.

##### 3.3.1.9 Liver cancers

Three studies explored the association between genetically predicted alcohol intake and hepatocellular carcinoma (HCC), a subtype of liver cancer, while three other studies investigated the association between alcohol intake and liver cancer overall.

Deng et al. (82), in a one-sample MR design using data from Biobank Japan (1,866 cases; 195,745 controls) performed on Asian population, reported a significant positive association between alcohol consumption, instrumented by ADH1B rs1229984 and ALDH2 rs671, and HCC risk [OR = 1.57 (1.32–1.86), *p* < 0.001]. Using 5 SNPs related to ever/never drinker status, the study also found that people who had ever consumed alcohol had a higher HCC risk compared to never drinkers [OR = 1.11 (1.05–1.18), *p* < 0.001]. Cai et al. (76) reported a slight increase in HCC risk among ever drinkers [OR = 1.11 (1.05–1.18)]. Another one-sample MR non-linear analysis performed on a European population found a positive association between pure alcohol intake (g/day) and the risk of HCC, demonstrating a statistically significant linear trend (*p* < 0.0001) (83). Im et al. (71) indicated a positive non-significant association between higher alcohol consumption and liver cancer risk in men [HR per 280 g/week = 1.23 (0.93–1.62)] and a negative non-significant association between higher alcohol consumption and liver cancer risk in women [HR per 280 g/week = 0.85 (0.61–1.20)].

Using 714 liver cancer cases and 702,008 controls, another MR study demonstrated that neither the UVMR nor the MVMR adjusted for smoking analyses showed statistically significant associations between alcohol consumption and liver cancer risk [OR = 1.16 (0.43–3.11); *p* = 0.775] and [OR = 0.76 (0.29–2.02); *p* = 0.585], respectively (74). Finally, Zhang et al. (84) in a two sample MR design reported a 57% increase in liver cancer risk with each standard deviation increase in log-transformed alcoholic drinks per week. However, this association was not statistically significant [OR _drinks/week_ = 1.57 (0.57–5.03); *p* = 0.339].

These six studies validated all three MR assumptions and were reported to be of good quality according to the Q-Genie tool, with the exception of those by Deng et al. (82) and Liu et al. (83) These two studies presented methodological shortcomings, such as a one-sample design and poor validation of the third assumption.

##### 3.3.1.10 Pancreatic cancer

Three MR studies examined the relationship between genetically predicted alcohol consumption and pancreas cancer.

Larsson et al. (60) using 1,264 cases from the UK Biobank, revealed no statistically significant association between alcohol consumption and pancreas cancer risk [OR = 1.16 (0.55–2.43); *p* = 0.703].

In another MR study, the univariable Mendelian randomization (UVMR) analysis showed no statistically significant association [OR = 0.63 (0.32–1.26), *p* = 0.193]. Similarly, the multivariable Mendelian randomization (MVMR) analysis adjusted for smoking also indicated no significant association [OR = 0.79 (0.40–1.56), *p* = 0.496] (74).Cai et al. (76) using data from 442 cases and 195,745 controls retrieved from BBJ, analyzed the association between ever/never drinkers instrumented by 5 SNPs. The results showed no statistically significant association [OR = 1.01 (0.92–1.10), *p* = 0.9119]. However, after excluding rs671, a suggestive causal association between alcohol drinking and pancreatic cancer development was observed.

These studies validated all three MR assumptions and were reported to be of good quality according to the Q-Genie tool.

##### 3.3.1.11 Bladder cancer

Two studies investigated the relationship between genetically predicted alcohol consumption and bladder cancer (60, 85).

Larsson et al. (60), using 2,588 cases, investigated the association between alcohol consumption and the risk of bladder cancer and found no statistically significant association [OR = 0.85 (0.49–1.44), *p* = 0.539]. Similarly, Xiong et al. (85), in a two-sample MR analysis reported that genetically predicted drinks per week were not associated with bladder cancer [OR _drinks/week_ = 0.69 (0.44–1.10), *p* = 0.1237]. These studies validated all three MR assumptions and were reported to be of good quality according to the Q-Genie tool.

##### 3.3.1.12 Cutaneous melanoma

Only one study in our review examined the relationship between genetically predicted alcohol intake and cutaneous melanoma. This two-sample Mendelian randomization study, utilizing data from the FinnGen consortium (2,993 cases; 287,137 controls), reported a statistically significant association between the number of drinks per week and the risk of cutaneous melanoma [OR _drinks/week_ = 2.23 (1.11–4.47); *p* = 0.02] (86). This study demonstrated good methodological quality, as it validated all three MR assumptions and received a high-quality rating in the Q-Genie tool.

##### 3.3.1.13 Thyroid cancer

Wang et al. (70) conducted a study involving 989 thyroid cancer cases, examining the relationship between alcohol consumption and thyroid cancer using 34 SNPs for drinks per week and 7 SNPs for overall alcohol consumption. The study found no statistically significant associations for drinks per week [OR_drinks/week_ = 1.407 (0.461–4.294), *p* = 0.549] and for overall alcohol consumption [OR = 1.188 (0.762–1.850), *p* = 0.447]. This study was of high methodological quality, as it validated all three MR assumptions and received a favorable rating in the Q-Genie tool.

##### 3.3.1.14 Biliary tract cancer

Only one study investigated the relationship between alcohol consumption and biliary tract cancer, using 339 cases and 195,745 controls retrieved from BBJ. The study reported no significant association (OR ever/never drinker = 0.98 [0.89–1.07], *p* = 0.6363 (76). As previously mentioned, this study employed a two-sample design, validated all three MR assumptions, and achieved a high-quality score according to the Q-Genie tool.

#### 3.3.2 Cardiovascular outcomes

Regarding ischemic heart disease, a one-sample MR study using 8,408 cases from using the China Kadoorie Biobank found no association with mean alcohol intake instrumented by ALDH2-rs671 and ADH1B-rs1229984 [HR per 280 g per week = 1.04 (0.94–1.14), *p* = 0.457] (71). This study also showed a U-shaped association with a conventional epidemiological analysis but the apparent protective effect of moderate drinking is not confirmed in the MR analysis. Using the ADLH2-rs671 variant, Au Yeung et al. (87) did not found significant association between alcohol consumption and CVD or ischemic heart disease. These two studies utilized a one-sample design, received good quality scores on the Q-Genie tool, and validated at least two MR assumptions.

Some studies reported no association concerning stroke and vessel diseases. One-sample MR analysis that used the *ADH1B*-rs1229984 and *ADH1C*-rs698 variants found no association with stroke in a Danish population [OR = 1.15 (0.66–2.02)] (88). The two-sample MR study of Jia et al. (89) using 40,585 cases and 406,111 controls did not find a significant association of alcohol consumption or dependence with ischemic stroke. Harshfield et al. (90) used a two-sample MR analysis to explore the risk of all stroke and ischemic stroke subtypes from the MEGASTROKE Consortium using data from 67,162 European cases (60,341 cases with any ischemic stroke regardless of subtype, of which 9,006 were cardioembolic stroke, 6,688 were large artery stroke, and 11,710 were small vessel stroke) and 454,450 controls. They did not report any association between alcohol consumption and any type of stroke. However, other studies reported significant association. The one-sample MR study using 12,176 Asian cases found a statistically significative positive association of genotype-predicted mean alcohol intake with stroke [HR per 280 g per week = 1.38 (1.27–1.49), *p* = 6.8 × 10^−15^] (71). This study also showed a U-shaped association with a conventional epidemiological analysis but the apparent protective effect of moderate drinking is not confirmed in their MR analysis. A one-sample MR study including 261,991 European descents showed that the *ADH1B*-rs1229984 variant is associated with non-drinking and lower alcohol consumption had a reduced risk of ischemic stroke (but not the combined subtypes of stroke) than those without the genetic variant (91). Biddinger et al. (92) in a two-sample MR design using 8,710 cases from UK Biobank found a significative positive association with stroke [OR per 1-SD increase in genetically predicted alcohol consumption = 1.26 (1.04–1.54), *p* = 2.10 × 10^−2^]. Lankester et al. (93) reported that all stroke was positively associated with alcohol in all UK Biobank one-sample analyses, but null in MEGASTROKE [67,162 (all stroke); 60,341 (ischemic stroke)]. While ischemic stroke analyses were all null, hemorrhagic stroke was positively associated with alcohol use in UK Biobank analyses. Hu et al. (94) explored the causal effects of moderate alcohol intake on cardiovascular diseases including stroke in a prospective cohort of 40,386 Chinese males (genotyped for ALDH2-rs671, 2,406 incident CVD). Their one-sample MR analyses revealed a linear association of genetically predicted alcohol consumption with the incident CVD [HR = 1.27 (1.05–1.53), *p* = 0.02], including total stroke [HR = 1.33 (1.02–1.74), *p* = 0.04]. No significant effects were obtained for ischemic stroke, but they found that genetically predicted alcohol consumption was associated with increased risk of hemorrhagic stroke, with a linear trend (*p* = 0.02). In Chinese population, the one-sample MR study from Millwood and colleagues (95) used the *ALDH2*-rs671 and *ADH1B*-rs1229984 variants for alcohol consumption to estimate its relation with cardiovascular outcome (*n* = 161,498). In men, their conventional epidemiology analysis adjusted to smoking showed that self-reported alcohol intake had U-shaped associations with the incidence of ischemic stroke, and intracerebral hemorrhage. In contrast, their MR analysis showed a log-linear rather than a U-shaped association with stroke. No association between alcohol intake and stroke was found in women, a finding related to the very low prevalence of drinking found in Chinese women. A methodologically sound two-sample MR study performed by Larsson et al. (40) assessed the relation between alcohol consumption (94 SNPs from the GSCAN consortium) and eight cardiovascular diseases using a meta-analysis of data of mainly European descents from several consortia and the UK-Biobank study. They showed that genetically predicted alcohol consumption was associated with stroke [OR per 1-SD of log-transformed alcoholic drinks per week = 1.27 (1.12–1.45), *p* = 2.87 × 10^−4^], peripheral artery disease [OR = 3.05 (1.92–4.85), *p* = 2.30 × 10^−6^], coronary artery disease [OR = 1.16 (1.00–1.36), *p* = 0.052] and abdominal aortic aneurysm [OR = 2.60 (1.15–5.89), *p* = 0.022]. In addition, associations with stroke types remained significant (OR for ischemic stroke = 1.26, *p* = 0.002; OR for intracerebral hemorrhage = 3.53, *p* = 0.001). These associations were attenuated in multivariable MR analysis adjusted for smoking initiation, leaving a significant association only for stroke and peripheral artery disease. However, no association was found with venous thromboembolism (*p* = 0.810), and aortic valve stenosis (*p* = 0.926). Hisamatsu et al. (96) reported in their MR study on 682 Japanese men genotyped for ALDH2-rs671 the causal role of alcohol intake in cerebral small- and large-vessel diseases. They found a positive association of alcohol consumption with risk of cerebral small-vessel disease [Age-adjusted OR = 1.46 (1.09–1.94)] and its inverse association with risk of large-vessel disease [Age-adjusted OR = 0.70 (0.50–0.98)]. However, these associations attenuated to statistical non-significance after considering covariates and amount of alcohol intake. In a two-sample MR study, Tian et al. (97) reported on a mixed population that genetically predicted alcohol intake is not associated with the risk of intracranial aneurysms [OR = 1.29 (0.68–2.45), *p* = 0.43]. Larsson et al. (98) conducted MR analyses on the link between alcohol intake and the risk of intracerebral hemorrhage using GWAS data on European participants from three different sources: FinnGen, UK Biobank and previous GWAS by Woo et al. (99). Their univariable MR analysis showed a significant association with alcohol intake [OR = 1.59 (1.07–2.35), *p* = 2.07 × 10^−2^]. The association between genetically predicted alcohol consumption and intracerebral hemorrhage differed across studies, with a significant positive association in the UK Biobank, a suggestive positive association in the Woo et al. GWAS, and no association in FinnGen.

Regarding hypertension, in general, MR studies consistently found a significant association with genetically predicted alcohol consumption. A two-sample MR study (100) based on a pooled analysis from the UK-Biobank (54,358 cases, 408,652 controls) and FinnGenn (15,870 cases, 74,345 controls) cohorts of European descents showed a positive association with alcohol dependence [OR = 1.10 (1.06–1.13), 3 SNPs] and with alcohol consumption [OR = 1.28 (1.07–1.52), 99 SNPs]. In Asian population, Zhao et al. (101) found in a one-sample MR study that genetically predicted alcohol consumption was associated with hypertension in men of Chinese descents (OR = 1.19, *p* = 0.011), but not in women (*p* = 0.317), using the association of rs671 in the *ALDH2* gene with alcohol use in 2,349 participants. Similar results were found in a South Korean one-sample MR including 7,152 individuals using the same genetic variant (102). A two-sample MR study using data from the UK Biobank found a positive association with hypertension [OR per 1-SD increase in genetically predicted alcohol consumption = 1.28 (1.18–1.39), *p* = 1.73 × 10^−9^] (92).

Concerning Coronary artery/heart disease (CAD/CHD), Biddinger et al. (92) using 27,667 cases from European ancestry found a positive association with coronary artery disease [OR per 1-SD increase in genetically predicted alcohol consumption = 1.38 (1.10–1.74), *p* = 6.00 × 10^−3^]. The two-sample MR study of Jia et al. (89) did not find a significant association of alcohol consumption or dependence with coronary artery disease using 60,801 cases and 123,504 controls. Although a non-significant link was observed between alcohol consumption and CAD in the principal analysis, the MR-PRESSO approach revealed a suggestive positive relationship [OR per 1-SD increase = 1.19 (1.00–1.40)]. A two-sample MR study of Yang et al. (103) did not find any association of genetically predicted alcohol measured by drinks per week with the risk of CAD (OR = 1.11 [0.92–1.35). However, this study found a significant association of alcohol dependence with the risk of CAD [OR = 1.04 (1.02–1.06); *p* < 0,001]. Hu et al. (94) explored the causal effects of moderate alcohol intake instrumented by ALDH2-rs671 on cardiovascular diseases including coronary artery diseases using 2,406 incident CVD cases. They observed a J-shaped association of self-reported alcohol consumption with incident CVD, showing decreased risks for light (≤25 g/day) and moderate drinkers (25−≤60 g/day). However, the one-sample MR analyses revealed a linear association of genetically predicted alcohol consumption with the incident CVD [HR = 1.27 (1.05–1.53), *p* = 0.02], including CAD [HR = 1.46 (1.01–2.11), *p* = 0.04]. After excluding heavy drinkers, the risk of incident CVD was increased by 27% per standard drink increment of genetically predicted alcohol consumption. A two-sample MR conducted in European decent by Rosoff et al. (104) that also used SNPs from the GSCAN consortium (*n* = 71) showed that genetically predicted alcohol consumption was positively associated with CHD [OR = 1.21 (1.01–1.45)], but the effect was attenuated to a non-significant level after adjustment for smoking, whereas an association with coronary atherosclerosis remained significantly positive [OR = 1.02 (1.01–1.03), *p* = 5.56 × 10^−4^]. A one-sample MR study including 261,991 European descents showed that the *ADH1B*-rs1229984 variant is associated with non-drinking and lower alcohol consumption had a reduced risk of coronary heart disease than those without the genetic variant (91). However, when analysis was restricted to non-drinkers, the association was null.

Concerning myocardial infarction, Biddinger et al. (92) using 14,503 cases reported a significant positive association with myocardial infarction [OR = 1.37 (1.05–1.78), *p* = 2.00 × 10^−2^]. In contrast, Lankester et al. (93) provided inconsistent results. Their study found a positive association between alcohol consumption and myocardial infarction in the UK Biobank, but this association did not hold in the multivariable Mendelian randomization (MVMR) analysis and was not replicated in the CARDIOGRAMplusC4D dataset. A two-sample MR performed on European decent by Rosoff et al. (104) using SNPs from the GSCAN consortium (*n* = 71) showed that genetically predicted alcohol consumption was positively associated with myocardial infarction [OR = 1.24 (1.03–1.50)]. However, the effect was attenuated to a non-significant level after adjustment for smoking. Millwood et al. (95) in a one sample design used the *ALDH2*-rs671 and *ADH1B*-rs1229984 variants for alcohol consumption to estimate its relation with cardiovascular disease (*n* = 161,498) in Chinese population. In men, their conventional epidemiology analysis adjusted to smoking showed that self-reported alcohol intake had U-shaped associations with the incidence of acute myocardial infarction. However, the genotype-predicted mean alcohol intake was not significantly associated with myocardial infarction (RR per 280 g/week = 0.96, *p* = 0.69). No association between alcohol intake and myocardial infarction was found in women, a finding related to the very low prevalence of drinking found in Chinese women.

Concerning heart failure, a two-sample MR study using 5,812 cases found a positive association with stroke [OR = 1.39 (1.08–1.78), *p* = 9.00 × 10^−3^] (92). Lankester et al. (93) found that Heart failure was positively associated with alcohol use in the initial UK Biobank analysis, but associations were null in external datasets, thus providing inconsistent results. Another two-sample MR (105) also showed no significant association between alcohol consumption (using 91 SNPs also from the GSCAN consortium) and heart failure in 47,309 cases and 930,014 controls of European descents (*p* = 0.30). Similarly, The two-sample MR study performed by Larsson et al. (40) showed that genetically predicted alcohol consumption was not associated with heart failure (*p* = 0.996).

Concerning atrial fibrillation, a two-sample MR study utilizing data from the AFGen consortium on European individuals (17,931 cases and 115,142 controls) focusing on daily alcohol consumption did not find a causal association with incidence of AF [OR = 1.09 (0.72–1.76)] (106). In another two-sample study, Jiang et al. (107) did not find any association using 43 SNPs from the GSCAN consortium for alcohol intake (*p* = 0.979), 12 SNPs for alcohol dependence from the PGC consortium (*p* = 0.26) and 12 SNPs from the UK-Biobank for AUDIT score (*p* = 0.827). However, five other MR studies suggested that the association between alcohol consumption and AF may be causal. A two-sample MR study, using both linear and non-linear approaches, with data from the UK Biobank (92) on 14,367 European cases found a positive association with stroke [OR = 1.24 (1.08–1.44), *p* = 3.00 × 10^−3^]. The positive association with in increased risk of AF is confirmed in another two-sample MR study on mixed ancestry (European, Japanese, African American, Brazilian and Hispanic) showing that heavy alcohol consumption (>35 units/week in women and >50 units/week in men) increased AF risk [OR = 1.11 (1.04–1.18), *p* = 0.001] (108). The two-sample MR study performed by Larsson et al. (37) assessed the relation between alcohol consumption (94 SNPs from the GSCAN consortium) and eight cardiovascular diseases using a meta-analysis of data of mainly European descents from several consortia and the UK Biobank study. They showed that genetically predicted alcohol consumption was associated with atrial fibrillation [OR = 1.17 (1.00–1.37), *p* = 0.050]. Another one-sample MR study using 8,964 Asian participants genotyped for the ALDH2-rs671 SNP found a significant association with AF in men but not in women [OR = 3.00 (1.13–8.68)] and multivariate model [OR = 3.17 (1.18–9.24)] (109), conversely to their observational analysis. Lankester et al. (93) reported that one additional drink of alcohol per day was positively associated with AF [OR = 1.26 (1.07–v1.48)] in their one-sample (UK Biobank) analysis but two-sample MR association was null.

#### 3.3.3 Liver outcomes

Three studies investigated the association between genetically predicted alcohol consumption and NAFLD. Sookoian et al. (110) used the rs1229984 variant of *ADH1B* to investigate the association between genetically predicted alcohol consumption and the histology of MASLD in an European population of 331 MASLD cases and 135 controls. The results suggested that in patients with MASLD at high-risk for progressing to end-stage liver disease, alcohol consumption even at moderate amount might be harmful and thus suggested no beneficial effect of moderate alcohol consumption on MASLD disease severity. Carriers of the A-allele consumed significantly lower amounts of alcohol compared with noncarriers (2.3 ± 5.3 vs. 8.18 ± 21 g per day, mean ± s.d., *p* = 0.03), and showed lower degree of histological steatosis (1.76 ± 0.83 vs. 2.19 ± 0.78, *p* = 0.03), and lower scores of lobular inflammation (0.54 ± 0.65 vs. 0.95 ± 0.92, *p* = 0.02) and MASLD-Activity Score (2.9 ± 1.4 vs. 3.7 ± 1.4, *P* = 0.015) compared with noncarriers. Thus, the group with higher lifetime alcohol consumption (albeit very modest in this study because the inclusion criteria for MASLD impose indeed obvious restrictions into this variable) showed markers of more severe disease on biopsy, even though alcohol consumption was at very modest levels. The study sample comprised of a small number of subjects who drink alcohol in moderate amounts and a small number of events owing to the low frequency of the variant. However, this study reported methodological shortcomings and validated only two assumptions.

Yuan et al. (111) in a two sample MR design used 84 SNPs to investigate the relationship between genetically predicted alcohol consumption and the risk of MASLD. The results indicated a statistically significant inverse association between genetically predicted alcohol consumption and the risk of MASLD [OR = 0.61 (0.38–0.96); *p* = 0.03]. However, the findings need further validation due to moderate-to-high heterogeneity and potential pleiotropy in the genetic instruments used. Another two-MR study design using 3,242 cases and 707,631 controls from European ancestry using a set of 84 SNPs related to alcohol consumption measured by number of drinks per week reported no significant association with the risk of MASLD [OR = 1.20 (0.63–2.28); *p* = 0.574]. In contrast the study reported a strong and statistically significant association between genetically predicted alcohol consumption and a markedly increased risk of developing Alcoholic liver disease [OR = 14.35 (7.69–26.81); *p* = 6.32 × 10^−17^] (74). This study validated all three MR assumptions and was rated as high quality according to the Q-Genie tool.

As for cirrhosis, two studies explored the association with genetically predicted alcohol consumption. Yuan et al. (74) reported a statistically significant association between genetically predicted alcohol consumption and an increased risk of cirrhosis [OR = 2.96 (1.50–5.85); *p* = 0.002]. Similarly, Im et al. (71) using 499 cases demonstrated a strong and statistically significant positive association between alcohol consumption instrumented by ALDH2-rs671 and ADH1B-rs1229984 and the risk of developing cirrhosis [HR = 2.30 (1.58–3.35); *p* = 1.5 × 10^−5^]. As mentioned above these two studies are two sample design, validated all three MR assumptions and was rated as high quality according to the Q-Genie tool.

Two MR studies assessed the relation between alcohol consumption and liver function through biomarkers. Using variants of *ADH1B* and *ADH1C* as IVs in 58,313 individuals of Danish origin from the Copenhagen General Population Study (CGPS), Lawlor et al. (112) reported that amongst those who drank any alcohol, positive but not significant association were observed with higher alanine aminotransferase (ALT, mean difference per doubling of alcohol consumption = 3.7% [−4.5–11.9], y-glutamyl-transferase (GGT) = 6.8% [−2.8–16.5], while these associations were significant in their observational multivariable analyses. In contrast, a strong positive effect was found in MR analysis with alkaline phosphatase [ALP = 11.6% (6.8–16.4)] whereas the observational multivariable analysis suggested a weak inverse association. A more recent study used the *ADH1B-*rs1229984 as IV for assessing alcohol drinking. The association between genetically predicted alcohol consumption was not significant with ALT and GGT levels (whereas it was significant in their multivariate observation analysis). It was however significant with the incidence of liver disease per 12 g alcohol/week [OR = 1.71 (1.38–2.13)] (113). These two studies utilized a one-sample design and validated two Mendelian randomization assumptions.

All these studies received good quality scores on the Q-Genie tool, and validated at least two MR assumptions.

#### 3.3.4 Neurological outcomes

Several MR studies assessed the link between alcohol consumption and cognitive performance. In a one-sample MR study by Almeida et al. (114), genetic instruments for alcohol consumption were used to investigate cognitive performance in a sample of 3,542 elderly men. The A allele of the *ADH1B*-rs1229984 was associated with lower prevalence of regular use of alcohol and decreased consumption among regular users. Although observed abstainers and irregular drinkers had higher odds of cognitive impairment (assessed with the Mini-Mental State Examination) than regular drinkers, the rs1229984-A polymorphism was not associated with a decreased odds of cognitive impairment [adjusted OR_AA/GG_ = 1.35 (0.29–6.27); OR_AG/GG_ = 1.05 (0.71–1.55)]. Similarly, no association was found with cognitive performance (assessed in terms of word recall, verbal fluency and processing speed) in another one-sample MR analysis using the same SNP in a population of mixed ancestry (115). Another one-sample MR study performed in Asian population and using the *ALDH2*-rs671 found no association between genetically-predicted alcohol consumption and cognitive function (assessed with word recall score and Mini-Mental State Examination) (116). A two-sample MR study using a set of 99 independent SNPs associated with the number of drinks per week in young adults also failed to show any association with cognitive functioning, assessed in terms of working memory, response inhibition and emotion recognition (117). Ritchie et al. (118) tested a different approach, in a gene × environment interaction study one-sample MR study. Cognitive ability was measured twice in 1,091 participants of the Lothian 1,936 birth cohort, at ages ∼11 and ∼70 years, using the IQ Moray House Test. Mean alcohol consumption was measured with a self-report instrument over the previous 2–3months before the cognitive test (recent alcohol consumption). A four-SNP score was used to assess ADH activity. Neither alcohol consumption (b = −0.14, *p* = 0.62) nor SNP score (*b* = 0.11, *p* = 0.82) were significant predictors of age ∼70 cognitive ability. However, a significant (albeit small) gene-environment interaction was found (*b* = −1.13, *p* = 0.007) showing that alcohol consumption interacted with the genotype score to significantly predict age ∼70 cognitive ability. In terms of methodological quality assessment, from these 5 studies on cognitive performance, only Au Yeung et al. (116) validated all three assumptions, and Ritchie et al. (118) was to be of good quality according to the Q-genie Tool.

Li et al. (119) 2024 comprehensively explored the causal associations of the common environmental factors with major Neurodegenerative Diseases (NDDs) including Alzheimer's disease (AD), Parkinson's disease (PD), amyotrophic lateral sclerosis (ALS), and multiple sclerosis (MS), based on updated large-scale genome-wide association study data through two-sample Mendelian randomization (MR) approach. The AD GWAS dataset from the International Genomics of Alzheimer's Project (IGAP) Stage 1 meta-analysis included 21,982 AD cases and 41,944 cognitively normal controls. The PD GWAS dataset was obtained from a meta-GWAS released by the International Parkinson's Disease Genomics Consortium (IPDGC) and included 33,674 PD cases and 449,056 controls mainly from three previous studies and 13 case-control studies The ALS GWAS dataset was derived from a large-scale multi-source meta-GWAS based on individual-level genotype data from 117 cohorts, including 27, 205 ALS cases and 110,881 controls. The MS GWAS dataset was from a meta-GWAS published by the International Multiple Sclerosis Genetics Consortium (IMSGC) based on 15 previous datasets and two large-scale independent datasets, including a total of 47,429 MS cases and 68,374 controls. Genetically predicted higher alcohol intake frequency was found to be associated with higher risk of MS [OR = 1.412 (1.130–1.765), *p* = 2.418 × 10^−3^], but lower risk of PD [OR = 0.724 (0.572–0.916), *p* = 7.174 × 10^−3^]. However, the genetically predicted drinking amount was not associated with any NDDs. The evidence of pleiotropy involving the causal association of alcohol intake frequency with MS suggested that this observed association may not be robust. Among the multiple types of alcohol consumption, higher average weekly beer plus cider intake, but not spirits or red wine, was causally associated with higher risk of AD [OR = 2.570 (1.389–4.757), *p* = 2.653 × 10^−3^]. This study validated all three MR assumptions, and was reported to be of good quality according to the G-genie Tool.

In studies focusing on Alzheimer's disease, Meng et al. (120) used genome-wide association data sourced from the UK Biobank (UKBB) GWAS summary statistics. The dataset analyzed 361,194 participants and included 13.7 million QC-passing SNPs. The numbers of SNPs ultimately identified as the instrumental variables were 89 (Alcohol intake frequency) and 30 (Alcoholic drinks per week). To identify genetic variants associated with AD prevalence, the authors utilized meta-analysis data from the International Genomics of Alzheimer's Project (IGAP). This dataset comprised 63,926 subjects, including 21,982 AD cases and 41,944 healthy controls of European origin. Neither alcohol intake frequency [OR = 0.923 (0.753–1.134), *p* = 0.364] nor alcoholic drinks per week [OR = 1.162 (0.803–1.678), *p* = 0.479] were associated with AD risk. Larsson et al. (121) performed an MR study using genetic variants associated with modifiable risk factors, including alcohol consumption, as instrumental variables. Summarized data for the associations between the genetic variants and risk factors for Alzheimer's disease were obtained from the International Genomics of Alzheimer's Project (IGAP), which included 17,008 cases of Alzheimer's disease and 37,154 controls. Genetically predicted alcohol consumption was negatively, albeit non-significantly, associated with Alzheimer's disease [OR 0.72 (0.50–1.04), *p* = 0.08]. Andrews et al. (122) assessed the relationship between alcohol consumption (number of drinks/week, *n* = 537,349 from a GWAS of the GSCAN; 55 SNPs), alcohol dependence (*n* = 46,568 from the PGC; 20 SNPs), AUDIT score (*n* = 121,604 from the UK Biobank study; 11 SNPs), and late-onset Alzheimer's disease (AD) (*n* = 17,008 cases and 37,154 controls from a meta-analysis of four studies) or AD age of onset survival (14,406 cases and 25,849 controls from the IGAP study) in individuals of European descent. No association was found with late-onset AD. However, genetically predicted alcohol consumption was associated with an earlier AD age of onset survival [HR = 2.02 (1.42–2.87), *p* = 9.4 × 10^−5^]. Additionally, individuals with 1-SD (1.90 drinks/week) higher consumption of alcohol are twice as likely to develop AD at a given point in time, resulting in a 66% probability of an earlier age of onset. These three studies on AD validated all three MR assumptions and were reported to be of good quality according to the G-genie Tool.

For Parkinson's disease, Grover et al. (123) examined causal associations between risky behavior phenotypes and Parkinson's disease using a Mendelian randomization approach. They used a two-sample Mendelian randomization to generate unconfounded estimates using summary statistics from two independent, large meta-analyses of genome-wide association studies on risk-taking behaviors (*n* = 370,771–939,908) and Parkinson's disease (cases *n* = 9,581; controls *n* = 33,245). Additionally, they utilized a recently published GWAS on alcohol consumption using data from 112,117 individuals from the UK Biobank. The effect estimates using different MR methods showed no significant association between genetically predicted weekly alcohol consumption (*n* = 941,280, 71 SNPs) and the risk of PD [OR = 1.15 (0.87–1.53); *p* = 0.325]. This finding of a suggestive absence of a causal association between alcohol consumption and PD was replicated using data from the UK Biobank [OR = 1.389 (0.110–17.563); *p* = 0.7621]. Heilbron et al. (124) performed a split-sample design MR analysis with customers of 23andMe, Inc., a personal genomics company. The analysis included 19,924 PD cases and 2,413,087 controls. The diagnosis of PD was self-reported. A genome-wide association study was performed to identify single nucleotide polymorphisms associated with alcohol intake. Higher daily alcohol intake increased the risk of PD [OR = 1.125 (1.025–1.235); *p* = 0.013]. Notable limitations were that phenotypes (especially PD) were constructed using self-reported data derived from online surveys, which may suffer from recall bias and desirability bias. Additionally, the individuals studied were not a random sample of the general population, potentially leading to selection bias. Domenighetti et al. (125) examined associations of lifestyle behaviors, including alcohol drinking, with PD using two-sample MR and investigated the potential for survival and incidence-prevalence biases. They used summary statistics from publicly available studies to estimate the association of genetic polymorphisms with lifestyle behaviors and from Courage-PD (7,369 cases, 7,018 controls; European ancestry) to estimate the association of these variants with PD. The GWAS and Sequencing Consortium of Alcohol and Nicotine use (GSCAN) provided summary statistics for the number of alcohol drinks per week (*n* = 941,280; 71 SNPs) in participants of European descent. There was a non-significant negative association between alcohol drinking and PD [OR IVW = 0.68 (0.39–1.18), *p* = 0.17]. Finally, Domínguez Baleón et al. (126) conducted a two-sample Mendelian randomization study using genome-wide association study summary statistics from the GWAS & Sequencing Consortium of Alcohol and Nicotine use study (1.2 million participants) and the latest meta-analysis from the International Parkinson's Disease Genomics Consortium (37,688 PD cases and 18,618 proxy-cases). The analysis revealed a significant association of genetically predicted alcohol intake with lower PD risk [OR = 0.79 (0.65–0.96); *p* = 0.021]. Multivariable MR analyses showed that the causal association between drinks per week and PD is unlikely due to confounding by smoking behavior. Frailty analyses suggested that the causal effect of alcohol intake on PD risk estimated from MR analysis is not explained by the presence of survival bias alone. These four studies on PD validated all three MR assumptions and were reported to be of good quality according to the G-genie Tool.

In examining ALS, Yu et al. (127) used a large GWAS consisting of 20,806 cases and 59,804 controls. Using 44 SNPs from a GWAS that included 480,842 individuals of European descent, they found a significant association between genetically predicted alcohol consumption and ALS [OR per 10 g/day = 2.48 (1.38–4.44); *p* = 0.002]. This association remained significant after adjusting for smoking in a multivariable MR analysis [OR = 2.23 (1.06–4.70); *p* = 0.040]. This study validated all three MR assumptions and was reported to be of good quality according to the G-genie Tool.

Wang et al. (128) conducted an MR study to evaluate the association between genetically predicted alcohol consumption and earlier age at onset (AAO) of Huntington's disease (HD). They selected genetic instruments for alcohol consumption (*n* = 941,280) based on two large genome-wide association studies (GWAS). The summary-level data for residual AAO of HD were derived from a GWAS meta-analysis carried out by the Genetic Modifiers of Huntington's Disease Consortium (*n* = 9,064 HD patients). Univariable and multivariable MR analyses evaluated the independent impact of smoking and alcohol consumption on AAO of HD. No significant association was found between alcohol consumption and AAO of HD. This study validated all three MR assumptions and was reported to be of good quality according to the G-genie Tool.

Two studies investigated the relationship between alcohol consumption and epilepsy. Zhang et al. (129) conducted a two-sample MR study where genetic variants associated with alcohol intake were adopted as instrumental variables. Summary data for epilepsy were obtained from the International League Against Epilepsy (ILAE) Consortium (15,212 cases and 29,677 controls) and the FinnGen consortium (4,588 cases and 144,780 controls). Based on findings reported from the Consortium of Alcohol and Nicotine Use (GSCAN), 84 independent SNPs were adopted as instrumental variables for alcohol intake. Combined analysis of the ILAE and FinnGen databases indicated that genetically predicted alcohol intake was associated with a higher risk of epilepsy [OR = 1.24 (1.06–1.47); *p* = 0.009]. Yuan et al. (130) also conducted a two-sample MR study. Summary-level data for epilepsy were obtained from the FinnGen consortium (4,588 cases; 144,780 controls). Potential causal associations (*p* < 0.05) were attempted for replication using UK Biobank data (901 cases and 395,209 controls). Although positive, the association observed for genetically predicted alcohol consumption was not significant [OR = 1.54 (0.99–2.41), *p* = 0.058]. Both studies assessing epilepsy validated all three MR assumptions and were reported to be of good quality according to the G-genie Tool.

## 4 Discussion

In this systematic review, we examined 70 MR studies that investigated the causal relationships between alcohol consumption and cancers (26 studies), cardiovascular (24 studies), liver (6 studies) and neurological (17 studies) diseases. Observational studies have suggested that any level of alcohol consumption poses a risk to health, and the potential protective effects of low levels of consumption may be influenced by confounding factors and methodological biases (131). With the advantage of a large number of genetic variants involved in alcohol metabolism and consumption, we deemed it important to analyze the latest data from MR studies on the effects of alcohol on health outcomes.

### 4.1 Cancers

Our review on the relationship between genetically predicted alcohol consumption and various cancers provides important insights, yet reveals a complex and heterogeneous set of findings.

The association of genetically-predicted alcohol consumption with cancer was assessed for all cancers in one study (60), for breast and ovarian cancers in five additional studies (62–66) and for endometrial cancer in one study (67).

Regarding breast cancer, the five MR analyzes reported no significant association except with genetically predicted problematic alcohol use (60, 62, 63, 65, 66). These results were in contradiction with the findings of observational studies showing that any drinking, starting from light to moderate alcohol consumption is associated with a dose-dependent increase in breast cancer risk (62, 132, 133). However, it is crucial to note that the MR study may have lacked sufficient statistical power to identify such a moderately sized association, as demonstrated in other studies, where there is an 8% to 12% increase in risk for each 10 g/day increment in alcohol consumption, as some authors have suggested (68). As for ovarian cancer, MR findings were consistent with those of observational studies suggesting no association between alcohol consumption and ovarian cancer (134, 135).

For endometrial cancer, MR analysis indicates a strong causal relationship between genetically predicted alcohol consumption and EC risk. Specifically, highlighting the protective effect of genetically predicted alcohol. Subgroup analyses revealed that genetically predicted alcohol consumption had a protective effect only in the endometrioid subtype of endometrial cancer (EEC), while no protective effect was observed in non-endometrioid endometrial cancer (NEC). The study suggests that the protective effect of alcohol consumption on EC is mediated by other factors, specifically a decrease in human chorionic gonadotropin (HCG), which is associated with endometrial proliferation and malignant tumors (136), and insulin-like growth factor 1 (IGF1), which is involved in the occurrence and development of EC (137).

This relationship has been controversial in observational studies. A prospective cohort study involving 41,574 participants found that daily alcohol consumption of ≥2 drinks/day increased postmenopausal EC risk [RR = 2.01 (1.3–3.11)] compared to no alcohol consumption (138). Conversely, another prospective Nurses’ Health Study by Je et al. (139) in a population of 68 067 female participants showed a decreased risk of endometrial cancer for light alcohol consumption. Women with an alcohol intake of less than 5 g per day had a 22% lower risk of endometrial cancer. When comparing categories of alcohol intake and risk of endometrial cancer, the evidence remains inconsistent. A meta-analysis by Bagnardi et al. (20), based on results from 21 observational studies, found no significant association between endometrial cancer risk and moderate (≤50 g per day) or light (≤12.5 g per day) alcohol intake compared to no alcohol intake.

The relationship between alcohol consumption and prostate cancer risk remains inconclusive in observational studies (140). While some studies suggest a potential increased risk among heavy drinkers (141), others find no significant association, particularly for low-to-moderate consumption (142). In contrast to these mixed findings, the two MR studies reported no significant association between genetically-predicted alcohol consumption and prostate cancer. However, alcohol consumption was associated with increased prostate cancer mortality in men with low-grade prostate cancer (60, 68).

Oral/oropharyngeal cancers is the site-specific cancer clearly associated with genetically-predicted alcohol consumption in our review (69). Importantly, this association remained significant even after adjusting for smoking, demonstrating alcohol's independent effect which contrasts observational studies suggesting alcohol interacts synergistically with smoking to elevate the risk of head and neck cancer (143). Observational studies have identified alcohol as a significant risk factor for head and neck cancer (144, 145). Genetic factors, such as slow ADH1B and ALDH2 enzyme activity, further increase susceptibility to alcohol-induced HNC (146). Biochemical studies further elucidate this relationship, showing that alcohol's toxicity involves its metabolic products, oral microbiota, and oxidative stress, leading to genetic and epigenetic alterations (147), suggesting carcinogenic effects of alcohol are mediated not only through systemic exposure but also through local factors (148).

The results regarding the relationship between alcohol consumption and lung cancer were contradictory. One (72) of four MR studies (60, 71–73) examining the effects on lung cancer suggested that genetically predicted habitual alcohol consumption with meals (also described as light to moderate) was a protective factor against lung cancer and squamous cell cancer lung. This alcohol consumption during meals was unrelated to lung adenocarcinoma or small cell lung cancer in the MR analysis. Similarly, another MR study suggested a decreased risk in both men and women although it was statistically insignificant in women. These results confirm observational studies supporting the idea that light to moderate alcohol consumption may prevent lung cancer, most prominently squamous cell carcinoma, and increase survival after the diagnosis (18, 149). In contrast, In Larsson et al. (60), genetically predicted alcohol drinking was significantly positively associated with lung cancer in the ILCCO but not in the UK-Biobank study. In any case, the findings reported by this study call into question a protective effect of alcohol consumption on the onset of lung cancer while another study reported no association (73). However, the association between alcohol consumption and lung cancer is still under debate for never-smokers (150).

Regarding esophageal cancer, results from the six studies were contradictory. While four studies reported a positive association between alcohol intake and esophageal risk (60, 71, 74, 76), another study reported no association with genetically predicted alcohol frequency (75). Similarly, Zhang et al. (77) reported no association with esophageal squamous cell carcinoma, however, they did find a significantly increased risk associated with the variant rs1229984 of ADH1B. Despite these discrepancies, this set of six MR studies supports previous observational studies. A dose-response meta-analysis indicated that alcohol intake significantly increases the incidence of esophageal cancer, especially for ESCC (151). Additionally, another recent dose-response meta-analysis reported that even light alcohol consumption is significantly associated with higher risks of esophageal cancer, extending beyond heavy alcohol consumption (152).

As for gastric cancer, the set of five studies overall provides little support for a causal relationship between genetically predicted alcohol consumption and the risk of gastric cancer. Four out of the five studies reported no significant association. Yuan et al. (74) did report a positive, though non-significant, association even after adjusting for smoking. Interestingly, observational data showed a similar direction in a recent meta-analysis of eighty-one epidemiological studies demonstrated that increased daily intake of alcohol was correlated with a heightened incidence of gastric cancer (153).

Seven studies assessed the association between genetically predicted alcohol intake and colorectal cancer. Of these, five studies reported an increased risk, with one study maintaining a positive association even after adjusting for smoking (74). While one study reported no association with current, never and previous alcohol intake (80), another found a negative, though non-significant, association (76). Overall, these results align with observational studies suggesting that alcohol consumption is a risk factor for colorectal cancer, particularly in Asian populations. A case-control study conducted in an Asian population revealed that being a current or former drinker was positively associated with the risk of CRC compared to controls [OR = 5.4 (1.1–27.8), *p* = 0.043] (154). Moreover, an updated meta-analysis of prospective cohort studies by Zhou et al. (79) demonstrated that moderate and heavy drinking were statistically significantly associated with an increased risk of colorectal cancer.

The association between genetically predicted alcohol consumption and the risk of liver cancers was analyzed in six MR studies. Consistent with previous observational studies, the MR studies demonstrated that genetically predicted alcohol consumption increases the risk of hepatocellular carcinoma. Notably, one of these studies reported a non-linear relationship, finding a positive association between pure alcohol intake (g/day) and the risk of HCC, with a statistically significant linear trend (*p* < 0.0001). However, these studies showed positive but non-statistically significant associations with liver cancer overall. A similar positive relationship between alcohol consumption and HCC risk in Chinese populations was reported in a meta-analysis of 18 case-control studies, which included 3,812 HCC cases and 10,927 controls. The meta-analysis found that ever drinkers had a significantly higher risk of HCC compared to never drinkers (OR = 1.56; 95% CI, 1.16–2.09) (155). Another meta-analysis of 11 case-control studies indicated a positive association between alcohol consumption and liver cancer risk, with a higher intake increasing the risk compared to lower intake (156). The underlying mechanisms explaining this association in previous studies are multifaceted. Acetaldehyde, the first metabolic product of alcohol, can induce oxidative stress and DNA damage, promoting carcinogenesis. Furthermore, chronic and heavy alcohol consumption can lead to alcoholic cirrhosis, a known precursor to HCC. Moreover, alcohol acts as a solvent, enhancing the penetration of other carcinogens into liver cells, while also impairing hepatic detoxification and immunity (156, 157).

As for pancreatic cancer, all three studies from our review did not show any significant causal association with genetically predicted alcohol drinking. These findings contrast with previous observational studies, which reported an increased risk of pancreatic cancer only with heavy drinking (158, 159) likely mediated by inflammatory pathways related to chronic pancreatitis (160).

Also, our review found no relationship between alcohol consumption and bladder cancer risk. The link between alcohol consumption and bladder cancer risk has been inconsistent in observational studies, with varying results likely due to the type of alcohol consumed (e.g., beer, wine, or spirits) rather than the amount of alcohol consumed (161, 162). There was a linear dose-response relation in those who consume alcohol from liquor or spirits (163).

Conversely, a statistically significant increased risk of cutaneous melanoma was observed, aligning with observational studies that reported an increased risk even with moderate alcohol intake. Ethanol has been shown to inhibit the developmental process of melanocytes, directly contributing to the progression of melanoma, it also enhances its metastatic ability (164–166).

Contrary to observational studies suggesting an association between moderate alcohol consumption and a lower risk of thyroid and biliary tract cancers (167, 168), our systematic review did not confirm any significant association.

Finally, genetically predicted alcohol consumption was not associated to overall cancer in the only study having all cancers as outcomes (60). This lack of association is likely to be reliable to some extent as the study showed good methodological qualities.

Overall, MR studies suggest that genetically predicted alcohol consumption is associated with an increased risk for certain cancers, particularly oral/oropharyngeal, lung, head and neck, cutaneous melanoma, colorectal, and liver cancers particularly hepatocellular carcinoma. For breast cancer, a statistically significant increased risk was only observed in association with genetically predicted problematic alcohol use. for esophageal cancer, a statistically significant association was reported in the two studies performed on Asian ancestry population. Particularly, an increased risk for esophageal squamous cell carcinoma (ESCC) in association with the ADH1B rs1229984 variant. In contrast, no significant association was found for ovarian, prostate, lung, esophageal, gastric, pancreatic, bladder, thyroid, kidney, non-Hodgkin lymphoma, biliary tract cancers. Endometrial cancer showed a unique protective effect particularly in the endometrioid subtype.

These findings highlight the complex and varied relationship between alcohol consumption and cancer risk. The inconsistencies in the results can be attributed to differences in population ancestry, sample size, and genetic variants across the studies. To obtain more definitive conclusions, it is essential to conduct studies with more generalized populations. Additionally, it is important to note that we were unable to assess possible U- or J-shaped relationships between alcohol consumption and cancer risk in this review. Future research should aim to address these gaps and explore the potential non-linear associations between alcohol consumption and cancer.

### 4.2 Cardiovascular outcomes

The relationship between alcohol drinking and CVD is complex, controversial and still highly debated (169). There have been a growing number of studies outlining both harmful and potentially protective effects of alcohol consumption on the risk of various cardiovascular outcomes but there is less clarity about estimates of risk with low levels of consumption (131). Observational studies have consistently reported that compared with non-drinkers, light to moderate drinking exhibits a reduced cardiovascular risk (170, 171). However, relying on the participants’ self-report, imply the possibility of reverse causality and confounding; for example, Schutte et al. demonstrated that using never drinkers as reference entails a bias leading to underestimating the cardiovascular risk. When considering the drinking reference bias and excluding ischemic heart disease and wine beverage from the analysis, there is no overall cardiovascular protection from alcohol and instead there is an association with increased cardiovascular risk even when consuming 112 g or less per week (172).

Although two meta-analyses of observational studies reported an association of light or moderate alcohol consumption with a reduced risk of heart failure (26, 173), two large two-sample MR analyzes that validated all three key assumptions and reported a good quality according to the Q-genie tool) found no association between genetically predicted alcohol intake and the risk of heart failure (40, 105). As these two MR studies did not distinguish between levels of alcohol consumption, it is difficult to confront their findings with those deriving from the observational studies.

Five MR studies examined alcohol intake and hypertension (100–102, 174, 175). All these MR studies showed a significant association of alcohol consumption with an increased risk of hypertension, with the exception of a Chinese study where no association was found in women, a result that may derive, as suggested by the authors, from the fact that Chinese culture does not encourage women to drink (101). As these MR studies were not designed to reveal nonlinear associations, no conclusion could be reached regarding the light-moderate alcohol use possible causal effect on hypertension. These findings are consistent with two recent meta-analyses of cohort studies showing an elevated risk of hypertension compared to abstainers for any amount of alcohol consumption in men, and above 20–24 g per day in women (176, 177). In addition, it has been shown that a reduction in alcohol consumption was associated with a decrease of blood pressure in a dose-dependent manner with an apparent threshold effect at 24 g of alcohol per day. Overall, the convergent findings of MR and observational studies findings supports the hypothesis of causal effect of alcohol consumption on blood pressure and hypertension risk, particularly in men.

Four MR studies investigated the association of genetically predicted alcohol consumption with CHD (including coronary artery disease and myocardial infarction) (40, 91, 95, 104). These studies mostly showed non-significant associations between CHD and genetically predicted alcohol consumption when adjusted to smoking (40, 104). One study (91) showed that a genetic variant associated with non-drinking and lower alcohol consumption had a reduced risk of CHD, an association that disappeared when the analysis was restricted to non-drinkers.

Two MR studies in our review investigated the association of genetically predicted alcohol consumption with AF. The two-sample MR study conducted by Jiang et al. (107) reported no significant association between genetically predicted alcohol consumption and the risk of AF. As this MR study did not distinguish between levels of alcohol consumption, it is difficult to confront its negative findings with those deriving from the observational studies, showing an association between heavy alcohol consumption and an increased risk of AF (178). While Larsson et al. suggested a positive significant association between genetically predicted alcohol consumption and AF [OR_IVW_, 1.17, (1.00–1.37); *p* = 0.05], the association was no longer significant after adjustment for smoking initiation (40). All four MR studies that investigated stroke reported a positive association with genetically predicted alcohol consumption (40, 88, 91, 95). One study was particularly methodologically sound, and showed that the association resisted to the adjustment to smoking initiation (40). Another study (91) showed that a variant associated with non-drinking and lower alcohol consumption was associated with an ischemic stroke (but not with the combined subtypes of stroke), an association that disappeared when the analysis was restricted to non-drinkers. Finally, in another study performed in a Chinese population, the MR analysis showed a log-linear rather than a U-shaped association with stroke, advocating against a possible protective effect of light or moderate alcohol consumption on stroke (95).

Finally, MR studies clearly confirmed the causal effect of alcohol on the risk of increased blood pressure, hypertension, and stroke. Beyond this, as most MR studies were unable distinguish between levels of alcohol consumption, the negative findings were difficult to confront with observational studies claiming a protective effect of light or moderate alcohol consumption. There is however an exception deriving from the study from Millwood and colleagues (95) in Asian descents, where the MR analysis showed a log-linear rather than the U-shaped association found in the conventional epidemiology analysis with stroke (in men only). Thus, demonstrating that MR methodology can call into question the results from classical observational methodology when applied to the same sample.

Several MR studies have challenged the previously suggested protective effect of moderate alcohol consumption on cardiovascular disease.

Traditional observational studies had shown a potential association between moderate alcohol consumption and a reduced risk of CVD, particularly CHD. However, these studies were subject to confounding factors, such as lifestyle, diet, socioeconomic status, and reverse causality, which may have biased the results.

Several MR studies have found that the protective effects of alcohol on heart health may be overstated or nonexistent. The study of Holmes et al. (91) used genetic variants related to alcohol metabolism (particularly the ADH1B gene) to examine the causal relationship between alcohol consumption and cardiovascular health. The study found that individuals with genetic variants that lead to lower alcohol consumption had a reduced risk of coronary heart disease. This suggests that lower alcohol intake, rather than moderate consumption, is beneficial for heart health. The study did not support a protective effect of moderate drinking on CVD risk. In the MR study of Millwood et al. (95), alcohol consumption was associated with an increased risk of stroke and other cardiovascular events, even at relatively low levels. There was no protective effect observed, and the study concluded that alcohol consumption increases the risk of CVD. Larsson et al. (40) found no evidence of a protective effect of moderate alcohol consumption on coronary artery disease. Instead, the study pointed to harmful effects of alcohol on heart health.

These studies suggest that the supposed cardiovascular benefits of moderate alcohol consumption may have been overstated in earlier observational research, largely due to confounding variables. Mendelian randomization, being less prone to confounding, provides stronger evidence that alcohol consumption likely does not confer a protective effect on CVD risk and may in fact be harmful, even in moderate amounts.

### 4.3 Liver outcomes

Only a few MR studies have been published on liver outcomes in relation to genetically predicted alcohol intake. Our review highlighted a strong association between genetically predicted alcohol consumption and alcoholic liver disease (ALD), with a markedly increased risk consistently reported across studies. Furthermore, significant positive associations were found between alcohol consumption and cirrhosis, indicating that higher alcohol intake significantly elevates the risk of developing these severe liver conditions.

Regarding MASLD, the three studies showed mixed results. Sookoian et al. (111) found that even moderate alcohol consumption could be harmful in patients at high risk for progressing to end-stage liver disease, with lower alcohol consumption correlating with less severe histological features. Despite the small sample size and the modest level of alcohol consumption due to MASLD inclusion criteria, carriers of the A-allele exhibited lower degrees of histological steatosis, lobular inflammation, and MASLD-Activity Score compared to non-carriers. While, Yuan et al. (112) reported a significant inverse association between genetically predicted alcohol consumption and the risk of MASLD. Nonetheless, the findings require further validation due to moderate-to-high heterogeneity and potential pleiotropy in the genetic instruments. Finally, another reported no significant association with the risk of MASLD. These results contradict those of previous observational studies indicating that, compared to nondrinkers, people who drink moderate levels of alcohol (≤30 g/day) not only have a lower prevalence of MASLD, but also less severe disease from a histological standpoint (179).

Two MR studies investigated the impact of alcohol consumption on liver function biomarkers. While non-significant positive associations were observed with alanine aminotransferase (ALT) and gamma-glutamyl transferase (GGT) in one study, significant positive associations were found with alkaline phosphatase (ALP). These findings indicate potential liver function impairment linked to alcohol consumption. Another study also reported significant associations between alcohol consumption and the incidence of liver disease, despite non-significant results for ALT and GGT levels. These results are consistent with observational studies indicating that even moderate drinking can elevate γ-GT, ALT, and ferritin levels compared to abstainers (180).

In summary, while the available MR studies provide some insights into the link between alcohol consumption and liver disease, the results are limited and varied. It is crucial to note that no studies tested for non-linearity in these associations, highlighting a significant gap in the current understanding that needs to be addressed in future research.

### 4.4 Neurological outcomes

The association of alcohol consumption with cognitive outcomes is controversial. Epidemiological studies have repeatedly reported better brain health in moderate drinkers compared with abstainers. Observational studies suggest that heavy alcohol consumption leads to deterioration of cognitive and executive function and is related to an increased risk of dementia (37) and other neurodegenerative diseases (181),while moderate alcohol intake may have a protective effect, leading to a J-shaped or U-shaped relationship (182–184). Overall, concerns about confounding and inconsistencies between epidemiological studies make it difficult to define what level of intake may have beneficial effects for cognition.

We reviewed MR studies with cognitive performance as an outcome and found that the methodological quality was generally low, with relatively small sample sizes. Although some studies observed an association between alcohol consumption and better cognitive performance, the five MR analyses did not confirm a causal relationship between genetically predicted alcohol consumption and cognitive performance. One study employed an additional approach, conducting a gene × environment interaction analysis (118). This study found that alcohol consumption interacted with the genotype score reflecting ADH activity to significantly predict cognitive ability around age 70. The authors interpreted this finding to mean that individuals with high alcohol processing efficiency experienced modest improvements in intelligence with increased alcohol consumption later in life. In contrast, individuals with lower alcohol processing efficiency experienced a decline in cognitive ability with greater alcohol consumption throughout their life. However, it should be noted that this study had a number of limitations, including a limited sample size, and the latter finding is not the result of an MR analysis, falling somewhat outside the scope of our review. In any case, these MR studies do not support the causal effect of alcohol consumption suggested by observational findings that moderate levels of alcohol consumption are associated with better cognitive performance (183, 184).

Four MR studies examined the association between genetically predicted alcohol consumption and AD. In all studies, the associations were generally non-significant, albeit mostly in the negative direction. However, a significant association was reported between average beer plus cider consumption and a higher risk of AD (119). It should be noted, however, that although these studies were of good methodological quality, they seemed to use an overlapping set of subjects from the IGAP. Here again, we find no support for a possible protective effect of alcohol consumption on the risk of developing AD.

Five studies examined the association between genetically predicted alcohol consumption and PD. Two studies reported a protective effect of genetically predicted alcohol use on the risk of developing PD. In one study, the protective effect was observed only with alcohol intake frequency (not alcohol amount) (119). Importantly, the other study demonstrated that the protective effect of alcohol intake was unlikely to be due to confounding by smoking behavior or the presence of survival bias (126). Two studies reported no association of genetically predicted alcohol use with the risk of developing PD, while one study reported a positive association of genetically predicted alcohol use with the risk of developing PD (124). It should be noted that this latter study had notable methodological shortcomings. Despite some discrepancies, this set of five MR studies provides some support for a possible protective effect of alcohol use on the risk of developing PD, as suggested by the findings of a meta-analysis of case-control studies (185).

An MR analysis of the association between genetically predicted alcohol consumption and ALS was performed in two studies. One of these studies reported a positive association, which remained significant after adjusting for smoking (127). This latter finding suggests a causal detrimental effect of alcohol consumption on the risk of developing ALS, contrasting with the findings of a meta-analysis of five observational studies that showed a potentially neuroprotective effect of alcohol (186). One study reported a positive association of genetically predicted higher alcohol intake frequency with a higher risk of MS, contrasting with the report of a dose-dependent inverse association of observed alcohol consumption with MS (187). In one MR study (128), no significant association was found between genetically predicted alcohol consumption and the age of onset of Huntington's Disease, a finding that does not help to clarify the conclusions of a few observational studies, which reported contrasting results (128).

The association between genetically predicted alcohol consumption and the risk of epilepsy was analyzed in two MR studies. Both studies reported a positive association, reaching statistical significance in only one (129). These results suggest a causal detrimental effect of alcohol on the risk of developing epilepsy, providing clarity in an area with previously inconsistent associations. While mild to moderate alcohol consumption may decrease the risk of seizures, alcohol consumption is generally thought to lower the seizure threshold, and alcohol withdrawal is a recognized cause of seizures (188).

In summary, the findings from MR studies on alcohol consumption and neurological outcomes provide mixed results. While some studies suggest potential protective effects of moderate alcohol consumption on certain neurodegenerative diseases, others highlight detrimental associations, particularly with higher intake frequencies. The methodological limitations and inconsistencies observed in these studies underscore the need for more robust and large-scale research to clarify these associations. Overall, these MR studies do not provide strong evidence to support the protective effects of alcohol consumption suggested by some observational studies, and they highlight the complex and multifaceted nature of alcohol's impact on brain health.

### 4.5 Strengths and limitations of the study

This systematic review has some limitations. First, the lack of an existing formal quality assessment tool to assess the quality of MR studies may limit the strength of our findings (189). To minimize this limitation, we computed our validation protocol for MR analyses based on the STROBE-MR (Strengthening The Reporting of Observational studies in Epidemiology using Mendelian Randomization) check list (190), which is inspired by the original STROBE checklist and developed to assist researchers in reporting their MR studies clearly and transparently.

Mendelian randomization studies also display some methodological limits and, as other studies, may lack power. Some genotypes used in MR studies may not be strong instruments due to limited variation in alcohol intake that could be explained by theses genotypes in a specific population. For instance, genotypes of *ADH* (fast alcohol metabolizers) and *ALDH* (lower acetaldehyde clearance rate) are associated with alcohol intake because higher acetaldehyde levels lead to unpleasant symptoms, but peak levels of acetaldehyde are also involved in the development of alcohol-related cancers (191, 192) and possibly CVD (193). Therefore, the level of alcohol drinking cannot be studied independently of acetaldehyde. Moreover, the available studies included in our systematic review are often heterogeneous, including variations in genetic IVs used, adjustments for confounding exposures, the study populations, and the outcome measurements. However, we included a large number of studies, with many two-sample MR design. These patterns of MR confer a higher statistical power and use a large number of independent IVs that come from the biggest GWAS of alcohol to date (from the GSCAN consortium (61) which increases the proportion of the variance of alcohol consumption explained by the IVs. The majority of the MR studies found consistent results, which provides an additional level of evidence for the role of alcohol consumption in the occurrence of these diseases.

Unfortunately, genetic variants are often unable to clearly distinguish between abstainers, light drinkers, and moderate drinkers, making it difficult to distinguish between U or J-shaped vs. linear relationships between exposure and outcome. As one of the controversies regarding the impact of alcohol consumption concerns the possible protective effect of light or moderate alcohol consumption on health outcomes, particularly cardiovascular outcomes, our systematic review does not allow us to draw clear conclusions regarding the causal effect of moderate alcohol consumption on these outcomes.

To address these limitations, further research is needed to develop formal quality assessment tools for MR studies and to conduct more MR studies on diverse populations, including those with non-European ancestries.

## 5 Conclusion

Our systematic review of MR studies sheds new light on the complex relationship between alcohol consumption and various health outcomes. The available studies did not confirm the protective effect of alcohol on lung cancer suggested by observational studies, while it showed a positive significant association on multiple cancers like oral and oropharyngeal, esophageal, colorectal cancers, hepatocellular carcinoma and cutaneous melanoma. Furthermore, MR studies were able to confirm the causal effect of alcohol on the risk of increased risk of hypertension, stroke, atrial fibrillation and myocardial infraction. Several MR studies have found that the protective effects of alcohol on heart health may be overstated or nonexistent. Our systematic review also found limited evidence to support the protective effects of light to moderate alcohol consumption on cognitive function, AD, and amyotrophic lateral sclerosis, as previously reported in observational studies while it showed a positive association with epilepsy and multiple sclerosis. Finally, the available studies provided only limited results on the link between alcohol consumption and liver disease. However, a limitation of genetic instrument is their inability to consistently distinguish between abstainers, light drinkers, and moderate drinkers, which can complicate efforts to differentiate between U or J-shaped vs. linear relationships between exposure and outcomes.

Overall, our review suggests that MR studies can provide valuable insights into the causal relationship between alcohol consumption and various health outcomes, but further research is needed to explore the potential U- or J-shaped relationships between alcohol consumption and cancer.

As the evidence continues to evolve, it will be important to consider the potential benefits and risks of alcohol consumption in the context of individual and population health, and to inform evidence-based policy and practice.

## Data Availability

All data used in this systematic review are derived from previously published studies, which are available through the cited literature.
