# Supplementary material for: Alcohol consumption and its association with cancer, cardiovascular, liver and brain diseases: a systematic review of Mendelian randomization studies
Source: Front Epidemiol. 2024 Nov 7;4:1385064. doi: 10.3389/fepid.2024.1385064 (PMC11578756; doi:10.3389/fepid.2024.1385064)
Supplement: Supplementary file 1 [file Datasheet1.pdf]

Supplementary materials

List of Supplementary Materials	Page
Supplementary Table 1. Research equations	3
Supplementary Table 2. Risk of bias assessment of included studies evaluating genetically predicted alcohol consumption in relation to Cancers using Q-genie Tool	4_5
Supplementary Table 3. Risk of bias assessment of included studies evaluating genetically predicted alcohol consumption in relation to cardiovascular outcomes using Q-genie Tool	6_7
Supplementary Table 4. Risk of bias assessment of included studies evaluating genetically predicted alcohol consumption in relation to brain outcomes using Q-genie Tool	8
Supplementary Table 5. Risk of bias assessment of included studies evaluating genetically predicted alcohol consumption in relation to liver outcomes using Q-genie Tool	9
Supplementary Table 6. Methodological quality assessment through testing for assumptions: Genetically predicted alcohol and cancer outcomes	10_11
Supplementary Table 7. Methodological quality assessment through testing for assumptions: Genetically predicted alcohol and cardiovascular outcomes	12_13
Supplementary Table 8. Methodological quality assessment through testing for assumptions: Genetically predicted alcohol and brain diseases	14_15
Supplementary Table 9. Methodological quality assessment through testing for assumptions: Genetically predicted alcohol and liver outcomes	15
Supplementary Table 10. Characteristics and results of included mendelian randomization studies investigating genetically predicted alcohol consumption and cancer outcomes	16_17_18_19_20_21
Supplementary Table 11. Characteristics and results of included mendelian randomization studies investigating genetically predicted alcohol consumption and cardiovascular outcomes	22_23_24_25_26_27

Supplementary Table 12. Characteristics and results of included mendelian randomization studies investigating genetically predicted alcohol consumption and brain diseases	28_29_30
Supplementary Table 13. Characteristics and results of included mendelian randomization studies investigating genetically predicted alcohol consumption and liver outcomes	31_32

Supplementary Table 1. Research equations

Database	Search Method	Search Terms Used
PubMed	Free text	« (“Alcohol” OR “ethanol “) AND (“Mendelian Randomization” OR “Genetic epidemiology”) AND (“cancer ” OR “cardiovascular disease” OR “Neurological disease” OR “Liver disease”) »
	Mesh Terms	«(“Alcohol [Mesh Terms] “AND (“Mendelian Randomization [Mesh Terms]”) AND (“Cancer [Mesh Terms]”OR “Cardiovascular disease [Mesh Terms]” OR “Neurological disease” [Mesh Terms]”OR “Liver disease” [Mesh Terms]”) »
ScienceDirect	Free text	«("Alcohol” OR “ethanol") AND ("Mendelian Randomization OR Genetic epidemiology") AND (“cancer” OR "cardiovascular disease” OR “Neurological disease” OR “Liver disease")»
Embase	Emtree Terms	("Alcohol [Emtree Terms]" AND "Mendelian Randomization [Emtree Terms]" AND ("Cancer” OR “Cardiovascular disease” OR “Neurological disease” OR “Liver disease")
Europe PMC	Free Text	("Alcohol consumption" AND ("Mendelian Randomization” OR “Instrumental variable analysis") AND ("Cancer” OR “Cardiovascular disease” OR “Neurological disease” OR “Liver disease")

Supplementary Table 2. Risk of bias assessment of included studies evaluating genetically predicted alcohol consumption in relation to Cancers using Q-genie Tool

Quality indicator[a] / *Reference*	*Rationale for study*	*Selection and definition of outcome of interest*	*Selection and comparability of comparison groups*	*Technical classification of the exposure*	*Non-technical classification of the exposure*	*Other sources of bias*	*Sample size and power*	*A priori planning of analyses*	*Statistical methods and control for confounding*	*Testing of assumptions and inferences for genetic analyses*	*Appropriateness of inferences drawn from results*	*Total score*[b]	*Overall rating*[c]
Gormley et al,2020 (1)	5	5	3	5	5	3	5	5	5	5	5	51	Good
Zhu et al,2020 (2)	5	3	3	5	3	3	5	5	5	5	5	47	Good
Larsson et al,2020 (3)	5	5	3	5	1	3	5	5	5	5	5	47	Good
Brunner et al,2019 (4)	7	7	3	5	3	7	5	5	3	1	5	51	Good
Ong et al,2021 (5)	5	3	3	5	1	5	7	7	5	5	5	51	Good
Chen et al,2021(6)	7	5	3	5	3	5	5	5	5	5	5	53	Good
Zhou et al.2022 (7)	6	6	5	6	5	5	5	5	5	5	5	58	Good
Zhou et al.2022 (8)	6	6	5	6	5	5	5	5	5	5	5	58	Good

[a] Scores range from 1 (poor) to 7 (excellent)

[b] Higher scores indicate higher overall quality

[c] Overall rating of poor, moderate or good

For studies with control groups: Scores ≤35 indicate poor quality studies, >35 and ≤45 indicate studies of moderate quality, and >45 indicate good quality studies.

For studies without control groups: Scores ≤32 indicate poor quality studies, >32 and ≤40 indicate studies of moderate quality, and >40 indicate good quality studies.

NA: Not Applicable

Liu et al.2023 (9)	6	6	5	6	6	5	7	5	5	5	5	61	Good
Yuan et al.2023 (10)	5	5	5	6	6	5	7	5	5	5	5	59	Good
Deng et al.2022 (11)	3	3	5	5	5	5	6	6	5	5	5	54	Good
Liu et al.2022 (12)	5	5	3	5	5	5	6	3	3	3	5	45	Good
Chen et al.2022 (13)	3	3	5	5	5	5	6	6	5	5	5	54	Good
Zou et al.2023 (14)	6	6	5	6	6	5	5	6	5	5	5	60	Good
He et al.2024 (15)	5	5	5	5	5	5	5	6	5	5	5	56	Good
Yang et al.2024 (16)	5	5	5	5	5	5	6	6	5	5	5	57	Good
Cai et al.2024 (17)	3	5	5	5	5	5	6	6	5	5	5	55	Good
Zhang et al.2023 (18)	6	6	5	5	5	5	6	6	5	5	5	59	Good
Zhang et al.2024 (19)	6	6	5	5	5	5	6	6	5	5	5	59	Good
Xu et al.2023 (20)	5	5	5	5	5	5	5	5	5	5	5	55	Good
Li et al.2022 (21)	5	5	5	5	5	5	5	5	5	5	5	55	Good
Ding et al.2021 (22)	7	6	5	5	5	5	6	5	5	5	5	59	Good
Tan et al.2023 (23)	5	5	5	5	5	5	5	5	5	5	5	55	Good

Wang et al.2023 (24)	6	5	3	5	5	5	1	5	5	5	5	45	Good
Xiong et al.2022 (25)	6	6	5	5	5	5	5	5	5	5	5	57	Good

Supplementary Table 3. Risk of bias assessment of included studies evaluating genetically predicted alcohol consumption in relation to cardiovascular outcomes using Q-genie Tool

Quality Indicator[a] / **References**	***Rationale for study***	***Selection and definition of outcome of interest***	***Selection and comparability of comparison groups***	***Technical classification of the exposure***	***Non-technical classification of the exposure***	***Other sources of bias***	***Sample size and power***	***A priori planning of analyses***	***Statistical methods and control for confounding***	***Testing of assumptions and inferences for genetic analyses***	***Appropriateness of inferences drawn from results***	***Total score***[b]	***Overall rating***[c]
Van Oort et al,2020 (26)	5	1	3	3	1	5	7	5	7	5	5	47	Good
Zhao et al,2019 (27)	5	5	3	5	3	3	3	7	7	3	5	49	Good
Holmes et al,2014 (28)	5	5	3	5	3	3	5	3	7	3	5	47	Good
Millwood et al,2019 (29)	5	3	3	5	1	5	7	7	5	3	5	49	Good
Larsson et al,2019 (30)	5	3	3	3	3	5	5	3	5	3	5	46	Good
Jiang et al,2020 (31)	5	5	3	5	3	5	7	5	5	5	5	53	Good
Van Oort et al, 2020 (32)	5	1	3	3	1	5	7	5	7	5	5	48	Good
Christensen et al,2018 (33)	5	7	3	5	1	1	5	3	3	1	5	39	Moderate

[a] Scores range from 1 (poor) to 7 (excellent)

[b] Higher scores indicate higher overall quality

[c] Overall rating of poor, moderate or good

For studies with control groups: Scores ≤35 indicate poor quality studies, >35 and ≤45 indicate studies of moderate quality, and >45 indicate good quality studies.

For studies without control groups: Scores ≤32 indicate poor quality studies, >32 and ≤40 indicate studies of moderate quality, and >40 indicate good quality studies.

NA: Not Applicable

Cho et al,2015 (34)	5	5	3	5	3	5	1	5	5	3	5	45	Good
Rosoff et al,2020 **(35)**	5	5	3	3	1	5	7	5	5	3	5	45	Good
Au Yeung et al,2013 (36)	5	3	3	5	3	5	3	5	5	3	5	45	Good
Biddinger et al.2022 (37)	5	5	6	7	6	5	7	7	5	7	7	67	Good
Georgiou e al.2023 (38)	5	5	5	5	5	5	7	3	5	5	5	55	Good
Lu et al.2021 (39)	5	5	5	3	3	5	7	3	5	5	5	51	Good
Yang et al.2022 (40)	5	5	3	5	3	5	3	5	5	5	5	49	Good
Harshfield et al.2021 (41)	5	5	5	5	3	3	7	5	5	5	5	53	Good
Yang et al.2023 (42)	5	5	3	5	3	5	3	5	5	5	5	49	Good
Chunyu Hu et al.2022 (43)	5	5	5	5	5	5	7	5	5	5	5	57	Good
Jia et al.2022 (44)	5	5	5	5	5	5	7	7	5	5	5	54	Good
Lankester et al.2021 (45)	5	5	5	5	5	5	7	7	5	5	5	54	Good
Larsson et al.2024 (46)	7	6	7	7	5	5	6	6	7	5	7	63	Good
Hisamtsu et al.2024 (47)	3	3	3	5	5	5	5	5	5	5	5	49	Good
Tian et al.2022 (48)	6	5	5	5	5	5	6	6	6	5	6	60	Good

Supplementary Table 4. Risk of bias assessment of included studies evaluating genetically predicted alcohol consumption in relation to brain diseases using Q-genie Tool

Quality indicator[a] / References	Rationale for study	Selection and definition of outcome of interest	Selection and comparability of comparison groups	Technical classification of the exposure	Non-technical classification of the exposure	Other sources of bias	Sample size and power	A priori planning of analyses	Statistical methods and control for confounding	Testing of assumptions and inferences for genetic analyses	Appropriateness of inferences drawn from results	Total score[b]	Overall rating[c]
Almeida et al.2014 (49)	5	7	3	5	3	7	1	3	3	1	5	43	Moderate
Kumari et al.2014 (50)	7	5	3	7	3	1	1	5	3	1	5	41	Moderate
Ritchie et al.2013 (51)	5	1	3	7	3	5	1	5	1	7	5	43	Moderate
Mahedy et al.2020 (52)	5	7	3	1	3	1	5	3	5	1	5	44	Moderate
Andrews et al.2019 (53)	5	5	3	5	1	5	7	5	5	5	5	51	Good
Au Yeung et al.2011 (54)	7	5	3	3	3	1	3	5	5	3	5	43	Moderate
Yu et al.2020 (55)	7	5	3	5	3	5	7	7	5	5	5	57	Good
Domínguez-Baleón et al.2022 (56)	7	5	3	5	5	3	5	7	7	5	5	57	Good
Heilbron et al.2021 (57)	7	5	5	5	5	5	7	7	7	5	5	63	Good
Zhang et al.2022 (58)	3	6	6	6	5	5	6	6	7	5	7	62	Good
Yuan et al.2021 (59)	6	6	5	6	5	5	6	6	6	6	6	63	Good
Yang et al.2023 (42)	3	3	3	5	5	5	6	5	5	5	5	49	Good
Domenighetti et al.2022 (60)	6	5	5	5	5	5	5	5	7	5	7	60	Good
Meng et al.2024 (61)	6	5	5	5	5	5	6	6	6	5	6	60	Good
Li et al.2024 (55)	6	5	5	5	5	5	6	6	6	5	6	60	Good
Wang et al.2022 (62)	6	5	5	5	5	5	5	5	5	5	6	57	Good

[a] Scores range from 1 (poor) to 7 (excellent)

[b] Higher scores indicate higher overall quality

[c] Overall rating of poor, moderate or good

For studies with control groups: Scores ≤35 indicate poor quality studies, >35 and ≤45 indicate studies of moderate quality, and >45 indicate good quality studies.

For studies without control groups: Scores ≤32 indicate poor quality studies, >32 and ≤40 indicate studies of moderate quality, and >40 indicate good quality studies.

NA: Not Applicable

Larsson et al. 2017(63)	6	5	5	5	5	5	5	5	5	5	5	56	Good
Grover et al.2019 (64)	6	5	5	5	5	5	5	5	5	5	5	56	Good

Supplementary Table 5. Risk of bias assessment of included studies evaluating genetically predicted alcohol consumption in relation to liver diseases using Q-genie Tool

Quality indicator [a] / ***References***	***Rationale for study***	***Selection and definition of outcome of interest***	***Selection and comparability of comparison groups***	***Technical classification of the exposure***	***Non-technical classification of the exposure***	***Other sources of bias***	***Sample size and power***	***A priori planning of analyses***	***Statistical methods and control for confounding***	***Testing of assumptions and inferences for genetic analyses***	***Appropriateness of inferences drawn from results***	***Total score*** [b]	***Overall rating*** [c]
Lawlor et al.2014 (65)	7	5	3	5	5	3	5	7	5	5	5	55	Good
Carter et al.2019 (66)	5	3	3	5	7	5	7	7	5	3	5	55	Good
Sookoian et al ,2016 (67)	7	7	5	5	3	3	3	3	3	5	5	49	Good
Yuan et al.2022 (68)	6	6	6	7	6	5	7	6	7	6	6	68	Good
Im et al.2023 (69)	6	6	6	5	5	5	7	6	6	5	6	63	Good

[a] Scores range from 1 (poor) to 7 (excellent)
[b] Higher scores indicate higher overall quality
[c] Overall rating of poor, moderate or good
<u>For studies with control groups</u>: Scores ≤35 indicate poor quality studies, >35 and ≤45 indicate studies of moderate quality, and >45 indicate good quality studies.
<u>For studies without control groups</u>: Scores ≤32 indicate poor quality studies, >32 and ≤40 indicate studies of moderate quality, and >40 indicate good quality studies.
NA: Not Applicable

Supplementary Table 6. Methodological quality assessment through testing for assumptions: Genetically predicted alcohol consumption and cancer outcomes

Study	***study design***	***F stat***	***r2***	***SNPs used***	***Methods used***	***Assumption 1***	***Assumption 2***	***Assumption 3***	***Non-linearity***
Gormley et al,2020 (1)	Two Sample	74.7 (GSCAN)<br>49 (Uk Biobank)	r2 < 0.07, with the exception of rs10236149 and rs6962772 which had an r2 of 0.743	60 SNPs	IVW	Good	Good	Good	Poor
Zhu et al,2020 (2)	Two sample	-	-	99 SNPs for drinks / week, 9 SNPs for AUD ,13 SNPs for AUDIT-C	IVW	Good	Good	Good	Poor
Larsson et al,2020(3)	Two Sample	19–29	-	29 SNPs	IVW	Good	Good	Good	Poor
Brunner et al,2017 (4)	One Sample	-	0,99	68 SNPs , ALDH1A2 rs1441817, ALDH1B1 rs10973794	Logistic regression , cox proportional hazards regression , meta-regression	Good	Good	Good	Poor
Ong et al,2020 (5)	Two Sample	50	0.0094	34 SNPs , ADH1B rs1229984	Cox-regression , IVW	Good	Good	Good	Poor
Chen et al.2021(6)	Two Sample	-	0.003	14 SNPs	IVW<br>MR-Egger<br>weighted median<br>MR-PRESSO	Good	Good	Good	Poor
Zhou et al.2022 (7)	Two sample	-	-	Drinks per Week: 84 SNPs<br>Alcohol Use Disorder (AUD): 18 SNPs<br>Problematic Alcohol Use (PAU): 26 SNPs	IVW<br>MR-Egger<br>weighted median<br>MR-PRESSO	Good	Good	Good	Poor
Zhou et al.2022 (8)	Two sample	-	-	Drinks per Week: 99 SNPs.<br>Alcohol Use Disorder (AUD): 30 SNPs.<br>Problematic Alcohol Use (PAU): 42 SNPs	IVW<br>MR-Egger<br>weighted median<br>MR-PRESSO	Good	Good	Good	Poor
**Liu et al.2023** (9)	Two sample	Range from 41 to 642	-	37 SNPs	IVW<br>MR-Egger<br>weighted median<br>MR-PRESSO	Good	Good	Good	Poor
**Yuan et al.2023** (10)	Two sample	-	-	99 SNPs	IVW<br>MR-Egger<br>weighted median<br>MR-PRESSO	Good	Good	Good	Poor

**Deng et al.2022 (11)**	One sample	-	-	ADH1B rs1229984<br>ALDH2 rs671	IVW<br>MR-Egger<br>weighted median<br>MR-PRESSO	Good	Good	Good	Poor
**Liu et al.2022 (12)**	One sample	-	-	6 SNPs	Trend Test<br>Fractional Polynomial Test<br>Meta-Regression	Good	Good	Poor	Good
**Chen et al.2022 (13)**	Two sample	33.6	-	169 SNPs	IVW<br>MR-Egger<br>weighted median<br>MR-PRESSO	Good	Good	Good	Poor
**Zou et al.2023 (14)**	Two sample	59.85	0.78	44 SNPs	IVW<br>MR-Egger<br>weighted median<br>MR-PRESSO	Good	Good	Good	Poor
**He et al.2024 (15)**	Two sample	-	-	184 SNPs	IVW<br>MR-Egger<br>weighted median<br>MR-PRESSO	Good	Good	Good	Poor
**Yang et al.2024 (16)**	Two sample	-	-	57 SNPs	IVW<br>MR-Egger<br>weighted median<br>MR-PRESSO	Good	Good	Good	Poor
**Cai et al.2024 (17)**	Two sample	-	-	5 SNPs	IVW<br>MR-Egger<br>weighted median<br>MR-PRESSO	Good	Good	Good	Poor
**Zhang et al.2023 (18)**	Two sample	-	-	6 SNPs	IVW<br>MR-Egger<br>weighted median<br>MR-PRESSO	Good	Good	Good	Poor
**Zhang et al.2024 (20)**	Two sample	68.74	-	76 SNPs	IVW<br>MR-Egger<br>weighted median<br>MR-PRESSO	Good	Good	Good	Poor
**Xu et al.2023 (20)**	Two sample	176	0.014	31 SNPs	IVW<br>MR-Egger<br>weighted median<br>MR-PRESSO	Good	Good	Good	Poor
**Li et al.2022 (21)**	Two sample	Ever versus never drinker :72.53<br>Drinks per week : 80.54 ; 22500.00	-	6 SNPs :Ever versus never drinker<br>2 SNPs :Drinks per week	IVW<br>MR-Egger<br>weighted median<br>MR-PRESSO	Good	Good	Good	Poor
**Ding et al.2021 (22)**	Two sample	160.44	-	ADH1B rs1229984	IVW<br>MR-Egger<br>weighted median<br>MR-PRESSO<br>Maximum Likelihood Method<br>CAUSE Method	Good	Good	Poor	Poor
**Tan et al.2023 (23)**	Two sample	-	-	179 SNPs (European)<br>66 SNPs (Asian)	IVW<br>MR-Egger<br>weighted median<br>MR-PRESSO<br>MR-RAPS<br>Radial MR<br>Summary Effect Estimates	Good	Good	Good	Poor

					(CAUSE)				
**Wang et al.2023 (24)**	Two sample	-	-	41 SNPs	IVW<br>MR-Egger<br>weighted median<br>MR-PRESSO	Good	Good	Good	Poor
**Xiong et al.2022 (25)**	Two sample	>82.93	-	39 SNPs	IVW<br>MR-Egger<br>weighted median<br>MR-PRESSO<br>Penalised weighted median<br>IVW radial	Good	Good	Good	Poor

Supplementary Table 7. Methodological quality assessment through testing for assumptions: Genetically predicted alcohol consumption and cardiovascular outcomes

Study	***Study design***	***F stat***	***r2***	***SNPs used***	***Methods used***	***Assumption 1***	***Assumption 2***	***Assumption 3***	***Non-linearity***
van Oort et al,2020 **(**26)	Two Sample	-	-	91 SNPs	IVW	Good	Good	Good	Poor
Zhao et al,2019 **(27)**	One Sample	-	-	ALDH2 rs671	2SLS	Poor	Good	Good	Poor
Holmes et al,2014 **(**28)	One Sample	-	-	ADH1B rs1229984	Meta-regression	Good	Good	Good	Good
Millwood et al,2019 **(**29)	One Sample	-	-	ALDH2 rs671 ; ADH1B rs1229984	IVW	Poor	Good	Good	Good
Larsson et al,2020 (30)	Two Sample	19–29	-	94 SNPs ; ADH1B rs1229984	IVW	Good	Good	Good	Poor
Jiang et al,2020 (31)	Two Sample	>10	> 0.001	43 SNPs	IVW	Good	Good	Good	Poor
Van Oort et al,2020 **(**32)	Two Sample	-	-	91 SNPs	IVW	Good	Good	Good	Poor
Christensen et al,2018 **(**33)	One Sample	22	-	ADH1B rs1229984 ; ADH1C rs698	Linear regression	Good	Good	Poor	Poor
Cho et al,2015 (34)	One Sample	262 (men)<br>38 (women	-	ALDH2 rs671	2SLS	Good	Good	Good	Poor

Rosoff et al,2020 (35)	Two sample	-	> 0.001	71 SNPs	Weighted linear regression	Good	Good	Good	Poor
Au Yeung et al,2013 (36)	One Sample	74.6	0.03	ALDH2 rs671	2SLS	Good	Good	Poor	Poor
Biddinger et al.2022 (37)	Two sample	780	-	AUD : 9 SNPs<br>AUDIT-C :13 SNPs	IVW<br>MR-Egger<br>weighted median<br>MR-PRESSO	Good	Good	Good	Good
Georgiou et al.2023 (38)	Two sample	-	-	73 SNPs	IVW<br>MR-Egger<br>weighted median<br>MR-PRESSO	Good	Good	Good	Poor
Lu et al.2021 (39)	Two sample	-	> 0.001	6 SNPs	IVW,<br>MR-RAPS<br>MR-PRESSO<br>MVMR	Good	Good	Good	Poor
Yang et al.2022 (40)	One sample	864.6	0.195	ALDH2 rs671	2SLS<br>linear regression	Good	Good	Good	Poor
Harshfield et al.2021 (41)	Two sample	-	-	35 SNPs	IVW<br>MR-Egger<br>weighted median<br>MR-PRESSO	Good	Good	Good	Poor
Yang et al.2023 (42)	Two sample	-	-	84 SNPs	IVW<br>MR-Egger<br>weighted median<br>MR-PRESSO	Good	Good	Good	Poor
Hu et al.2022 (43)	One sample	-	-	ALDH2 rs671	Two-Stage Predictor Substitution (2SPS)<br>Cox Regression	Good	Good	Good	Good
Jia et al.2022 (44)	Two sample	64 (alcohol consumption)<br>41 (alcohol dependance)	-	89 SNPs : Alcohol intake<br>2 SNPs : Alcohol dependance	IVW<br>MR-Egger<br>weighted median<br>MR-PRESSO	Good	Good	Good	Poor
Lankester et al.2021 (45)	Two sample	-	-	ADH1B rs1229984<br>+<br>24 SNPs	IVW<br>2SLS	Good	Good	Good	Poor
Larsson et al.2024 (46)	Two sample	66	-	84 SNPs	IVW<br>MR-Egger<br>weighted median<br>MR-PRESSO	Good	Good	Good	Poor
Hisamtsu et al.2024 (47)	One sample	-	-	ALDH2 rs671	2SLS	Good	Good	Good	Poor
Tian et al.2022 (48)	Two sample	-	0.006326	53 SNPs	IVW<br>MR-Egger<br>weighted median<br>MR-PRESSO	Good	Good	Good	Poor

Supplementary Table 8. Methodological quality assessment through testing for assumptions: Genetically predicted alcohol and brain diseases

Study	*study design*	*F stat*	*r2*	*SNPs used*	*Methods used*	*Assumption 1*	*Assumption 2*	*Assumption 3*	*Non-linearity*
Almeida et al,2014 (49)	One Sample	-	-	ADH1B rs1229984	Regression analysis	Good	Poor	Poor	Poor
Kumari et al,2014 (50)	One Sample	47	0,1	ADH1B rs1229984	Linear regression<br>2SLS	Good	Good	Good	Poor
Ritchie et al,2013 (51)	One Sample	72.83	0,48	4 SNPs : ADH1A rs2866151 rs975833, ADH7 rs284779 ; ADH1B rs4147536	Linear regression	Good	Moderate	Moderate	Poor
Mahedy et al,2020 (52)	Two Sample	-	-	87 SNPs	Linear regression<br>IVW	Good	Good	Poor	Poor
Andrews et al,2019 (53)	Two Sample	F=70.8 for alcohol consumption, F=24,6 for alcohol dependence, F=59,3 for AUDIT	-	44 SNPs : Alcohol consumption<br>22 SNPs : Alcohol dependance<br>11 SNPs : AUDIT	IVW	Good	Good	Good	Poor
Au Yeung et al,2011 (54)	One Sample	72	-	ALDH2 rs671	2SLS<br>Linear regression	Good	Good	Good	Poor
Yu et al,2020 (55)	One Sample	>10	-	46 SNPs	IVW	Good	Good	Good	Poor
Domínguez Baleón et al.2022 (56)	Two sample	147.00378	0.00513	33 SNPs	IVW<br>GCTA-GSMR<br>MR-Egger<br>MR-PRESSO	Good	Good	Good	Poor
Heilbron et al.2021 (57)	Two sample	F =49.5 (cohort 1)<br>F = 48.3 (cohort 2)	-	129 SNPs (cohort 1)<br>124 SNPs (cohort 2)	IVW<br>MR-Egger<br>weighted median	Good	Good	Good	Good

Zhang et al.2022 (58)	Two sample	-	-	84 SNPs	IVW<br>MR-Egger<br>weighted median<br>MR-PRESSO	Good	Good	Good	Poor
Yuan et al.2021 (59)	Two sample	103		79 SNPs	Cochrane Q Statistic<br>IVW<br>MR-Egger<br>weighted median<br>MR-PRESSO	Good	Good	Good	Poor
Domenighetti et al.2022 (60)	Two sample	-	-	ADH1B rs1229984<br>64 SNPs	IVW<br>MR-Egger<br>weighted median<br>MR-PRESSO	Good	Good	Good	Poor
Meng et al.2024 (61)	Two sample	-	-	30 SNPs : Alcoholic drinks per week<br>89 SNPs : Alcohol intake frequency	IVW<br>MR-Egger<br>weighted median<br>MR-PRESSO	Good	Good	Good	Poor
Li et al.2024 (55)	Two sample	-	-	89 SNPs : Alcohol intake frequency<br>30 SNPs : Alcoholic drinks per week	IVW<br>MR-Egger<br>weighted median<br>MR-PRESSO	Good	Good	Good	Poor
Wang et al.2022 (62)	Two sample	-	-	99 SNPs : Drinks per week<br>46 SNPs : Grams per day	IVW<br>MR-Egger<br>weighted median<br>MR-PRESSO	Good	Good	Good	Poor
Larsson et al. 2017(63)	One sample	-	-	2 SNPs	IVW<br>MR-Egger<br>weighted median<br>leave one out analysis	Good	Good	Good	Poor
Grover et al.2019 (64)	Two sample	4,342,4	-	70 SNPs	IVW<br>MR-Egger<br>weighted median<br>leave one out analysis	Good	Good	Good	Poor

Supplementary Table 9. Methodological quality assessment through testing for assumptions: Genetically predicted alcohol and liver outcomes

Study	*study design*	*F stat*	*r2*	*SNPs used*	*Methods used*	*Assumption 1*	*Assumption 2*	*Assumption 3*	*Non-linearity*
Lawlor et al,2014 (65)	One Sample	21	0.006	ADH1B rs1229984<br>ADH1C rs698	Multivariable linear regression	Good	Good	Poor	Poor
Carter et al,2019 (66)	One Sample	117.4	0.0013	ADH1B rs1229984	Multivariable linear regression, logistic regression<br>2SLS	Good	Good	Poor	Poor
Sookoian et al ,2016 **(**67)	One Sample	-	-	ADH1B rs1229984	Logistic regression	Good	Good	Poor	Poor
Yuan et al.2022 (68)	Two Sample	29	-	74 SNPs (Discovery stage) ; 83 SNPs (replication stage)	IVW, Weighted Median, MR-PRESSO, MR-Egger	Good	Moderate	Good	Poor

Im et al.2023 (69)	one Sample	-	-	ALDH2-rs671 ; ADH1B-rs1229984	Cox Regression, Meta-Regression, 2SLS,IVW	Good	Good	Good	Poor

Abbreviations: GWAS = genome-wide association study; GCTA-GSMR = genome-wide complex trait analysis-generalized summary mendelian randomization ; IV = instrumental variable; IVW = inverse-variance weighted; MR = Mendelian randomization; MR-PRESSO = Mendelian Randomization Pleiotropy RESidual Sum and Outlier ; SNP = single nucleotide polymorphism; 2SLS = 2 stage least squares; RR = risk ratio. First assumption: the genetic variant is associated with alcohol consumption. Second assumption: the genetic variant is not associated with any confounder of the alcohol consumption-outcome association. Third assumption: the genetic variant does not affect the outcome, except possibly via its association with alcohol consumption.

Supplementary Table 10. Characteristics and results of included mendelian randomization studies investigating genetically predicted alcohol consumption and cancer outcomes

Study	*Sample size*	*Ancestry*	*Sex*	*Mean age*	*IV*	*Exposure dataset*	*outcomes*	*Outcome dataset*	*Results Association with alcohol consumption*
Gormley et al,2020 (1)	6034 cases ; 6585 controls	Mixed (European and American)	-	-	60 SNPs	GSCAN UKB	Oral and oropharyngeal cancer	GAME-ON	OR = 2.1 (1.1- 3.8)
Zhu et al,2020 (2)	Breast cancer cases n= 122,977; ovarian cancer cases n= 25,509	European	-	-	99 SNPs : drinks/week<br>9 SNPs : AUD<br>13 SNPs : AUDIT-C	GWAS	Breast and ovarian cancer	GWAS	Breast cancer :OR $_{\text{drinks/week}}$ = 1.01 (0.85 –1.21), p =0.89; OR$_{\text{AUD}}$ = 1.04 (0.89–1.21), p=0.62; OR $_{\text{AUDIT-C}}$ =1.07 (0.90–1.28), p =0.44<br>Ovarian cancer : OR $_{\text{drinks/week}}$ = 0.83 (0.63–1.10), p =0.19; OR $_{\text{AUD}}$ = 0.92 (0.83–1.01), p = 0.08; OR $_{\text{AUDIT-C}}$ = 0.83 (0.71–0.97), p =0.02

Study	Sample size	Ancestry	Sex	Mean age	IV	Exposure dataset	outcomes	Outcome dataset	Results Association with alcohol consumption
Larsson et al,2020 (3)	75,037 cases	European	-	-	29 SNPs	GWAS	Overall cancer, Testis, Lung , Esophagus, Head/neck, Cervix, Colorectum, Ovary, Pancreas, Multiple myeloma, Lung, Breast ,Prostate , Prostate , Kidney, Stomach, Bladder, Uterus, Brain, Melanoma, Non-Hodgkin lymphoma, Leukemia	UK Biobank , ILCCO ,BCAC, OCAC, PRACTICAL	Overall cancer :OR =0.95 (0.84 - 1.07); p =0.376<br>Testis: OR = 2.15 (0.81 - 5.71), p =0.125<br>Lung (ILCCO): OR = 1.94 (1.41 - 2.68), p =4.68e-05<br>Esophagus: OR = 1.88 (0.76 - 4.66), p =0.171<br>Head/neck: OR = 1.75 (0.83 - 3.72), p =0.144<br>Cervix: OR = 1.33 (0.73 - 2.42), p =0.351<br>Colorectum: OR = 1.31 (0.84 - 2.04), p =0.235<br>Ovary: OR = 1.23 (0.62 - 2.47), p =0.554<br>Pancreas: OR = 1.16 (0.55, 2.43), p =0.703<br>Multiple myeloma: OR = 1.15 (0.41 - 3.21), p =0.795<br>Lung (UK Biobank): OR = 1.12 (0.65 - 1.93), p = 0.686<br>Breast (UK Biobank): OR = 1.10 (0.87 - 1.40), p = 0.423<br>Breast (BCAC): OR = 0.99 (0.83 - 1.18), p = 0.922<br>Prostate (PRACTICAL): OR = 0.96 (0.74 - 1.24), p = 0.753<br>Ovary (OCAC): OR = 0.91 (0.73 - 1.15), p = 0.438<br>Prostate (UK Biobank): OR = 0.91 (0.62 - 1.34), p = 0.630<br>Kidney: OR = 0.90 (0.44 - 1.86), p =0.786<br>Stomach: OR = 0.88 (0.33 - 2.30), p =0.788<br>Bladder: OR = 0.85 (0.49 - 1.44), p =0.539<br>Uterus: OR = 0.84 (0.45 - 1.58), p =0.591<br>Brain: OR = 0.69 (0.26 - 1.87), p =0.467<br>Melanoma: OR = 0.68 (0.46 - 1.02), p =0.059<br>Non-Hodgkin lymphoma: OR = 0.66 (0.37 - 1.18), p =0.157<br>Leukaemia: OR = 0.51 (0.25 - 1.02), p =0.056
Brunner et al,2019 (4)	23,868 cases ; 23,091 controls	European	Only Men	Average from 58,45 to 72,08	68 SNPs + ALDH1A2 rs1441817; ALDH1B1 rs10973794	PRACTICAL Consortium	Prostate cancer incidence and mortality	PRACTICAL Consortium	prostate cancer mortality/ALDH1A2 rs1441817 HR_{fixed} = 0.78 (0.66 - 0.91), p = 0.002<br>prostate cancer mortality/ALDH1A2 rs12910509 HR_{fixed} = 0.76 (0.64 - 0.91), p = 0.003<br>prostate cancer mortality/ALDH1A2 rs8041922 HR_{fixed} = 0.76 (0.64 - 0.91), p = 0.002<br>prostate cancer mortality/ALDH1A2 rs10973794 HR_{fixed} = 1.43 (1.14 - 1.79), p = 0.002
Ong et al,2021 (5)	population case controls from BCAC+OCAC	European	Only Women	-	34 SNPs + ADH1B rs1229984	UKB	Breast and ovarian cancer	BCAC ; OCAC	Breast cancer : $OR_{drink/day}$ = 1.03 (0.93 - 1.14)<br>EOC : $OR_{drink/day}$ = 0.89 (0.73 - 1.08)
Chen et al,2021 (6)	11,348	European	-	-	14 SNPs	Data from Neale Lab	Lung cancer ; lung squamous cell cancer	ILCCO ; UK Biobank	Lung cancer OR = 0.175 (0.045 - 0.682), p =0.012<br>Lung squamous cell cancer OR = 0.075 (0.013 - 0.429),p =0.004
Im et al.2023 (69)	2452	Asian	41% men	52	ALDH2-rs671 ; ADH1B-rs1229984	CKB	Esophageal cancer<br>Colon cancer<br>Liver cancer<br>Stomach cancer	CKB	**Esophageal cancer** HR per 280 g/week M 1.47 (1.09, 1.99);HR per 280 g/week W = 1.58 (0.85, 2.93)<br>**Colon cancer** HR per 280 g/week M = 1.38 (0.90 - 2.11); HR per 280 g/week W = 1.23 (0.89 - 1.72)

Study	*Sample size*	*Ancestry*	*Sex*	*Mean age*	*IV*	*Exposure dataset*	*outcomes*	*Outcome dataset*	*Results Association with alcohol consumption*
							oral cavity & pharynx cancer<br>Rectal cancer<br>Lung cancer<br>Larynx cancer		**Liver cancer** HR per 280 g/week M = 1.23 (0.93 - 1.62); HR per 280 g/week W = 0.85 (0.61 - 1.20)<br>**Stomach cancer** HR per 280 g/week M = 1.16 (0.89 - 1.50) ; HR per 280 g/week W = 0.85 (0.61 - 1.20)<br>**Lip,Oral cavity and pharynx cancer** HR per 280 g/week M = 1.02 (0.59 - 1.75) ; HR per 280 g/week W = 0.95 (0.45 - 1.99)<br>**Rectal cancer** HR per 280 g/week M = 1.01 (0.71 - 1.46) ; HR per 280 g/week W = 0.79 (0.55 - 1.14)<br>**Lung cancer** HR per 280 g/week M = 0.81 (0.67 - 0.98); HR per 280 g/week W = 0.85 (0.68 - 1.06)<br>**Larynx cancer** HR per 280 g/week M = 0.58 (0.14 - 2.34); HR per 280 g/week W = 0.02 (0.00 - 4.45)
Zhou et al.2022 (7)	20,049 cases and 22,661 controls	European	-	-	84 SNPs : Drinks per Week<br>18 SNPs : AUD<br>26 SNPs : Problematic Alcohol Use (PAU)	GSCAN	Colorectal Cancer	Summary-level data from 12 CRC GWASs	**Drinks per Week:**<br>OR = 1.79 (1.23 - 2.61),p = 0.003<br>**Alcohol Use Disorder (AUD):**<br>OR = 1.33 (0.95 - 1.85),p = 0.093<br>**Problematic Alcohol Use (PAU):**<br>OR = 1.53 (1.02 - 2.29),p = 0.040
Zhou et al.2022 (8)	133,384 cases and 113,789 controls	European	-	-	84 SNPs : Drinks per Week<br>19 SNPs : AUD<br>26 SNPs : Problematic Alcohol Use (PAU)	GSCAN	Breast Cancer	BCAC	**Drinks per Week:**<br>OR = 1.01 (0.84 - 1.23),p = 0.883<br>**Alcohol Use Disorder (AUD):**<br>OR = 1.05 (0.80 - 1.37),p = 0.721<br>**Problematic Alcohol Use (PAU):**<br>OR = 1.03 (0.82 - 1.30), p = 0.781<br>**Conditioning on alcohol consumption PAU**<br>OR_{PAU} = 1.76 (1.04 - 2.99) ,p=0.036
Liu et al.2023 (9)	25,509 cases; 40,941 controls	European	Women	-	37 SNPs	33 GWAS and a genome-wide meta-analysis of 28 studies	Risk of ovarian cancer and prognosis	OCAC	OR = 0.74 (0.52 -1.04);p =0.081

Study	Sample size	Ancestry	Sex	Mean age	IV	Exposure dataset	outcomes	Outcome dataset	Results Association with alcohol consumption
Yuan et al.2023 (10)	**Esophageal cancer**:1130 cases;702,116 controls **Gastric cancer** : 1608 cases; 701,472 controls **Colorectal cancer** :9519 cases;686,953 controls **Liver cancer:** 714 cases;702,008 controls **Pancreatic cancer :** 1643 cases;701.472 controls	European	-	-	99 SNPs	UKB	Esophageal cancer Gastric cancer Colorectal cancer Liver cancer Pancreatic cancer	UK Biobank, FinnGen Study, International Inflammatory Bowel Disease Genetics Consortium (IIBDGC), Genetic Epidemiology Research on Aging (GERA)	**Esophageal cancer** :UVMR OR = 2.86 (1.18 - 6.91); p =0.020 MVMR adjusted for smoking OR = 1.28 (0.59 - 2.82);p =0.533 **Gastric cancer :** UVMR OR = 1.57 (0.75 - 3.30);p = 0.233 MVMR adjusted for smoking OR = 1.59 (0.79 - 3.21);p =0.194 **Colorectal cancer :** UVMR OR = 1.09 (0.76 - 1.55);p =0.649 MVMR adjusted for smoking OR = 1.28 (0.95 - 1.72);p =0.098 **Liver cancer** UVMR OR = 1.16 (0.43 - 3.11);p =0.775 MVMR adjusted for smoking OR = 0.76 (0.29 - 2.02);p =0.585 **Pancreatic cancer** UVMR OR = 0.63 (0.32 - 1.26),p =0.193 MVMR adjusted for smoking OR = 0.79 (0.40 - 1.56);p =0.496
Deng et al.2022 (11)	HCC Cases: 1,866 Controls: 195,745	Asian	Cases: 1384 men and 482 women Controls : 97,655 men and 98,090 women	Cases: 68.0 ± 8.4 years Controls: 61.6 ± 13.9 years	Alcohol consumption:ADH1B rs1229984+ ALDH2 rs671 ever/never drinkers :5 SNPs	BioBank Japan (BBJ)	Hepatocellular Carcinoma	BioBank Japan (BBJ)	OR $_{\text{alcohol consumption}}$ = 1.57 (1.32 - 1.86), p < 0.001 OR $_{\text{ever/never drinker}}$ = 1.11 (1.05–1.18) ;p < 0.001
Liu et al.2022 (12)	329,164 participants	European	< 60 years: 56.7% ≥ 60 years: 43.3%	56.7 [8.0] years	6 SNPs (rs11940694, rs1229984, rs1302808, rs55872084, rs7187575, rs676388)	UK Biobank	Hepatocellular carcinoma	UK Biobank	p< 0.0001 in the trend test
Chen et al.2022 (13)	Breast Cancer Cases: 122,977 Controls: 123,082	European	Only women	-	169 SNPs	UK Biobank	Breast cancer	BCAC	OR = 1.01 (0.89 – 1.16),p = 0.829
Zou et al.2023 (14)	503 cases and 259,583 non-cancer controls	European	-	-	44 SNPs for alcohol intake frequency	UK Biobank	Esophageal cancer	FinnGen consortium	OR = 0.206 (0.545-2.668) ;p =0.644

Study	Sample size	Ancestry	Sex	Mean age	IV	Exposure dataset	outcomes	Outcome dataset	Results Association with alcohol consumption
He et al.2024 (15)	5,567 colorectal cancer cases ;372,016 controls	European	-	-	27 SNPs: Alcohol drinker status (current)<br>23 SNPs: Alcohol drinker status (Never)<br>20 SNPs: Alcohol drinker status (Previous)<br>12 SNPs: Alcohol (Female)<br>9 SNPs: Alcohol (Male)<br>184 SNPs: Alcohol intake frequency<br>20 SNPs: White wine<br>16 SNPs: Fortified wine<br>14 SNPs: Red wine	IEU OPEN GWAS PROJECT	Colorectal cancer	UK Biobank	**Alcohol drinker status (current)** :OR = 1.012 (0.974 -1.051),p =0.556<br>**Alcohol drinker status (Never) :**OR = 1.010 (0.957-1.067),p =0.715<br>**Alcohol drinker status (Previous)** :OR = 1.001 (0.935 - 1.072),p =0.971<br>**alcohol (female):** OR = 1.004 (0.998 – 1.011),p =0.184)<br>**alcohol (male):** OR = 1.001 (0.993 – 1.008), p =0.870),<br>**alcohol intake frequency:** OR = 0.999 (0.997–1.001),p =0.154<br>**white wine:**<br>OR = 1.001 (0.997–1.005),p =0.708<br>**fortified wine :** OR = 0.997 (0.981–1.014),p =0.764<br>**red wine** :OR = 1 (0.997–1.002),p =0.893
Yang et al.2024 (16)	121,885: 12,906 cases ;108,979 controls	European	-	-	57 SNPs	GSCAN	Endometrial Cancer	Endometrial Cancer Association Consortium, the Endometrial Cancer Epidemiology Consortium, UK Biobank	**Overall Endometrial Cancer (IVW):** OR = 0.57 (0.41 – 0.79), p < 0.001<br>**Endometrioid Endometrial Cancer (IVW):** OR = 0.56 (0.38 – 0.83),p = 0.004<br>**Non-Endometrioid Endometrial Cancer (IVW):** OR = 1.36 (0.40 – 4.66),p = 0.626
Cai et al.2024 (17)	digestive system cancers 17,572 DSCs cases and 195,745 controls	Asian	-	-	5 SNPs	Matoba et al. study (2020)	Esophageal(EC), gastric (GC), colorectal (CRC), hepatocellular (HCC), biliary tract (BTC), pancreatic cancer (PC)	Biobank Japan	**HCC** OR = 1.11 (1.05–1.18)<br>**gastric cancer** OR = 0.95 (0.93–0.98)<br>**Esophageal cancer** OR = 2.24E4 (40.02-1.25E7), p =0.0019<br>**Colorectal cancer** OR =0.84 (0.23 - 3.07); p =0.7952<br>**BTC** OR = 0.98 (0.89 - 1.07),p =0.6363<br>**PC** OR = 1.01 (0.92 - 1.10),p =0.9119
Zhang et al.2023 (18)	15,591 cases and 22,141 controls	Mixed	-	-	4 SNPs	UK Biobank	esophageal squamous cell carcinoma (ESCC)	IEU Open GWAS	**ALDH2 rs671 /ESCC** OR = 0.60 (0.50 – 0.73)<br>**ADH1B /ESCC**(additive model (AA vs. GG)) OR =2.50 (1.70 –3.69)<br>**ADH1B /ESCC** (allelic model (A vs. G))<br>OR = 1.50 (1.21 – 1.87)<br>**ALDH2 rs674/ESCC** OR = 1.22 (0.71–2.12)<br>**ADH1B rs1042026** OR = 1.28 (0.52 – 3.14)<br>**5 SNPs/ESCC** OR = 0.99 (0.99 – 1.00)
Zhang et al.2024 (19)	309,174	European	-	-	76 SNPs	UK Biobank (30643251)	Liver cancer	FinnGen consortium	OR drinks/per week = 1.57 (0.57-5.03);p =0.339
Xu et al.2023 (20)	2,993 cases and 287,137 controls	European	-	-	31 SNPs	UK Biobank (30643251)	cutaneous melanoma	FinnGen consortium	OR drinks/per week = 2.23 (1.11- 4.47), p =0.02

Study	Sample size	Ancestry	Sex	Mean age	IV	Exposure dataset	outcomes	Outcome dataset	Results Association with alcohol consumption
Li et al.2022 (21)	6692 cases;27.178 controls	Asian	-	-	6 SNPs: Ever versus never drinkers<br>2 SNPs: Number of drinks per week	Matoba et al,2020	Colorectal Cancer	Tanikawa et al.2018	OR $_{\text{ever versus never drinker}}$ = 1.08 (1.05 –1.12), p = 1.51 × 10–5<br>OR $_{\text{drinks/per week}}$ = 1.39 (1.27–1.52),p = 5.29 × 10–13
Ding et al.2021 (22)	2,485 lung cancer cases ; 410,350 controls	European	-	-	ADH1B rs1229984	GSCAN	Lung cancer	UK Biobank ; Kaiser Permanente Genetic Epidemiology Research on Adult Health and Aging (GERA)	OR = 1.30 (0.39 - 4.35),p = 0.674
Tan et al.2023 (23)	6,563 GC cases and 195,745 controls	Mixed(Asian +European)	-	-	66 SNPs (East Asian populations)<br>179 SNPs (European population)	UK Biobank (Ukb-a-25)	Gastric cancer	GWAS datasets from the MRC Integrative Epidemiology Unit (IEU)	**66 SNPs/GC** OR = 1.01 (0.98 - 1.04),p =0.562<br>**179 SNPs/GC** OR = 1.05 (0.94 - 1.18),p =0.359
Wang et al.2023 (24)	1106 (Head and neck cancer)<br>989 (Thyroid cancer)	European	-	-	41 SNPs (34 SNPs for drinks per week+7 SNPs for overall alcohol consumption)	GSCAN,WFGC (within family GWAS consortium)	head and neck cancer<br>Thyroid cancer	UK Biobank	**34 SNPs / Head and neck cancer** OR = 1.003 (1.001–1.006);p = 0.014<br>**7 SNPs/ Head and neck cancer** OR =1.000 (0.999-1.002);p =0.537<br>**34 SNPs/ Thyroid cancer** OR =1.407 (0.461 - 4.294) ,p =0.549<br>**7 SNPs/Thyroid cancer** OR =1.188 (0.762 - 1.850) ;p =0.447
Xiong et al.2022 (25)	1,115 cases ;174,006 controls (Finnegen) ; 2,883 cases;417,955 controls (UKB)	European	-	-	39 SNPs	UK Biobank	Bladder cancer	FinnGen ;UK Biobank	OR = 0.69 (0.44 - 1.10),p = 0.1237

ADH1B: Alcohol Dehydrogenase 1B; ALDH2: Aldhyde Dehydrogenase 2; BCAC: Breast Cancer Association Consortium; CI: Confidence Interval; GWAS: Genome-wide Association Study; HR: Hazard Ratio; ILCCO: The International Lung Cancer Consortium; OCAC: Ovarian Cancer Association Consortium; OR: Odds Ratio; PRACTICAL: PRostate cancer AssoCiation group to Investigate Cancer-associated ALterations in the genome; SNP: Single Nucleotide Polymorphism;M: men ; W: women

Supplementary Table 11. Characteristics and results of included mendelian randomization studies investigating genetically predicted alcohol consumption and cardiovascular outcomes

Study	*Sample size*	*Ancestry*	*Sex*	*Mean age*	*IV*	*Exposure dataset*	*Outcome*	*Outcome dataset*	*Results Association with genetically predicted alcohol consumption*
Van Oort et al, 2020 (26)	47,309 cases , 930,014 controls	European	-	-	91 SNPs	GWAS ,GSCAN	HF	26 studies: Heart failure Molecular Epidemiology for therapeutic targets consortium	OR $_{drinks/week}$ = 1.11(0.85 - 1.46)
Zhao et al,2019 (27)	2349	Asian	54.1% women	59±11	ALDH2 rs671	-	HT	-	OR =1.19 (1.04 - 1.36),p =0.011
Holmes et al,2014 (28)	261,991	European	52% men	58	ADH1B rs1229984	56 studies	CHD, stroke	56 studies	**CHD** OR (GG versus AA or AG) = 1.11 (1.04 - 1.19)<br>**Stroke** OR (GG versus AA or AG) = 1.02 (0.93 - 1.11)
Millwood et al,2019 (29)	161 498	Asian	41% Male	52±11	ALDH2 rs671 , ADH1B rs1229984	China kadoorie biobank	AMI , CHD,stroke	-	**AMI** RR $_{per\ 280g/week}$ M = 0.96 (0.78 - 1.18) ; RR $_{per\ 280g/week}$ W = 0.94 (0.74 - 1.20)<br>**CHD** RR $_{per\ 280g/week}$ M = 1.05 (0.94 - 1.17) ; RR $_{per\ 280g/week}$ W = 1.02 (0.93 - 1.12)<br>**Stroke** RR $_{per\ 280g/week}$ M = 1.38 (1.26 - 1.51) ; RR $_{per\ 280g/week}$ W = 0.98 (0.88 - 1.09)
Larsson et al,2020 (30)	184.305-588.190 per outcome	European	-	-	94 SNPs, ADH1B rs1229984	GSCAN SSGAC	Stroke,CHD,AF,HF,PAD	MEGASTROKE Consortium, UKB, ISGC, (CARDIoGRAMplusC4D) consortium, AFGen Consortium	**CHD** OR = 1.16 (1.00 - 1.36)<br>**AF** OR = 1.17 (1.00 - 1.37)<br>**PAD** OR = 3.05 (1.92 - 4.85)<br>**HF** OR = 1.00 (0.68 - 1.47)<br>**Stroke** OR = 1.27 (1.12 - 1.45)
Jiang et al,2020 (31)	60,620 cases;970,216 controls	European	-	-	43 SNPs	GSCAN ; GWAS	AF	Meta-analysis of 6 studies : Nord-Trøndelag Health Study, deCODE, MGI, DiscovEHR, UKB, AFGen Consortium	OR $_{drinks/week}$ = 1.00 (0.77 - 1.32)

Study	*Sample size*	*Ancestry*	*Sex*	*Mean age*	*IV*	*Exposure dataset*	*Outcome*	*Outcome dataset*	*Results Association with genetically predicted alcohol consumption*
Van Oort et al, 2020 (32)	FinnGen Study (15 870 cases and 74 345 controls) ; UKB (54 358 cases and 408 652 controls)	European	-	-	85 SNPs (alcohol dependance)	GWAS	HT	UKB;FinnGen consortium	OR =1.10 (1.06 - 1.13)
Christensen et al,2018 (33)	74,632	European	45% men	57	ADH1B rs1229984, ADH1C rs698	-	Stroke	CGPS;CCHS	HR = 1.15 (0.66 - 2.02)
Cho et al , 2015 (34)	7152	Asian	47% men	52±8	ALDH2 rs671	-	CHD,CVD,HT	KoGES, the Ansung and Ansan urban cohorts	**CHD** :<br>OR g/day = 0.98 (0.89 - 1.09),p =0.762<br>**HT** :<br>OR g/day = 1.031 (1.001 - 1.062), p= 0.040<br>**CVD** :<br>OR g/day = 0.949 (0.988 - 1.004), p= 0.362
Rosoff et al,2020 (35)	Range from 36,041 to 972,032	European	-	-	71 SNPs	UKB	Stroke, MI, CHD, HF	CARDIoGRAMplusC4D, HUNT, deCODE, MGI, DiscovEHR, UKB, AFGen consortium, ISGC, MEGASTROKE study	**MI** OR = 1.24 (1.03 - 1.50),p = 0.02<br>**CHD** OR 1.21 (1.01 - 1.45),p = 0.04<br>**HF** OR= 1.10 (0.9 - 1.3),p =0.3<br>**Stroke** OR= 1.13 (0.9 - 1.3),p =0.2
Au Yeung et al,2013 (36)	4867	Asian	Only men	≥ 50	ALDH2 rs671	GBCS	CVD,IHD	GBCS	**IHD** OR per 10g/day = 1.10 (0.83 - 1.45)<br>**CVD** OR per 10g/day = 0.98 (0.76 - 1.27)
Im et al.2023 (69)	12,176 (stroke)<br>8,408 (IHD)	Asian	41% men	52	ALDH2-rs671 ; ADH1B-rs1229984	CKB	Stroke<br>IHD	CKB	**Stroke** HR per 280g/week = 1.38 (1.27−1.49),p =6.8 × 10−15<br>**IHD** HR per 280g/week =1.04 (0.94−1.14), p= 0.457

Study	*Sample size*	*Ancestry*	*Sex*	*Mean age*	*IV*	*Exposure dataset*	*Outcome*	*Outcome dataset*	*Results Association with genetically predicted alcohol consumption*
Biddinger et al.2022 (37)	371 463	European	46% men	57	AUD , 9 SNPs AUDIT-C ,13 SNPs	The Mass General Brigham Biobank	HT,CAD,MI, stroke, heart failure, AF	The Mass General Brigham Biobank	**HT** OR = 1.28 (1.18 - 1.39)<br>**CAD** OR = 1.38 (1.10 - 1.74)<br>**MI** OR = 1.37 (1.05 - 1.78)<br>**Stroke** OR = 1.26 (1.04 -1.54)<br>**Heart failure** OR = 1.39 (1.08 - 1.78)<br>**AF** OR = 1.24 (1.08 -1.44)
Georgiou et al.2023 (38)	480 382	European	-	-	73 SNPs	Gwas meta-analysis for UKB, AFGen,CHARGE+ consortium	AF	AFGen	OR = 1.09 (0.72 – 1.76)
Lu et al.2021 (39)	65,446 AF cases ((84.2% European, 12.5% Japanese, 2.0% African American, 0.9% Brazilian and 0.4% Hispanic populations); 522744 controls	Mixed	-	-	6 SNPs associated with heavy alcohol consumption	UK Biobank	AF	AFGen consortium , Broad AF study , UK Biobank , Biobank Japan	OR=1.11 (1.04 – 1.18),p = 0.001
Yang et al.2022 (40)	8,964 (3,577 men for MR analysis)	Asian	Men	≈65.5	ALDH2 rs671	Dong-gu Study	AF	the Dong-gu Study	OR = 3.17 (1.18 – 9.24) ,p =0.028
Harshfield et al.2021 (41)	67 162 cases and 454 450 controls (MEGASTROKE), 1545 cases and 1481 controls (ISGC)	European	-	-	35 SNPs	UKB, 23andMe, deCODE, HUNT, WHI, COPDGene	Stroke : AS,AIS, CES, ICH , SVS	MEGASTROKE Consortium; ISGC	**AS** OR = 1.14 (0.97 - 1.34)<br>**AIS** OR = 1.12 (0.94 - 1.34)<br>**ICH** OR = 2.4 (0.71 - 8.08)<br>**CES** OR = 0.91 (0.67 - 1.24)<br>**LAS** OR = 1.04 (0.71 - 1.53)<br>**SVS** OR = 1.18 (0.79 - 1.76)

Study	Sample size	Ancestry	Sex	Mean age	IV	Exposure dataset	Outcome	Outcome dataset	Results Association with genetically predicted alcohol consumption
Yang et al.2023(42)	60,801 cases and 123,504 controls (CARDIoGRAMplusC4D), 39,036 cases and 303,463 controls (FinnGen)	European	-	-	84 SNPs	GSCAN	CHD	CARDIoGRAMplusC4D , Coronary Artery Disease (C4D) Genetics consortium, FinnGen study	$OR_{drinks/week}$ =1.11 (0.92 - 1.35),p = 0.267<br>$OR_{alcohol\ dependence}$ = 1.04 (1.02 - 1.06),p< 0.001
Hu et al.2022 (43)	17,676	Asian	Only men	52	ALDH2 rs671	ChinaMUCA 1998, InterASIA, ,CIMIC	CVD<br>CAD<br>Total stroke<br>Ischemic stroke<br>Hemorrhagic stroke	ChinaMUCA 1998, InterASIA, ,CIMIC	**CVD** HR =1.27 (1.05- 1.53),p =0.02<br>**CAD** HR =1.46 (1.01- 2.11),p=0.04<br>**Total stroke** HR =1.33 (1.02- 1.74),p =0.04<br>**Ischemic stroke** HR =1.22 (0.88- 1.69),p =0.24<br>**Hemorrhagic stroke** HR =1.46 (0.86 - 2.47),p =0.16
Jia et al.2022 (44)	CAD: 60,801 cases and 123,504 controls; Ischemic Stroke: 40,585 cases and 406,111 controls	European	-	-	89 SNPs :Alcohol consumption<br>2 SNPs :Alcohol dependance	MRC IEU OpenGWAS	CAD<br>Ischemic stroke	CARDIoGRAMplusC4D, MEGASTROKE	**Alcohol dependence/CAD** OR = 1.04 (1.03 - 1.06)<br>**Alcohol consumption/CAD** OR =1.18 (0.97 - 1.42)<br>**Alcohol consumption/Ischemic stroke** OR = 1.21 (0.97 - 1.50)<br>**Alcohol dependance/Ischemic stroke** OR = 0.99 (0.91 - 1.07)

Study	Sample size	Ancestry	Sex	Mean age	IV	Exposure dataset	Outcome	Outcome dataset	Results Association with genetically predicted alcohol consumption
Lankester et al.2021 (45)	337,484	European	-	67.6 (8)	ADH1B rs1229984 + 25 SNPs	UK Biobank. CARDIoGRAMplusC4D, MEGASTROKE, HERMES Heart Failure GWAS, 2018 AF HRC GWAS, FinnMetSeq exome sequence analysis	MI, stroke heart failure, AF	CARDIoGRAMplusC4D, MEGASTROKE, HERMES Heart Failure GWAS, 2018 AF HRC GWAS, FinnMetSeq exome sequence analysis	**Myocardial Infarction**<br>UKB, 2SLS: OR = 1.67 (1.28 - 2.18), # SNPs in IV = 1<br>UKB, IVW: OR = 1.29 (1.10 - 1.51), # SNPs in IV = 25<br>**Stroke**<br>UKB, 2SLS: OR = 1.61 (1.12 - 2.33), # SNPs in IV = 1<br>UKB, IVW: OR = 1.54 (1.21 - 1.95), # SNPs in IV = 25<br>**Heart Failure**<br>UKB, 2SLS: OR = 1.63 (1.06 - 2.50), # SNPs in IV = 1<br>UKB, IVW: OR = 1.25 (0.90 - 1.73), # SNPs in IV = 25<br>**Atrial Fibrillation**<br>UKB, 2SLS: OR = 1.40 (1.08 - 1.83), # SNPs in IV = 1<br>UKB, IVW: OR = 1.26 (1.01 - 1.57), # SNPs in IV = 25

Study	*Sample size*	*Ancestry*	*Sex*	*Mean age*	*IV*	*Exposure dataset*	*Outcome*	*Outcome dataset*	*Results Association with genetically predicted alcohol consumption*
Larsson et al.2024 (46)	7605 ICH cases ,711 818 controls	European	-	-	84 SNPs	GWAS meta-analysis (id=30643251)	Intracerebral haemorrage	FinnGen, UK Biobank, , Woo et al GWAS	**univariable analysis:** IVW OR=1,58 (1.07 - 2.35),p= 0,02 **FinnGen :** IVW，fixed-effect (Beta = 0.086, SE = 0.256, p =0.739) IVW， random-effects (Beta = 0.086, SE = 0.304, p = 0.778) **UK Biobank** IVW，fixed-effect (Beta = 0.912, SE = 0.298, p= 0.002) IVW， random-effects (Beta = 0.912, SE = 0.276, p = 0.001) **Woo et al. GWAS** IVW，fixed-effect (Beta = 0.688, SE = 0.673,p = 0.307) IVW， random-effects (Beta = 0.688, SE = 0.736, p =0.350)
Hisamtsu et al.2024 (47)	682	Asian	Only men	47 to 85 years	ALDH2-rs671	SESSA	Small vessel disease (SVD) large intracranial artery stenosis (ICAS)	SESSA	**Total SVDs** Age-adjusted OR = 1.46 (1.09 - 1.94) **ICAS** Age-adjusted OR = 0.70 (0.50 - 0.98) ,p =0.039
Tian et al.2022 (48)	10,754 cases and 306,882 controls	Mixed	-	-	53 SNPs	GWAS (PMID=30643251)	intracranial aneurysms	Gwas (doi: 10.1038/s41588-020-00760-4)	OR = 1.29 (0.68 - 2.45),p =0.43

ADH1B: Alcohol dehydrogenase 1B; ALDH2: Aldhyde dehydrogenase 2; AFGen =Atrial Fibrillation Genetics ;AMI= Acute Myocardial Infarction; AIS= any ischemic stroke; AS=all stroke; CES= cardioembolic stroke; BP: Blood Pressure; CI: Confidence interval; CVD: Cardiovascular Disease; CARDIoGRAMplusC4D= Meta-analysis of CARDIoGRAM and The Coronary Artery Disease Genetics; CHD: Coronary Heart Disease; COPDGene = Genetic Epidemiology of Chronic Obstructive Pulmonary Disease; ChinaMUCA= China Multi-Center Collaborative Study of Cardiovascular Epidemiology; CAD=Coronary artery disease; CIMIC =the Community Intervention of Metabolic Syndrome in China & Chinese Family HealthStudy; DBP: Diastolic Blood Pressure; GBCS: The Guangzhou Biobank Cohort Study; GWAS: Genome-wide Association Study; HR: Hazard Ratio; HR: Heart rate; HT: Hypertension;HF : Heart failure ; IHD: Ischemic Heart Disease; ISGC= International Stroke Genetics Consortium.; ICH =intracerebral hemorrhage ; InterASIA = the International Collaborative Study of Cardiovascular Disease in Asia ;HUNT = Nord-Trøndelag Health Study; ISGC=International Stroke Genetics Consortium; J-SHIPP= Shimanami Health Promoting Program; LAS= large artery stroke; MI= Myocardial Infarction; OR= Odds Ratio; SBP= Systolic Blood Pressure; SESSA= The Shiga Epidemiological Study of Subclinical Atherosclerosis; SNP= Single Nucleotide Polymorphism; SVS =small vessel stroke; UKB= UK Biobank; WHI = Women's Health Initiative

Supplementary Table 12. Characteristics and results of included mendelian randomization studies investigating genetically predicted alcohol consumption and brain diseases

Study	*Sample size*	*Ancestry*	*Sex*	*Mean age*	*IV*	*Exposure dataset*	*outcomes*	*Outcome dataset*	*Results Association with alcohol consumption*
Almeida et al,2014 (24)	3542	Mixed	Only men	71,3	ADH1B rs1229984	HIMS	Cognitive impairment	HIMS	AA/GG OR = 1.35, 95% CI = 0.29–6.27; GA/GG OR = 1.05, 95% CI = 0.71–1.55
Kumari et al,2014 (50)	34,452	Mixed	50% women,50% men	44-66	ADH1B rs1229984	HAPIEE Czech Republic,HAPIEE Russia,HAPIEE Poland,HAPIEE Lithuania,Whitehall II,ELSA	Cognitive function	HAPIEE Czech Republic,HAPIEE Russia,HAPIEE Poland,HAPIEE Lithuania,Whitehall II,ELSA	Any drinking versus none :Immediate recall −0.74 SD (−1.88 - 0.41)<br>Delayed recall −1.09 SD (−2.38 - 0.21)<br>Verbal fluency −0.63 SD (−1.78 - 0.53)<br>Processing speed −0.16 SD (−1.29 - 0.97)
Ritchie et al,2014 (51)	777	European	543 of population are women	10.94 (during the Scottish Mental Survey of 1947) 69.53 (between 2004 and 2007)	ADH1A rs2866151; ADH1A rs975833; ADH7 rs284779 ;ADH1B rs4147536	the Lothian Birth Cohort 1936	Cognitive ability	the Lothian Birth Cohort 1936	Alcohol consumption × SNP score interaction b= −1.13 (−1.94 ; −0.31),p = 0.007
Mahedy et al,2021(52)	~2500	European	-	24	87 SNPs	GWAS	Cognitive function: Working memory, Response inhibition, Emotion recognition	ALSPAC	**Working memory** b = 0.29 (-0.42 - 0.99),p = 0.43<br>**Response inhibition** b = -0.32 (-1.04 - 0.39),p = 0.38<br>**Emotion recognition** b = 0.03 (-0.55 - 0.61),p = 0.93
Andrews et al,2020 (53)	14,406 AD cases ,25,849 controls	European	60.35% women	77.49	44 SNPs (alcohol consumption),22 SNPs (alcohol dependence),11 SNPs (AUDIT scores)	UKB , GSCAN Consortium in Psychiatric Genomics	LOAD ,AAOS	EADI, ADGC, CHARGE	**LOAD/Alcohol consumption** OR = 0.96 (0.74 - 1.25),p = 0.775<br>**LOAD/Alcohol dependence** OR = 0.98 (0.93 - 1.04), p = 0.588<br>**LOAD/AUDIT scores** OR = 0.45 (0.12 - 1.75), p = 0.25<br>**AAOS/Alcohol consumption** HR = 2.02 (1.42 - 2.87), p = 9.4 × 10−05
Au Yeung et al,2012 (54)	5606	Asian	Only men	-	ALDH2 rs671	GBCS	Cognitive function: delayed 10-word recall score , MMSE score	GBCS	**Delayed 10-word recall score** b=-0.03 (-0.18 - 0.13),p = 0.61<br>**MMSE score** b= 0.06 (-0.22 - 0.34),p =0.55

Study	Sample size	Ancestry	Sex	Mean age	IV	Exposure dataset	outcomes	Outcome dataset	Results Association with alcohol consumption
Yu et al,2020 (55)	20,806 ALS cases ;59,804 controls	European	-	-	46 SNPs	GWAS,UKB	ALS	GWAS	OR = 2.48 (1.38 – 4.44),p = 0.002
Domínguez-Baleón et al.2022 (56)	941,280 for drinks per week	European	-	-	33 SNPs	GSCAN	Parkinson disease	IPDGC	OR = 0.79 (0.65 – 0.96; p = 0.021 bidirectional MR analysis IVW OR = 1.006 (0.999 – 1.013),p = 0.083
Heilbron et al.2021(57)	19,924 PD cases ; 2,413,087 controls	European	-	-	129 SNPs (cohort 1) 124 SNPs (cohort 2)	23andMe IPDGC	Parkinson disease	23andMe IPDGC	OR = 1.125 (1.025 – 1.235),p = 0.013
Zhang et al.2022 (58)	15,212 cases; 29,677 controls (ILAE) and 4588 cases and 144,780 controls (Finnegen)	European	-	-	84 SNPS	GSCAN	Epilepsy	ILAE FinnGen consortium	OR = 1.22 (1.02 – 1.45),p = 0.028
Yuan et al.2021 (59)	FinnGen consortium: 4,588 epilepsy cases and 144,780 controls. UK Biobank: 901 epilepsy cases and 395,209 controls.	European	-	-	79 SNPs	Gwas	Epilepsy	FinnGen consortium; UK Biobank	OR = 1.54 (0.99 – 2.41),p = 0.058
Domenighetti et al.2022 (60)	7,369 cases and 7,018 controls	European	-	67	ADH1B rs1229984/ 64 SNPs	GSCAN ;Courage-PD consortium	Parkinson's Disease	Courage-PD consortium	OR IVW = 0.68 (0.39 – 1.18), p = 0.17 OR Waldratio = 0.93 (0.45 –1.91),p = 0.85
Meng et al.2024 (61)	21,982 AD cases and 41,944 controls	European	-	-	89 SNPs for alcohol intake frequency; 30 SNPs for alcoholic drinks per week	UKB	Alzheimer disease	IGAP meta-analysis	$OR_{\text{Alcohol intake frequency}}$ = 0.923 (0.753 –1.134),p = 0.364 $OR_{\text{Alcoholic drinks per week}}$ = 1.162 (0.803 –1.678),p = 0.479
Wang et al.2022 (62)	9,064 HD patients	European	-	-	Drinks per week: 99 SNPs Grams per day: 46 SNP	GSCAN GWAS meta-analysis (Evangelou et al.)	Age at onset (AAO) of Huntington's disease (HD)	GWAS meta-analysis ofGenetic Modifiers of Huntington's Disease (GeM-HD) consortium	$OR_{\text{Drinks per week}}$ = 0.89 (-1.05 - 2.83),p=0.369 $OR_{\text{g/day}}$ = 0.86 (-3.73 - 5.46),p =0.712

Study	Sample size	Ancestry	Sex	Mean age	IV	Exposure dataset	outcomes	Outcome dataset	Results Association with alcohol consumption
Li et al.2024 (70)	**AD**: 21,982 cases and 41,944 controls.<br>**PD**: 33,674 cases and 449,056 controls.<br>**ALS**: 27,205 cases and 110,881 controls.<br>**MS**: 47,429 cases and 68,374 controls	European	-	-	**Drinks per week**<br>• **AD**: 28 SNPs<br>• **PD**: 30 SNPs<br>• **ALS**: 35 SNPs<br>• **MS**: 30 SNPs<br>**Alcohol Intake Frequency**<br>• **AD**: 88 SNPs<br>• **PD**: 91 SNPs<br>• **ALS**: 91 SNPs<br>• **MS**: 83 SNPs	GSCAN<br>MRC-IEU	Alzheimer's Disease (AD)<br>Parkinson's Disease (PD)<br>Amyotrophic Lateral Sclerosis (ALS)<br>Multiple Sclerosis (MS)	**AD**: International Genomics of Alzheimer's Project (IGAP) Stage 1 meta-analysis<br>**PD**: International Parkinson's Disease Genomics Consortium (IPDGC) meta-GWAS<br>**ALS**: Large-scale multi-source meta-GWAS<br>**MS** : International Multiple Sclerosis Genetics Consortium (IMSGC) meta-GWAS	**Drinks per Week**<br>• **AD**: b=−0.364, SE = 0.204, p=0.0745<br>• **PD**: b=−0.095b = -0.095b=−0.095, SE = 0.285, p=0.7381<br>• **ALS**: b=0.162, SE = 0.134, p=0.2295<br>• **MS**: b=0.092, SE = 0.235, p=0.6960<br>**Alcohol Intake Frequency**<br>• **AD**: b=−0.054, SE = 0.086, p=0.5252<br>• **PD**: b=−0.323, SE = 0.120, p=0.00717<br>• **ALS**: b=−0.047, SE = 0.064, p=0.4638<br>• **MS**: b=0.345, SE = 0.114, p=0.002418
Larsson et al. 2017(63)	17 008 cases of Alzheimer's disease and 37 154 controls	European	-	-	2 SNPs	IGAP	Alzheimer's Disease	IGAP	OR = 0.72 (0.50 - 1.04),p = 0.08
Grover et al.2019 (64)	9,581 parkinson cases and 33,245 controls	European	-	-	70 SNPs for drinks per week	UK Biobank	Parkinson disease	meta-analysis of GWAS	OR drinks/wk = 1.15 (0.87–1.53),p = 0.325

AAOS= Onset of Alzheimer's disease ;AD= Alzheimer disease ;ADGC= Alzheimer Disease Genetics Consortium ;ALDH2= Aldhyde dehydrogenase 2 ;ALS= Amyotrophic lateral sclerosis ;ALSPAC= Avon Longitudinal Study of Parents and Children ;AUDIT= Alcohol Use Disorder Identification Test ;CHARGE= Cohorts for Heart and Aging Research in Genomic Epidemiology ;ELSA= English Longitudinal Study of Ageing ;GBCS= The Guangzhou Biobank Cohort Study ;GSCAN= Sequencing Consortium of Alcohol and Nicotine use ;GWAS= Genome-wide Association Study ;HIMS= Health in Men Study ;HR= Hazard Ratio ; iPDGC= International Parkinson's Disease Genomics Consortium ;LOAD= Late-onset Alzheimer's disease ; ICAS= large intracranial artery stenosis; IGAP= The International Genomics of Alzheimer's Project ; IMSGC=International Multiple Sclerosis Genetics Consortium ;MR= Mendelian Randomization ;MR-PRESSO= Mendelian Randomization Pleitropy RESidual Sum and Outlier ; MRC-IEU= MRC Integrative Epidemiology Unit ;OR= Odds Ratio ;SNP= Single Nucleotide Polymorphism ; SVDs= small-vessel diseases; SESSA= Shiga Epidemiological Study of Subclinical Atherosclerosis;UKB= UK Biobank; ILAE= International League Against Epilepsy Consortium

Supplementary Table 13. Characteristics and results of included mendelian randomization studies investigating genetically predicted alcohol consumption and liver diseases

Study	*Sample size*	*Ancestry*	*Sex*	*Mean age*	*IV*	*Exposure dataset*	*outcomes*	*Outcome dataset*	*Results Association with alcohol consumption*
Lawlor et al,2014 (65)	58,313	European	-	>20	ADH1B rs1229984, ADH1C rs698	CGPS	Liver function: ALT, γ-GT, ALP, action of bilirubin ,prothrombin	CGPS	**ALT** (mean difference per doubling of alcohol consumption) 3.7% (–4.5, 11.9)<br>**γ-GT** (mean difference per doubling of alcohol consumption) 6.8 % (–2.8, 16.5)<br>**ALP** (mean difference per doubling of alcohol consumption) 11.6% (6.8, 16.4)<br>**Bilirubin** (mean difference per doubling of alcohol consumption) –2.4 (–9.4, 4.7)<br>**Prothrombin** (mean difference per doubling of alcohol consumption) –1.8 (–5.3, 1.7)
Carter et al.,2019 (66)	91 552	European	45.2% women	58 [13.05]	ADH1B rs1229984	CGPS	Plasma biomarkers of liver injury :ALT , γ-GT Incident cases of liver disease	CGPS	**ALT** OR = 0.47 (-0.36 - 1.29)<br>**GGT** OR = 0.88 (-0.1 - 1.86)<br>**Incident liver disease** OR = 1.71 (1.38 - 2.13)
Sookian et al,2016 (67)	466 : 331 NAFLD cases ,135 controls	European	Healthy subjects: 62 women,38 men ;patients with NAFLD: 65 women, 35 men	Healthy subjects:48±12 Patients with NAFLD:53±11	ADH1B rs1229984	Cohort study	NAFLD	Cohort study	Histological steatosis (1.76 ± 0.83 vs. 2.19 ± 0.78, p = 0.03) lobular inflammation (0.54 ± 0.65 vs. 0.95 ± 0.92, p = 0.02) NAFLD-Activity Score (2.9 ± 1.4 vs. 3.7 ± 1.4, p = 0.015)
Yuan et al.2022 (68)	discovery dataset (8434 NAFLD cases ;770,180 non-cases), replication dataset (1483 NAFLD cases ; 17,781 non-cases)	European	-	-	84 SNPs	GSCAN (id=30,643,251)	NAFLD	eMERGE , UK Biobank, Estonian Biobank, FinnGen	IVW combined analysis : OR = 0.61 (0.38 - 0.96), p =0.03

Study	Sample size	Ancestry	Sex	Mean age	IV	Exposure dataset	outcomes	Outcome dataset	Results Association with alcohol consumption
Im et al.2023 (69)	499 cases	Asian	41% men	52	ALDH2-rs671 ; ADH1B-rs1229984	CKB	Liver cirrhosis	CKB	HR per 280 g per week = 2.30 (1.58 − 3.35),p = 1.5 × 10−5
Yuan et al.2023 (10)	**Non-alcoholic fatty liver disease:** 3242 cases,707,631 controls **Alcoholic liver disease**: 2955 cases;680,369 controls **Cirrhosis**: 5904 cases,706,200 controls	European	-	-	84 SNPs	GWAS	Non-alcoholic fatty liver disease<br>Alcoholic liver disease<br>Cirrhosis	UK Biobank, FinnGen Study, International Inflammatory Bowel Disease Genetics Consortium (IIBDGC), Genetic Epidemiology Research on Aging (GERA)	**Non-alcoholic fatty liver disease** OR = 1.20 (0.63 - 2.28);p =0.574<br>**Alcoholic liver disease** OR = 14.35 (7.69 - 26.81);p =6.32 × 10-17<br>**Cirrhosis** OR = 2.96 (1.50 - 5.85) ;p =0.002

ADH1B = Alcohol dehydrogenase 1B ;ALDH2 = Aldehyde dehydrogenase 2 ;ALP = Alkaline phosphatase ;ALT = Alanine transaminase ;CI=Confidence interval; eMERGE =The Electronic Medical Records and Genomics; GBCS = The Guangzhou Biobank Cohort Study ;GGT = Gamma-glutamyl Transferase ;GPSC = General Population Survey of Copenhagen ;MR = Mendelian Randomization ;MR-PRESSO = Mendelian Randomization Pleiotropy RESidual Sum and Outlier ;NAFLD = Non-Alcoholic Fatty Liver Disease ;OR = Odds Ratio , SNP = Single Nucleotide Polymorphism

Supplementary References

1. Gormley M, Dudding T, Sanderson E, Martin RM, Thomas S, Tyrrell J, et al. A multivariable Mendelian randomization analysis investigating smoking and alcohol consumption in oral and oropharyngeal cancer. Nat Commun [Internet]. déc 2020 [cité 22 févr 2022];11(1):6071. Disponible sur: http://www.nature.com/articles/s41467-020-19822-6

2. Zhu J, Jiang X, Niu Z. Alcohol consumption and risk of breast and ovarian cancer: A Mendelian randomization study. Cancer Genet [Internet]. juill 2020 [cité 22 févr 2022];245:35-41. Disponible sur: https://linkinghub.elsevier.com/retrieve/pii/S2210776220302465

3. Larsson SC, Carter P, Kar S, Vithayathil M, Mason AM, Michaëlsson K, et al. Smoking, alcohol consumption, and cancer: A mendelian randomisation study in UK Biobank and international genetic consortia participants. Tsilidis KK, éditeur. PLOS Med [Internet]. 23 juill 2020 [cité 22 févr 2022];17(7):e1003178. Disponible sur: https://dx.plos.org/10.1371/journal.pmed.1003178

4. Brunner C, Davies NM, Martin RM, Eeles R, Easton D, Kote-Jarai Z, et al. Alcohol consumption and prostate cancer incidence and progression: A Mendelian randomisation study: Alcohol metabolism and prostate cancer. Int J Cancer [Internet]. 1 janv 2017 [cité 22 févr 2022];140(1):75-85. Disponible sur: https://onlinelibrary.wiley.com/doi/10.1002/ijc.30436

5. Ong J, Derks EM, Eriksson M, An J, Hwang L, Easton DF, et al. Evaluating the role of alcohol consumption in breast and ovarian cancer susceptibility using population-based cohort studies and two-sample Mendelian randomization analyses. Int J Cancer [Internet]. 15 mars 2021 [cité 22 févr 2022];148(6):1338-50. Disponible sur: https://onlinelibrary.wiley.com/doi/10.1002/ijc.33308

6. Chen C, Hu Q, Wang J, Wen T, Zhu C, Tan W, et al. Habitual consumption of alcohol with meals and lung cancer: a Mendelian randomization study. Ann Transl Med [Internet]. févr 2021 [cité 22 févr 2022];9(3):263-263. Disponible sur: https://atm.amegroups.com/article/view/58549/html

7. Zhou X, Wang L, Xiao J, Sun J, Yu L, Zhang H, et al. Alcohol consumption, DNA methylation and colorectal cancer risk: Results from pooled cohort studies and Mendelian randomization analysis. Int J Cancer [Internet]. juill 2022 [cité 4 juill 2024];151(1):83-94. Disponible sur: https://onlinelibrary.wiley.com/doi/10.1002/ijc.33945

8. Zhou X, Yu L, Wang L, Xiao J, Sun J, Zhou Y, et al. Alcohol consumption, blood DNA methylation and breast cancer: a Mendelian randomisation study. Eur J Epidemiol [Internet]. juill 2022 [cité 4 juill 2024];37(7):701-12. Disponible sur: https://link.springer.com/10.1007/s10654-022-00886-1

9. Liu S, Feng S, Du F, Zhang K, Shen Y. Association of smoking, alcohol, and coffee consumption with the risk of ovarian cancer and prognosis: a mendelian randomization study. BMC Cancer [Internet]. 20 mars 2023 [cité 8 juill 2024];23(1):256. Disponible sur: https://bmccancer.biomedcentral.com/articles/10.1186/s12885-023-10737-1

10. Yuan S, Chen J, Ruan X, Sun Y, Zhang K, Wang X, et al. Smoking, alcohol consumption, and 24 gastrointestinal diseases: Mendelian randomization analysis. eLife [Internet]. 2 févr 2023 [cité 8 juill 2024];12:e84051. Disponible sur: https://elifesciences.org/articles/84051

11. Deng Y, Huang J, Wong MCS. Associations between six dietary habits and risk of hepatocellular carcinoma: A Mendelian randomization study. Hepatol Commun [Internet]. août 2022 [cité 8 juill 2024];6(8):2147-54. Disponible sur: https://journals.lww.com/10.1002/hep4.1960

12. Liu Z, Song C, Suo C, Fan H, Zhang T, Jin L, et al. Alcohol consumption and hepatocellular carcinoma: novel insights from a prospective cohort study and nonlinear Mendelian randomization analysis. BMC Med [Internet]. 28 oct 2022 [cité 14 juill 2024];20(1):413. Disponible sur: https://bmcmedicine.biomedcentral.com/articles/10.1186/s12916-022-02622-8

13. Chen F, Wen W, Long J, Shu X, Yang Y, Shu X, et al. Mendelian randomization analyses of 23 known and suspected risk factors and biomarkers for breast cancer overall and by molecular subtypes. Int J Cancer [Internet]. août 2022 [cité 9 juill 2024];151(3):372-80. Disponible sur: https://onlinelibrary.wiley.com/doi/10.1002/ijc.34026

14. Zou M, Liang Q, Zhang W, Zhu Y, Xu Y. Causal association between dietary factors and esophageal diseases: A Mendelian randomization study. Andl CD, éditeur. PLOS ONE [Internet]. 29 nov 2023 [cité 9 juill 2024];18(11):e0292113. Disponible sur: https://dx.plos.org/10.1371/journal.pone.0292113

15. He M, Huan L, Wang X, Fan Y, Huang J. Nine dietary habits and risk of colorectal cancer: a Mendelian randomization study. BMC Med Genomics [Internet]. 17 janv 2024 [cité 10 juill 2024];17(1):21. Disponible sur: https://bmcmedgenomics.biomedcentral.com/articles/10.1186/s12920-023-01782-7

16. Yang J, Qu X, Zheng A jie, Jiang F, Chang H, Zhang J ru, et al. The causal effects of genetically predicted alcohol consumption on endometrial cancer risk from a Mendelian randomization study. Sci Rep [Internet]. 12 févr 2024 [cité 10 juill 2024];14(1):3478. Disponible sur: https://www.nature.com/articles/s41598-024-53926-z

17. Cai X, Li X, Liang C, Zhang M, Dong Z, Yu W. The effect of metabolism-related lifestyle and clinical risk factors on digestive system cancers in East Asian populations: a two-sample Mendelian randomization analysis. Sci Rep [Internet]. 24 avr 2024 [cité 11 juill 2024];14(1):9474. Disponible sur: https://www.nature.com/articles/s41598-024-60122-6

18. Zhang B, Peng Y, Luo Y, Hong C, Lin Y, Zhang Y, et al. Relationship between esophageal squamous cell carcinoma risk and alcohol-related ALDH2 and ADH1B polymorphisms: Evidence from a META-ANALYSIS and Mendelian randomization analysis. Cancer Med [Internet]. oct 2023 [cité 14 juill 2024];12(20):20437-49. Disponible sur: https://onlinelibrary.wiley.com/doi/10.1002/cam4.6610

19. Zhang H, Liu J. Lifestyle factors, glycemic traits, and lipoprotein traits and risk of liver cancer: a Mendelian randomization analysis. Sci Rep [Internet]. 12 avr 2024 [cité 15 juill 2024];14(1):8502. Disponible sur: https://www.nature.com/articles/s41598-024-59211-3

20. Xu J, Liu W, Liu X, Zhou X, Li G. Alcohol drinking, smoking, and cutaneous melanoma risk: Mendelian randomization analysis. Gac Sanit [Internet]. 2023 [cité 15 juill 2024];37:102351. Disponible sur: https://linkinghub.elsevier.com/retrieve/pii/S0213911123000729

21. Li Y, Ye D, Zhou W, Liu B, Mao Y, Sun X. Alcohol consumption and colorectal cancer risk: A mendelian randomization study. Front Genet [Internet]. 23 sept 2022 [cité 16 juill 2024];13:967229. Disponible sur: https://www.frontiersin.org/articles/10.3389/fgene.2022.967229/full

22. Ding J, Tu Z, Chen H, Liu Z. Identifying modifiable risk factors of lung cancer: Indications from Mendelian randomization. Zhao JV, éditeur. PLOS ONE [Internet]. 18 oct 2021 [cité 16 juill 2024];16(10):e0258498. Disponible sur: https://dx.plos.org/10.1371/journal.pone.0258498

23. Tan Y, Wei Z, Liu K, Qin Y, Hui W. Lifestyle habits and gastric cancer in an East Asian population: a Mendelian randomization study. Front Oncol [Internet]. 4 sept 2023 [cité 16 juill 2024];13:1224753. Disponible sur: https://www.frontiersin.org/articles/10.3389/fonc.2023.1224753/full

24. Wang X, Bi Y, Liu G, Wang W, Cui H. Smoking and alcohol consumption with the risk of 11 common otolaryngological diseases: a bidirectional Mendelian randomization. Eur Arch Otorhinolaryngol [Internet]. déc 2023 [cité 16 juill 2024];280(12):5615-23. Disponible sur: https://link.springer.com/10.1007/s00405-023-08246-9

25. Xiong J, Yang L, Deng Y, Yan S, Gu J, Li B, et al. The causal association between smoking, alcohol consumption and risk of bladder cancer: A univariable and multivariable Mendelian randomization study. Int J Cancer [Internet]. 15 déc 2022 [cité 17 juill 2024];151(12):2136-43. Disponible sur: https://onlinelibrary.wiley.com/doi/10.1002/ijc.34228

26. van Oort S, Beulens JWJ, van Ballegooijen AJ, Handoko ML, Larsson SC. Modifiable lifestyle factors and heart failure: A Mendelian randomization study. Am Heart J [Internet]. sept 2020 [cité 22 févr 2022];227:64-73. Disponible sur: https://linkinghub.elsevier.com/retrieve/pii/S0002870320301873

27. Zhao PP, Xu LW, Sun T, Wu YY, Zhu XW, Zhang B, et al. Relationship between alcohol use, blood pressure and hypertension: an association study and a Mendelian randomisation study. J Epidemiol Community Health [Internet]. sept 2019 [cité 22 févr 2022];73(9):796-801. Disponible sur: https://jech.bmj.com/lookup/doi/10.1136/jech-2018-211185

28. Holmes MV, Dale CE, Zuccolo L, Silverwood RJ, Guo Y, Ye Z, et al. Association between alcohol and cardiovascular disease: Mendelian randomisation analysis based on individual participant data. BMJ [Internet]. 10 juill 2014 [cité 22 févr 2022];349(jul10 6):g4164-g4164. Disponible sur: https://www.bmj.com/lookup/doi/10.1136/bmj.g4164

29. Millwood IY, Walters RG, Mei XW, Guo Y, Yang L, Bian Z, et al. Conventional and genetic evidence on alcohol and vascular disease aetiology: a prospective study of 500 000 men and women in China. The Lancet [Internet]. mai 2019 [cité 21 févr 2022];393(10183):1831-42. Disponible sur: https://linkinghub.elsevier.com/retrieve/pii/S0140673618317720

30. Larsson SC, Burgess S, Mason AM, Michaëlsson K. Alcohol Consumption and Cardiovascular Disease: A Mendelian Randomization Study. Circ Genomic Precis Med [Internet]. juin 2020 [cité 22 févr 2022];13(3). Disponible sur: https://www.ahajournals.org/doi/10.1161/CIRCGEN.119.002814

31. Jiang Q, Wang K, Shi J, Li M, Chen M. No association between alcohol consumption and risk of atrial fibrillation: A two-sample Mendelian randomization study. Nutr Metab Cardiovasc Dis [Internet]. juill 2020 [cité 22 févr 2022];30(8):1389-96. Disponible sur: https://linkinghub.elsevier.com/retrieve/pii/S0939475320301356

32. van Oort S, Beulens JWJ, van Ballegooijen AJ, Grobbee DE, Larsson SC. Association of Cardiovascular Risk Factors and Lifestyle Behaviors With Hypertension: A Mendelian Randomization Study. Hypertension [Internet]. déc 2020 [cité 22 févr 2022];76(6):1971-9. Disponible sur: https://www.ahajournals.org/doi/10.1161/HYPERTENSIONAHA.120.15761

33. Christensen AI, Nordestgaard BG, Tolstrup JS. Alcohol Intake and Risk of Ischemic and Haemorrhagic Stroke: Results from a Mendelian Randomisation Study. J Stroke [Internet]. 31 mai 2018 [cité 22 févr 2022];20(2):218-27. Disponible sur: http://j-stroke.org/journal/view.php?doi=10.5853/jos.2017.01466

34. Cho Y, Shin SY, Won S, Relton CL, Davey Smith G, Shin MJ. Alcohol intake and cardiovascular risk factors: A Mendelian randomisation study. Sci Rep [Internet]. 22 déc 2015 [cité 22 févr 2022];5(1):18422. Disponible sur: http://www.nature.com/articles/srep18422

35. Rosoff DB, Davey Smith G, Mehta N, Clarke TK, Lohoff FW. Evaluating the relationship between alcohol consumption, tobacco use, and cardiovascular disease: A multivariable Mendelian randomization study. Gill D, éditeur. PLOS Med [Internet]. 4 déc 2020 [cité 22 févr 2022];17(12):e1003410. Disponible sur: https://dx.plos.org/10.1371/journal.pmed.1003410

36. Au Yeung SL, Jiang C, Cheng KK, Cowling BJ, Liu B, Zhang W, et al. Moderate Alcohol Use and Cardiovascular Disease from Mendelian Randomization. Biondi-Zoccai G, éditeur. PLoS ONE [Internet]. 16 juill 2013 [cité 22 févr 2022];8(7):e68054. Disponible sur: https://dx.plos.org/10.1371/journal.pone.0068054

37. Biddinger KJ, Emdin CA, Haas ME, Wang M, Hindy G, Ellinor PT, et al. Association of Habitual Alcohol Intake With Risk of Cardiovascular Disease. JAMA Netw Open [Internet]. 25 mars 2022 [cité 13 juin 2024];5(3):e223849. Disponible sur: https://jamanetwork.com/journals/jamanetworkopen/fullarticle/2790520

38. Georgiou A, Georgiopoulos G, Delialis D, Maneta E, Masci PG, Neophytou O, et al. Causal Relationship Between Average Alcohol Consumption and Risk of Atrial Fibrillation: A Mendelian Randomization Study. Circ Genomic Precis Med [Internet]. août 2023 [cité 14 juin 2024];16(4):406-8. Disponible sur: https://www.ahajournals.org/doi/10.1161/CIRCGEN.122.003766

39. Lu Y, Guo Y, Lin H, Wang Z, Zheng L. Genetically determined tobacco and alcohol use and risk of atrial fibrillation. BMC Med Genomics [Internet]. déc 2021 [cité 15 juin 2024];14(1):73. Disponible sur: https://bmcmedgenomics.biomedcentral.com/articles/10.1186/s12920-021-00915-0

40. Yang JH, Jeong JA, Kweon SS, Lee YH, Choi SW, Ryu SY, et al. Causal Association Between Alcohol Consumption and Atrial Fibrillation: A Mendelian Randomization Study. Korean Circ J [Internet]. 2022 [cité 15 juin 2024];52(3):220. Disponible sur: https://e-kcj.org/DOIx.php?id=10.4070/kcj.2021.0269

41. Harshfield EL, Georgakis MK, Malik R, Dichgans M, Markus HS. Modifiable Lifestyle Factors and Risk of Stroke: A Mendelian Randomization Analysis. Stroke [Internet]. mars 2021 [cité 15 juin 2024];52(3):931-6. Disponible sur: https://www.ahajournals.org/doi/10.1161/STROKEAHA.120.031710

42. Yang F, Xu F, Zhang H, Gill D, Larsson SC, Li X, et al. Proteomic insights into the associations between obesity, lifestyle factors, and coronary artery disease. BMC Med [Internet]. 5 déc 2023 [cité 16 juin 2024];21(1):485. Disponible sur: https://bmcmedicine.biomedcentral.com/articles/10.1186/s12916-023-03197-8

43. Hu C, Huang C, Li J, Liu F, Huang K, Liu Z, et al. Causal associations of alcohol consumption with cardiovascular diseases and all-cause mortality among Chinese males. Am J Clin Nutr [Internet]. sept 2022 [cité 16 juin 2024];116(3):771-9. Disponible sur: https://linkinghub.elsevier.com/retrieve/pii/S0002916522000910

44. Jia Y, Wang R, Guo D, Sun L, Shi M, Zhang K, et al. Contribution of metabolic risk factors and lifestyle behaviors to cardiovascular disease: A mendelian randomization study. Nutr Metab Cardiovasc Dis [Internet]. août 2022 [cité 17 juin 2024];32(8):1972-81. Disponible sur: https://linkinghub.elsevier.com/retrieve/pii/S0939475322001909

45. Lankester J, Zanetti D, Ingelsson E, Assimes TL. Alcohol use and cardiometabolic risk in the UK Biobank: A Mendelian randomization study. Taniyama Y, éditeur. PLOS ONE [Internet]. 11 août 2021 [cité 17 juin 2024];16(8):e0255801. Disponible sur: https://dx.plos.org/10.1371/journal.pone.0255801

46. Larsson SC, Chen J, Gill D, Burgess S, Yuan S. Risk Factors for Intracerebral Hemorrhage: Genome-Wide Association Study and Mendelian Randomization Analyses. Stroke [Internet]. juin 2024 [cité 12 juin 2024];55(6):1582-91. Disponible sur: https://www.ahajournals.org/doi/10.1161/STROKEAHA.124.046249

47. Hisamatsu T, Tabara Y, Kadota A, Torii S, Kondo K, Yano Y, et al. Alcohol Consumption and Cerebral Small- and Large-Vessel Diseases: A Mendelian Randomization Analysis. J Atheroscler Thromb [Internet]. 1 févr 2024 [cité 24 juin 2024];31(2):135-47. Disponible sur: https://www.jstage.jst.go.jp/article/jat/31/2/31_64222/_article

48. Tian D, Zhang L, Zhuang Z, Huang T, Fan D. A two-sample Mendelian randomization analysis of modifiable risk factors and intracranial aneurysms. Sci Rep [Internet]. 10 mai 2022 [cité 2 juill 2024];12(1):7659. Disponible sur: https://www.nature.com/articles/s41598-022-11720-9

49. Almeida OP, Hankey GJ, Yeap BB, Golledge J, Flicker L. Alcohol consumption and cognitive impairment in older men: A mendelian randomization study. Neurology [Internet]. 25 mars 2014 [cité 22 févr 2022];82(12):1038-44. Disponible sur: https://www.neurology.org/lookup/doi/10.1212/WNL.0000000000000255

50. Kumari M, Holmes MV, Dale CE, Hubacek JA, Palmer TM, Pikhart H, et al. Alcohol consumption and cognitive performance: a M endelian randomization study. Addiction [Internet]. sept 2014 [cité 22 févr 2022];109(9):1462-71. Disponible sur: https://onlinelibrary.wiley.com/doi/10.1111/add.12568

51. Ritchie SJ, Bates TC, Corley J, McNeill G, Davies G, Liewald DC, et al. Alcohol consumption and lifetime change in cognitive ability: a gene × environment interaction study. AGE [Internet]. juin 2014 [cité 22 févr 2022];36(3):9638. Disponible sur: http://link.springer.com/10.1007/s11357-014-9638-z

52. Mahedy L, Suddell S, Skirrow C, Fernandes GS, Field M, Heron J, et al. Alcohol use and cognitive functioning in young adults: improving causal inference. Addiction [Internet]. févr 2021 [cité 22 févr 2022];116(2):292-302. Disponible sur: https://onlinelibrary.wiley.com/doi/10.1111/add.15100

53. Andrews SJ, Goate A, Anstey KJ. Association between alcohol consumption and Alzheimer's disease: A Mendelian randomization study. Alzheimers Dement [Internet]. févr 2020 [cité 22 févr 2022];16(2):345-53. Disponible sur: https://onlinelibrary.wiley.com/doi/10.1016/j.jalz.2019.09.086

54. Au Yeung SL, Jiang CQ, Cheng KK, Liu B, Zhang WS, Lam TH, et al. Evaluation of Moderate Alcohol Use and Cognitive Function Among Men Using a Mendelian Randomization Design in the Guangzhou Biobank Cohort Study. Am J Epidemiol [Internet]. 15 mai 2012 [cité 22 févr 2022];175(10):1021-8. Disponible sur: https://academic.oup.com/aje/article-lookup/doi/10.1093/aje/kwr462

55. Yu X, Wang T, Chen Y, Shen Z, Gao Y, Xiao L, et al. Alcohol Drinking and Amyotrophic Lateral Sclerosis: An Instrumental Variable Causal Inference. Ann Neurol [Internet]. juill 2020 [cité 22 févr 2022];88(1):195-8. Disponible sur: https://onlinelibrary.wiley.com/doi/10.1002/ana.25721

56. Domínguez-Baleón C, Ong JS, Scherzer CR, Rentería ME, Dong X. Understanding the effect of smoking and drinking behavior on Parkinson's disease risk: a Mendelian randomization study. Sci Rep [Internet]. 7 juill 2021 [cité 12 juin 2024];11(1):13980. Disponible sur: https://www.nature.com/articles/s41598-021-93105-y

57. Heilbron K, Jensen MP, Bandres-Ciga S, Fontanillas P, Blauwendraat C, Nalls MA, et al. Unhealthy Behaviours and Risk of Parkinson's Disease: A Mendelian Randomisation Study. J Park Dis [Internet]. 12 oct 2021 [cité 12 juin 2024];11(4):1981-93. Disponible sur: https://www.medra.org/servlet/aliasResolver?alias=iospress&doi=10.3233/JPD-202487

58. Zhang Z, Wang M, Yuan S, Liu X. Alcohol, Coffee, and Milk Intake in Relation to Epilepsy Risk. Nutrients [Internet]. 9 mars 2022 [cité 13 juin 2024];14(6):1153. Disponible sur: https://www.mdpi.com/2072-6643/14/6/1153

59. Yuan S, Tomson T, Larsson SC. Modifiable risk factors for epilepsy: A two-sample Mendelian randomization study. Brain Behav [Internet]. mai 2021 [cité 2 juill 2024];11(5):e02098. Disponible sur: https://onlinelibrary.wiley.com/doi/10.1002/brb3.2098

60. Domenighetti C, Sugier PE, Sreelatha AAK, Schulte C, Grover S, Mohamed O, et al. Mendelian Randomisation Study of Smoking, Alcohol, and Coffee Drinking in Relation to Parkinson's Disease. J Park Dis [Internet]. 21 janv 2022 [cité 2 juill 2024];12(1):267-82. Disponible sur: https://www.medra.org/servlet/aliasResolver?alias=iospress&doi=10.3233/JPD-212851

61. Meng Q, Chen C, Zhu M, Huang Y. Dietary factors and Alzheimer's disease risk: a Mendelian randomization study. Eur J Med Res [Internet]. 2 mai 2024 [cité 3 juill 2024];29(1):261. Disponible sur: https://eurjmedres.biomedcentral.com/articles/10.1186/s40001-024-01821-8

62. Wang M, Liu D, Yang S, Li Y, Lian X. Smoking, alcohol consumption, and age at onset of Huntington's disease: a Mendelian randomization study. Parkinsonism Relat Disord [Internet]. avr 2022 [cité 3 juill 2024];97:34-8. Disponible sur: https://linkinghub.elsevier.com/retrieve/pii/S1353802022000475

63. Larsson SC, Traylor M, Malik R, Dichgans M, Burgess S, Markus HS. Modifiable pathways in Alzheimer's disease: Mendelian randomisation analysis. BMJ [Internet]. 6 déc 2017 [cité 23 juill 2024];j5375. Disponible sur: https://www.bmj.com/lookup/doi/10.1136/bmj.j5375

64. Grover S, Lill CM, Kasten M, Klein C, Del Greco M F, König IR. Risky behaviors and Parkinson disease: A mendelian randomization study. Neurology [Internet]. 8 oct 2019 [cité 23 juill 2024];93(15). Disponible sur: https://www.neurology.org/doi/10.1212/WNL.0000000000008245

65. Lawlor DA, Benn M, Zuccolo L, De Silva NMG, Tybjaerg-Hansen A, Smith GD, et al. ADH1B and ADH1C Genotype, Alcohol Consumption and Biomarkers of Liver Function: Findings from a Mendelian Randomization Study in 58,313 European Origin Danes. Sookoian SC, éditeur. PLoS ONE [Internet]. 15 déc 2014 [cité 22 févr 2022];9(12):e114294. Disponible sur: https://dx.plos.org/10.1371/journal.pone.0114294

66. Carter AR, Borges MC, Benn M, Tybjærg-Hansen A, Davey Smith G, Nordestgaard BG, et al. Combined Association of Body Mass Index and Alcohol Consumption With Biomarkers for Liver Injury and Incidence of Liver Disease: A Mendelian Randomization Study. JAMA Netw Open [Internet]. 8 mars 2019 [cité 22 févr 2022];2(3):e190305. Disponible sur: http://jamanetworkopen.jamanetwork.com/article.aspx?doi=10.1001/jamanetworkopen.2019.0305

67. Sookoian S, Flichman D, Castaño GO, Pirola CJ. Mendelian randomisation suggests no beneficial effect of moderate alcohol consumption on the severity of nonalcoholic fatty liver disease. Aliment Pharmacol Ther [Internet]. déc 2016 [cité 22 févr 2022];44(11-12):1224-34. Disponible sur: https://onlinelibrary.wiley.com/doi/10.1111/apt.13828

68. Yuan S, Chen J, Li X, Fan R, Arsenault B, Gill D, et al. Lifestyle and metabolic factors for nonalcoholic fatty liver disease: Mendelian randomization study. Eur J Epidemiol. juill 2022;37(7):723-33.

69. Im PK, Wright N, Yang L, Chan KH, Chen Y, Guo Y, et al. Alcohol consumption and risks of more than 200 diseases in Chinese men. Nat Med [Internet]. juin 2023 [cité 8 juin 2024];29(6):1476-86. Disponible sur: https://www.nature.com/articles/s41591-023-02383-8

70. Li D, Zhou L, Cao Z, Wang J, Yang H, Lyu M, et al. Associations of environmental factors with neurodegeneration: An exposome-wide Mendelian randomization investigation. Ageing Res Rev [Internet]. mars 2024 [cité 3 juill 2024];95:102254. Disponible sur: https://linkinghub.elsevier.com/retrieve/pii/S1568163724000722
